# Cell‐to‐cell and type‐to‐type heterogeneity of signaling networks: insights from the crowd

**DOI:** 10.15252/msb.202110402

**Published:** 2021-10-18

**Authors:** Attila Gabor, Marco Tognetti, Alice Driessen, Jovan Tanevski, Baosen Guo, Wencai Cao, He Shen, Thomas Yu, Verena Chung, Single Cell Signaling in Breast Cancer DREAM Consortium members, Bernd Bodenmiller, Julio Saez‐Rodriguez

**Affiliations:** ^1^ Institute for Computational Biomedicine Heidelberg University and Heidelberg University Hospital Faculty of Medicine Bioquant Heidelberg Germany; ^2^ Department of Quantitative Biomedicine & Institute of Molecular Life Sciences University of Zurich Zurich Switzerland; ^3^ Institute of Molecular Systems Biology ETH Zurich Zurich Switzerland; ^4^ Molecular Life Science PhD Program Life Science Zurich Graduate School ETH Zurich and University of Zurich Zurich Switzerland; ^5^ Division of AI & Bioinformatics Shenzhen Digital Life Institute Shenzhen China; ^6^ Sage Bionetworks Seattle WA USA

**Keywords:** cell signaling, crowdsourcing, mass cytometry, predictive modeling, single cell, Cancer, Computational Biology, Signal Transduction

## Abstract

Recent technological developments allow us to measure the status of dozens of proteins in individual cells. This opens the way to understand the heterogeneity of complex multi‐signaling networks across cells and cell types, with important implications to understand and treat diseases such as cancer. These technologies are, however, limited to proteins for which antibodies are available and are fairly costly, making predictions of new markers and of existing markers under new conditions a valuable alternative. To assess our capacity to make such predictions and boost further methodological development, we organized the Single Cell Signaling in Breast Cancer DREAM challenge. We used a mass cytometry dataset, covering 36 markers in over 4,000 conditions totaling 80 million single cells across 67 breast cancer cell lines. Through four increasingly difficult subchallenges, the participants predicted missing markers, new conditions, and the time‐course response of single cells to stimuli in the presence and absence of kinase inhibitors. The challenge results show that despite the stochastic nature of signal transduction in single cells, the signaling events are tightly controlled and machine learning methods can accurately predict new experimental data.

## Graphical abstract

This study presents the results and conclusions of the ‘Single Cell Signaling in Breast Cancer DREAM challenge’, where teams were challenged to use state‐of‐the‐art methods for predicting single‐cell signaling from single‐cell and bulk proteomics, transcriptomics and genomics data.

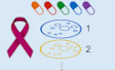

## Introduction

Cell signaling governs most activities of cells as it underlies the ability to correctly respond to stimuli from the cellular microenvironment. This dynamic process of signaling is tightly regulated by the interactions of proteins. Deregulation and major disturbances in this finely tuned machinery can lead to diseases such as cancer (Hynes & MacDonald, 2009), autoimmunity (Benveniste *et al*, 2014), and diabetes (Boucher *et al*, 2014).

The abundance of signaling proteins and the mutation status of underlying genes vary across cell lines; therefore, different cell lines have distinct signaling networks. But not only different cell lines have different signaling networks: Cells of the same type display dissimilar signaling patterns in response to stimuli depending on history, cell state, and microenvironment. This heterogeneity has important implications for the treatment of diseases. For example, intratumor heterogeneity in cancer is a key contributor to therapeutic failure and drug resistance: Often subpopulations do not respond to therapies, resulting in relapses. Thus, a better understanding of signaling and its heterogeneity might unlock better treatments (Yaffe, 2019).

Single‐cell measurements, particularly mass cytometry (Bodenmiller *et al*, 2012; Spitzer & Nolan, 2016), have opened a new way to monitor the signaling in individual cells. With this technique, we can measure the cellular response to multiple perturbations, paving the way for understanding the heterogeneity of the underlying cellular mechanisms.

However, the measured nodes are limited to a handful of proteins for fluorescence‐based live cell imaging and several dozens for highly multiplexed mass cytometry. If we could predict nodes that are not measured via computational approaches, we could overcome this technological limitation. In addition, the number of combinatorial treatments in studies is often limited by the budget or available material, since these experiments are complicated and expensive. Therefore, it would also be desirable whether models could predict the single‐cell response of a cell line to new treatments after training on response data of other cell lines. One could even conceive models that predict the response to perturbations from only basal, unperturbed data.

Answering these questions requires advanced computational methods. Predictive mathematical models are often used for bulk data analysis (Byrne *et al*, 2016; Rukhlenko *et al*, 2018), but they have not yet been applied extensively to single‐cell data (Loos *et al*, 2018). Therefore, as a first step, we need to assess the available modeling frameworks for single‐cell predictions on a fair platform and explore their use, capabilities, and also limits.

To accelerate the development of methods, we organized the Single Cell Signaling in Breast Cancer (SCSBrC) DREAM challenge. The Dialogue for Reverse Engineering Assessments and Methods (DREAM) Challenges provides a framework for participants across the globe to compare their methods for solving biomedical problems (Saez‐Rodriguez *et al*, 2016). The participants are ranked according to predefined metrics, and the solutions are analyzed to find the best ways to approach the particular problems. Further, the predictions, the methods, and the code that reproduce the results are made publicly available after the challenge as a stepping stone for further improvements. DREAM challenges have enabled the benchmark and development of methods in systems biology (Meyer & Saez‐Rodriguez, 2021), including the inference of signaling networks (Prill *et al*, 2011; Hill *et al*, 2016).

**Figure 1:**
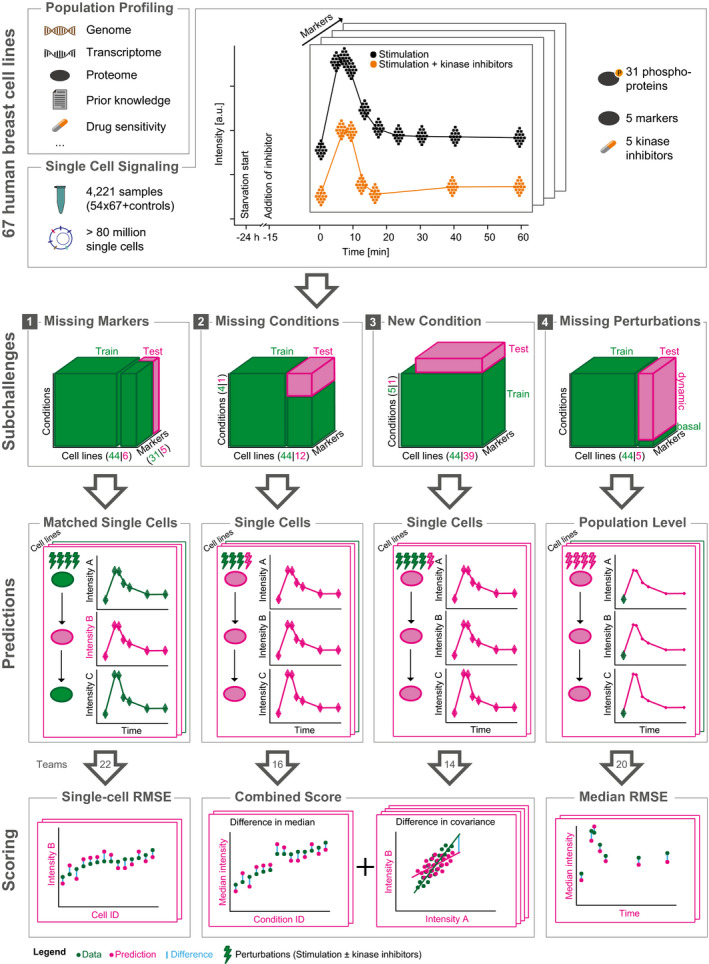
Overview of the Single Cell Signaling in Breast Cancer challenge The challenge data consist of genomic, basal transcriptomic, and proteomic data, and perturbation single‐cell phosphorylation datasets from 67 breast cancer cell lines. The datasets were divided into training (green; given to participants) and test (pink; withhold for scoring). The training data were common but test data different for each subchallenge. In each subchallenge, a suitable metric was chosen to score the predictions.

We based the SCSBrC challenge on a single‐cell signaling mass cytometry dataset (Tognetti *et al*, 2021). In this dataset, 67 cell lines were stimulated with EGF and serum (referred hereafter as EGF treatment) in combination with one of five kinase inhibitors (PKC, PI3K, mTOR, MEK, and EGFR), and in each condition, 31 phosphoproteins and 5 cellular markers were measured at 10 different time points over the course of one hour (Fig 1, Appendix Fig S1 and Appendix Fig S2A). In total, this dataset includes more than 80 million single cells and more than 4,000 experimental conditions. The data are complemented with population‐level protein abundance experiments, transcriptomic data (RNAseq), and genomic data (Single Nucleotide Polymorphism and Copy Number Aberration; Marcotte *et al*, 2016). A portion of this data was kept for scoring (test data), and the rest was provided as training data to the participants to develop their methods. Further, we encouraged participants to incorporate external data and resources of prior knowledge, such as pathways and protein–protein interaction databases, in their models.

Specifically, in our challenge we aimed to answer the following questions of increasing complexity: “Can we predict the signaling response (i) of nodes that are not directly measured from other measurements on the same cells?”, (ii) “to new combinatorial treatments based on how other cell lines respond to that treatment?”, (iii) “to perturbations for which we have no data but know the target?”, and (iv) purely from basal omics data?”. We defined four corresponding subchallenges (Fig 1).

**Figure 2:**
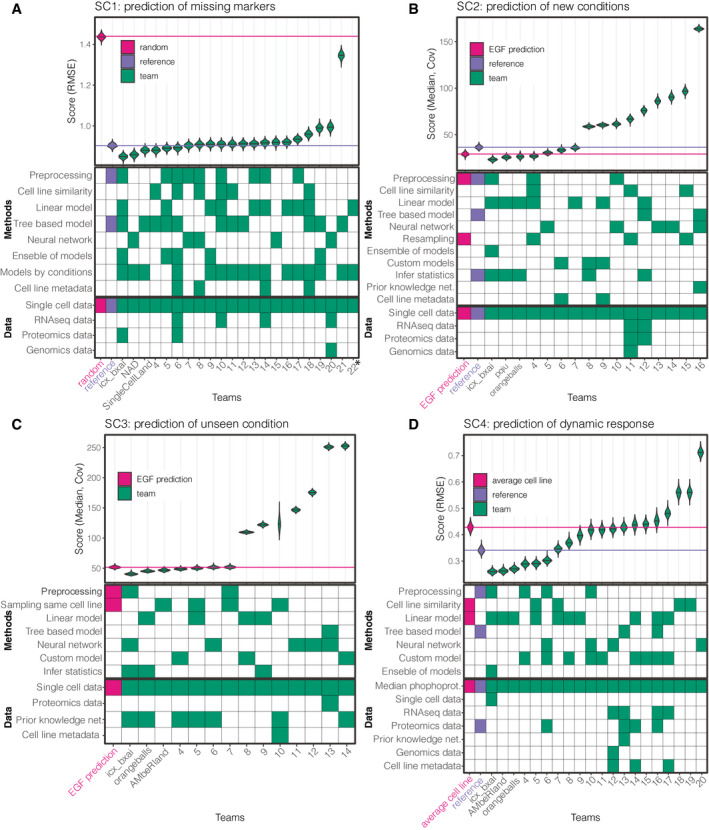
Summary of the ranking of teams and their methods and data usage across subchallenges A–DA, B, C, and D summarize subchallenge 1–4, respectively. The violin plots show the distribution of the scores obtained on bootstrap samples of the conditions in the test data. In all challenges, lower scores mean better predictions. Reference models that we built before the challenge are highlighted with blue color, and random and null models (EGF condition‐based predictions and average cell line‐based predictions) are shown with magenta. The tile‐plots summarize the approach and data used by each team. Data types that are used by at least one team are shown in each subchallenge. *Team 22 in SC1 scored worse than random, and their score exceeds the limit of the y‐axis.

We evaluated 73 models from 27 teams across the four subchallenges and found that machine learning‐based methods worked well for these prediction tasks (Fig 2). Subchallenge 1 showed that the signaling dynamics in nodes that are measured in some cell lines can be accurately predicted in other cell lines. In some cell lines, the basal phosphorylation of the nodes is significantly different from others, which may lead to a shift between measurements and predictions; therefore, we recommend to include the basal phosphorylation in the predictive models. Subchallenge 2 showed that the effect of kinase inhibitors can be learnt from other cell lines and predicted with high accuracy. Predictions performed better than random even when only the target of the kinase inhibitor is given (Subchallenge 3). Further, we found major differences between model types in terms of performance at capturing intra‐cell line heterogeneity. Finally, we found in Subchallenge 4 that models can partially predict the dynamics of a cell line from the basal omics if one uses the general signaling response of other cell lines.

## Results

The SCSBrC DREAM challenge was organized in three rounds between September and December 2019. A total of 261 individuals registered, and 22, 16, 14, and 20 teams around the world submitted predictions in the final round of each subchallenge, respectively. The teams were ranked in each subchallenge (SC1‐4) based on their predictions on the test dataset (Fig 2A–D, respectively). The challenges were followed by a post‐challenge period where participants submitted write‐ups and code, and participated in a survey about their methods. The experimental data, models, and results are freely available for further use (https://www.synapse.org/singlecellproteomics).

### The missing marker prediction subchallenge

The goal of subchallenge 1 (SC1) was to build a model that can predict the phosphorylation of a node that one may not be able to measure experimentally. To obtain such models, we asked participants to predict the phosphorylation of selected proteins in single cells from specific cell lines and conditions (pink box; Fig 1). To do this, the participants could use the other nodes of the signaling network for the same condition and cell lines as well as the same nodes from different cell lines (green box; Fig 1; Appendix Fig S1). Specifically, we asked the participants to predict five markers (p‐ERK^Thr202/Tyr‐204^, p‐AKT^Ser473^, p‐PLCg2^Tyr759^, p‐HER2^Tyr1196^, and p‐S6^Ser235/Ser236^) in six cell lines, measured in all the 49 different conditions, totaling 11.9 million cells. The participants submitted their predictions for each measured single cell, and we compared them to the actual measurements of the markers using the root mean square error (RMSE) (see Materials and Methods and Fig 1). This performance was also compared against a reference model we built before the challenge using a random forest predictor (see Materials and Methods).

### Quality of predictions

First, we ranked the teams and compared their performance with random predictions and with a random forest‐based reference (see Materials and Methods, Appendix Table S4). For robust ranking, we used bootstrap sampling of the test conditions to generate a distribution of scores for each team (see Materials and Methods; Fig 2A). This identified the winner (*icx_bxai*, RMSE_icx_bxai_ = 0.849), which strongly outperformed other participants (Bayes factor > 499). For the random prediction, we took the test data and shuffled the unique cell‐ids in each cell line and experimental condition. This way, when we scored this randomized dataset, each cell was predicted by a randomly chosen other cell in the same cell line and experimental condition. Almost all teams performed better than this random prediction (Fig 2A), with 32% average improvement (RMSE_teams average_ = 0.979 ± 0.238, RMSE_random_ = 1.44 ± 2.6e‐4). Six teams achieved better predictions than a reference model that we built before the challenge started (RMSE_reference_ = 0.903, see Materials and Methods).

**Figure 3:**
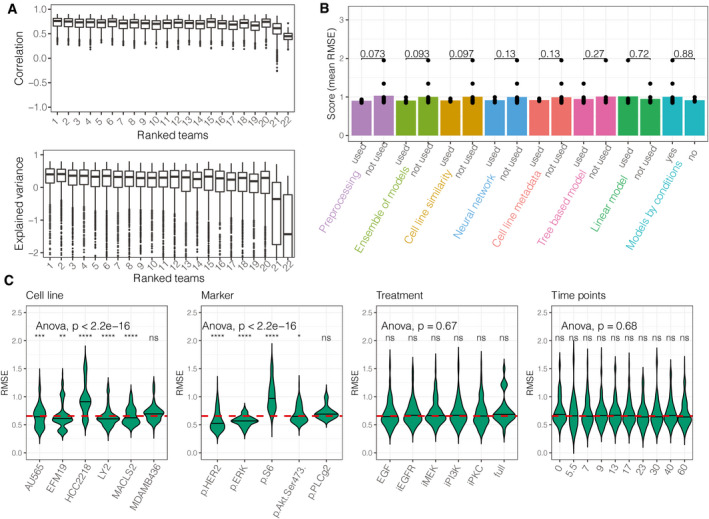
Summary of subchallenge 1 Distribution of the correlation coefficients and explained variances across conditions, cell lines, and markers (*N*_data_ = 1,165), for each team. The medians are indicated by thick lines, the 0.25 and 0.75 quantiles are indicated by the boxes. The whiskers extend up to the maximum/minimum, but no further than 1.5 * interquartile range. Values beyond the whiskers are plotted individually.Comparison of the performance of the teams using a specific method or dataset; individual performances are marked with dots (*N*_teams_ = 22). Numbers above the bars represent the *P*‐value from a *t*‐test.Distribution of the prediction errors for cell lines, markers, treatments, and time points. Each violin is built from the prediction errors of all teams. The dashed red horizontal line shows the global mean RMSE. The significance of a *t*‐test between the groups and the global RMSE is shown above each violin plot (*P < 0.05, **P < 0.01, ***P < 0.001, ****P < 0.0001, ns: not significant).

Models predicted the trend of the data well, but for a few cell lines the predictions under‐ or overestimated the test data. Model predictions correlated strongly with the test data (median correlation coefficient: 0.761) in each condition (Fig 3A top) and explained large amounts of variance (*R* ^2^_median_ = 0.40, *R* ^2^_max_ = 0.803) across all teams (Fig 3A bottom). The explained variance was negative in some conditions, which means that the predictions of node phosphorylation were worse than the sample mean in those conditions. These conditions were found to belong to specific cell lines that had strongly different initial activation (at time 0, before stimulating) of the predicted nodes than the majority of cell lines (details below), leading to a shift between predictions and measurements.

The training data did not contain measurements of the initial activation of the test cell lines. To quantify how much the knowledge of this initial phosphorylation influences the accuracy of the predictions, we corrected the predictions by the difference between the predicted and measured mean basal activities of the cell lines. All the predictions improved significantly (Appendix Fig S3D): in average teams’ error decreased by 10.4%, and even for the top team the error decreased by 7% (from RMSE_icx_bxai_ = 0.849 to RMSE_icx_bxai, corrected_ = 0.789). This suggests that it is very hard to predict phosphorylation without any data for the phospho‐protein of interest in the cell line where it has to be predicted.

We evaluated the winner’s team method and a random prediction on a similar, but independent mass cytometry dataset (Lun *et al*, 2017), where the authors studied the differences in signaling dynamics due to node abundance (Appendix section “Single cell prediction on independent dataset”). We used the winner’s method to learn signaling relationships among the measured markers in a set of cells overexpressing some proteins and then predict missing markers on cells expressing other proteins (Appendix Table S7). We found that the challenge winner’s method greatly outperformed a random prediction (RMSE_team, Lun_ = 0.508 vs. RMSE_random, Lun_ = 1.298; Appendix Fig S10), which demonstrates the applicability of the method on other datasets.

### What makes a good model for node prediction?

To understand what makes a model better than others, we analyzed the participants’ write‐up of methods and their answers to a questionnaire. The best three teams approached the prediction task with different methods: an ensemble of linear and tree‐based methods, convolutional neural networks, and gradient boosting, respectively (read about the details of each method in Appendix). Further, while teams 1 and 3 built models for each missing marker separately, the neural network model of team 2 predicted all the 5 missing markers together. Common among the three approaches are that all of them included all the 32 measured markers as model features among others and each team addressed the problem of missing data.

We compared the building blocks of the methods to see which elements contributed to better predictions (Fig 3B). Although we did not find a single model type or modeling procedure strikingly beneficial, several features improved the accuracy of predictions. In particular, pre‐processing of the data (mean RMSE improvement: 0.13, *P* = 0.0731 from a *t*‐test), using an ensemble of models (estimated RMSE improvement: 0.094, *P* = 0.0935), and training models on similar cell lines (improvement: 0.0949, *P* = 0.0975) had the largest individual impact on the score.

While some teams relied on a single method to build their models, the top‐ranked team (*icx_bxai*) built a model for each marker independently and included the 32 provided markers, treatment, and time, and the top principal components of the proteomic data as features. Further, when estimating the marker values at time *t*_i_, they also considered the median signaling levels of the 32 measured markers at time *t*_i_, *t*_i−1_, and *t*_i+1_. They used subsets of these features to train an ensemble of models: ElasticNet, ExtraTrees, RandomForest, Light Gradient Boosting, and linear model. To make a prediction, they averaged the predictions of all models.

The successful combination of methods by the best performers suggests that, as experienced in other DREAM challenges (Marbach *et al*, 2012; Saez‐Rodriguez *et al*, 2016), the combinations of methods provide robust predictors. We performed multiple aggregation of the predictions (see Materials and Methods and Appendix Fig S3C), but could not achieve significant improvement: The combination of the top two teams led to 0.5% improvement over the best team. This is probably due to the large correlation between the residuals of the teams predictions, which means that when a team overshot their predictions, other teams did similarly.

### Time‐course analysis of predictions

We investigated whether there were specific markers, conditions, or cell lines that are harder to predict than others (Fig 3C). Prediction errors distributed heterogeneously across the cell lines and markers (ANOVA test, *P* < 2.2e‐16; Appendix Table S1), but homogeneously across treatments (*P* = 0.67) and time after the perturbation (*P* = 0.68) (Fig 3C). In particular, *p‐Her2* was the most accurately predicted marker (improvement of 0.142 of the global mean RMSE; Appendix Table S2) and the largest prediction error was found for *p‐S6* (deterioration of 0.305). Cell line HCC2218 has the largest prediction errors (0.246 above global mean RMSE), while other cell lines had similarly small prediction error values. The prediction error differences between cell lines and markers suggest that the cell lines show diverse signaling patterns that influence the models’ accuracy (Appendix Fig S4).

**Figure 4:**
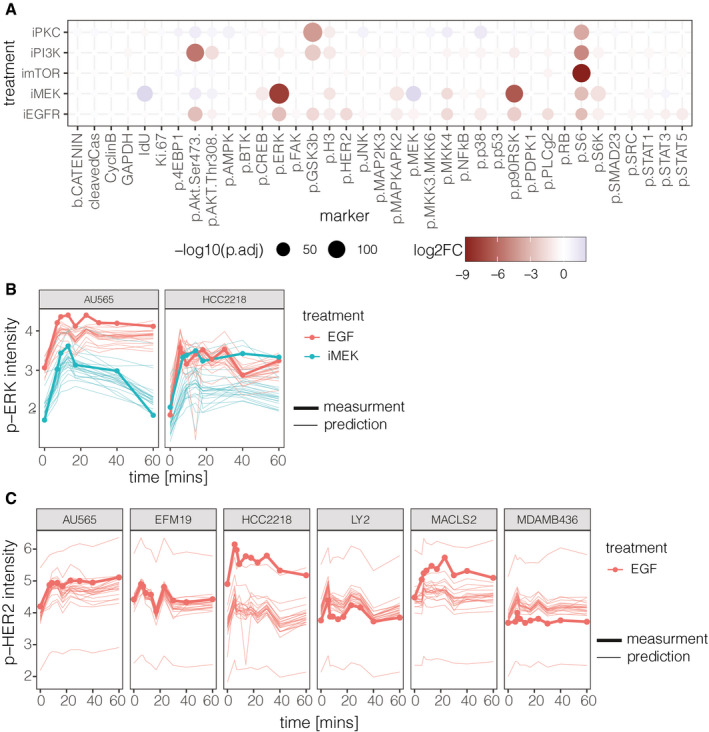
Effect of kinase inhibitors The population‐level effect of inhibitors on the phosphosites averaged across all 67 cell lines. (log_2_ fold change in signal level between cells treated with EGF + Serum and EGF + Serum in combination with a kinase inhibitor).Measured and predicted population‐level p‐ERK response to EGF and EGF + iMEK inhibitor.Measured and predicted population‐level p‐HER2 response to EGF and EGF + iEGFR inhibitor.

As signaling is a dynamic process, we aggregated the predictions of each team and the test data at each time point and compared the time‐courses. The dynamic response of phosphosites changed strongly across the different perturbations and also between cell lines (Appendix Fig S4), which was captured well by the predictions. For example, MDAMB436 cells showed strong p‐AKT activation upon all perturbations with EGF, except when a PI3K inhibitor (iPI3K) was applied. The majority of models captured both the initial dynamics and the level at which the phosphorylation saturated and predicted the low response of p‐AKT when iPI3K was added. The measured members of the mitogen‐activated protein kinase (MAPK) pathway (e.g., p‐MEK, p‐ERK, and downstream p‐p90RSK) show similar activation dynamics in response to EGF stimulations, which was accurately predicted by most participants (Fig 4B). For example, there is a strong decrease in p‐ERK when we compare its response between EGF and iMEK conditions (mean phosphorylation decreases: log_2_ fold change −7.66, adj. *P*‐value = 4.14e‐23; Fig 4A), which was also predicted well by most models (Fig 4B). However, in five cell lines (four training cell lines: HCC2185, HCC1599, DU4475, CAL148 and one test cell line HCC2218), the p‐ERK phosphorylation did not decrease after inhibiting MEK (Fig 4B), indicating a rare (observed for 5 out of 67 cell lines) MEK independent ERK signaling.

The estimates of p‐HER2 in HCC2218 cells also showed a large prediction error (Fig 4C). This is mostly due to the large shift in the initial (time 0) signal between the predictions and the data; HCC2218 shows higher activation of p‐HER2 than other cell lines in the test set. This also emphasizes the importance to measure and incorporate the basal phosphorylation of predicted markers in all cell lines in the modeling whenever possible.

In summary, this subchallenge shows that models can learn the signaling relationship of the markers on a single‐cell level and they can predict the response of individual markers. Further, the basal activation of the marker in the new cell line can improve these predictions and particular attention has to be paid on non‐canonical signaling effects.

### The missing conditions subchallenges

In SC2 and SC3, we challenged participants to predict the effect of known and new inhibitors. We asked them to estimate the response of populations of cells from selected cell lines to specific kinase inhibitors, i.e., all markers at all measured time points. This is a fundamentally different task from SC1, because all the nodes are unknown in the predicted condition, and therefore, the models have to capture the signaling relationships of all the markers to predict between conditions (Fig 1).

The task in SC2 was to predict the response to EGF stimulus in combination with four inhibitors (targeting EGFR, MEK, PI3K, and PKC; Appendix Table S3) in twelve test cell lines (Appendix Fig S2). The training data in SC2 contained the response of the other cell lines to these inhibitors, as well as the basal phosphorylation data for those cell lines. In contrast, in SC3 we asked the participants to predict the effect of an mTOR inhibitor, without giving them any training data for this specific condition. We only provided the name of the inhibitor and the concentration applied in the experiments (Appendix Table S3), and data upon treatment with other inhibitors on those cell lines. Since the effect of this inhibitor could not be observed from training data, participants were encouraged to use prior knowledge for this task.

**Figure 5:**
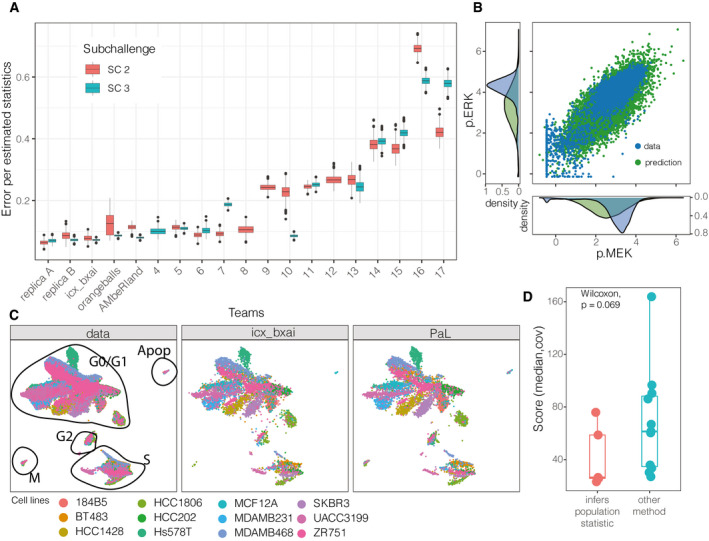
Summary of subchallenges 2 and 3 Comparison of teams’ predictions for SC2 and SC3 on a subset of data (15 mins of incubation with inhibitors, no stimuli); the distribution of prediction error is obtained via bootstrapping (*N*_samples_ = 1,000). In addition, the prediction error that one would obtain using either biological replica A or replica B as predictions is also shown on the subset of data that overlaps between both biological replicas. The medians are indicated by thick lines, and the 0.25 and 0.75 quantiles are indicated by the boxes. The whiskers extend up to the maximum/minimum, but no further than 1.5 * interquartile range. Values beyond the whiskers are plotted individually.Comparison of single‐cell predictions from winning team with the data in a single condition: test cell line MDAMB468, iEGFR perturbation, 7 min after treatment.Test data shown on two‐dimensional Uniform Manifold Approximation and Projection (UMAP) with annotated clusters that belong to different cell cycle phases (left); projection of the challenge winner’s prediction on the same manifold (middle), projection of the fourth team’s, sampling‐based prediction on the same manifold (right).Comparison of performance of teams using statistical inference and then applying Gaussian sampler against teams using other methods (*N*_teams_ = 17). The medians are indicated by thick lines, and the 0.25 and 0.75 quantiles are indicated by the boxes. The whiskers extend up to the maximum/minimum, but no further than 1.5 * interquartile range. Values beyond the whiskers are plotted individually.

The predictive models were scored and ranked based on 10,000 single‐cell predictions submitted for each target cell line and condition by each team. We scored the predictions using population‐level statistics; the mean value of each marker and the covariance between marker pairs (see Materials and Methods) in each condition. This way we capture not only the accuracy of individual markers, but also the changing interplay (covariance) between markers in the score (Fig 5B). A reference model was built using a random forest predictor before the challenge (see Materials and Methods).

### Quality of predictions

As for SC1, we ranked the participants based on bootstrap samples of the conditions (SC2: Fig 2B and SC3: Fig 2C) and compared the ranked predictions in SC2 to a reference model that we built before the challenge (Fig 2B, model described in Materials and Methods). Seven out of the 16 teams performed better than our reference model (RMSE_reference_ = 44.67), with up to 48% improvement (median improvement: 39.3%). Of note, a predictor corresponding to the lack of effect of the kinase inhibitor, thus using the measurements to the stimulus alone, leads to a score of RMSE_EGF_ = 29.156. This relatively good score could be due to the fact that each inhibitor showed strong effect only on a few nodes in the measured part of the signaling network. Surprisingly, only the top four teams managed to perform better (Fig 2B, Appendix Table S5).

A subset of the test data (all cell lines, 15 min after incubation with inhibitor, without EGF stimulation), contains two biological replicates (replica A and B). Their variation can be considered as an estimate of the upper‐bound of performance as it corresponds to the experimental error. Therefore, we also scored the participants on this subset of conditions and compared their scores to the variation of the measured individual biological replicas. Since SC2 and SC3 used different numbers of cell lines (12 and 38, respectively), we computed the prediction error per total number of predicted statistics. This allowed us to summarize the two subchallenges together (Fig 5A); however, they remain not directly comparable as the test cell lines and conditions were different. With that in mind, in SC2 the mean score of replica A and B (0.0753) is just slightly better than the top‐performing team (0.0782), which also holds for SC3, where the average score of the two biological replica is 0.0712 and the best team achieved 0.0725. This shows that the best team could predict single‐cell response with very good accuracy, close to that of a biological replica.

Similarly to SC1, we found that in both SC2 and SC3 the prediction error fluctuated across cell lines (ANOVA, *P* = 8.8e‐4), less so across treatments (*P* = 0.034) and not significantly across time (*P =* 0.54) (Appendix Fig S5A). The differences across cell lines were more prominent when only the top four teams, which performed better than the reference model, were considered (Appendix Fig S5B).

### Modeling approaches

Interestingly, five teams, including the top three (*icx_bxai*, *pqui*, and *orangeballs*), followed similar modeling approaches for SC2 (more about the approaches in the Appendix). These teams inferred the median phosphorylation level of the proteins and their covariance matrices for each sample (cell line, treatment, and time point) using linear models (Elastic‐net by *icx_bxai* and linear regression by *pqui* and *orangeballs*). Further, all three predicted the cells in the target conditions using the available conditions of the same cell line, rather than from the same conditions of other cell lines. In the next step, they sampled from a multidimensional Gaussian distribution that followed the inferred statistics in each condition which resulted in the single‐cell predictions. Despite all these similarities, the Z‐score transformation, handling the missing time points and using other statistical features of the single‐cell distributions (means, medians, and quantiles), led *icx_bxai* to the first place.

We also evaluated multiple scenarios for combining the predictions for SC2 (see Materials and Methods). The combination of top *N* teams led to 1–4.2% improvement over the top‐ranked team for the range of *N* = 2‐8 teams, and the best score was achieved with the top four teams (Appendix Fig S6). A random forest model trained on the predictions errors also performed similarly. The combination of any number of random teams occasionally achieved good scores and had a decreasing trend as the number of teams increased, but on average performed worse than simply assuming no inhibition effect.

### Intra‐cell line heterogeneity

The evaluation metric for SC2 and SC3 compared the predictions and data based on population‐level statistics (mean and covariance), but we were curious how well the predictions captured further single‐cell features. Therefore, we analyzed, for SC2, how the different methods generated heterogeneity and how this matched the heterogeneity found in the data (Fig 5C). We could distinguish two fundamentally different prediction methods. Five teams (including the top three teams) used methods that involve the estimation of population‐level statistics of the cells (mean and covariance) and then used a Gaussian sampler that generated single cells that followed the estimated statistics. Due to the Gaussian sampler, these approaches inherently assume homogeneity. These types of methods showed good performance: teams using this approach achieved on average 38.7% better score (Wilcoxon test, *P*‐value: 0.069) than teams relying on other methods (Fig 5D). Another popular method (3 teams) was to resample and then optionally scale the available training data (e.g., the 4th best team). This method does not rely on a similar assumption of normality, and thus manages to keep the underlying heterogeneity of the cells.

The measured cells show heterogeneity: The single‐cell measurements have the tendency of forming multiple clusters that are driven by the cell cycle states (see Materials and Methods), and where all the cell lines are represented in each main cluster (Fig 5C). The global layout of the predictions that are either based on Gaussian sampler (Fig 5C middle) or based on resampling and scaling real measurements (Fig 5C right) are in good agreement with the measurements (Fig 5C left). However, when we focused on cells in a single‐cell line and single treatment conditions (Appendix Fig S7), the heterogeneity due to cell cycle states was only visible in the resampling method because resampling inherits the heterogeneity of the sampled cell lines, while the Gaussian sampler‐based method predicted homogeneously distributed cells.

In summary, the participants predicted the time response of all markers in response to kinase inhibitors, first (SC2) to inhibitors with known effects and then (SC3) to an inhibitor which was absent from the training data. The results show that predicting distributions of single cells’ populations after perturbations with kinase inhibitors is almost as good as a biological replica and that, at least for some cases, this is possible even if no data are available upon perturbation with those specific inhibitors. The four best teams were better than the reference model, and the top team achieved an accuracy similar to a biological replica. Although overall machine learning‐based methods performed better, sampling methods were better able at capturing single‐cell heterogeneity.

### The time‐course prediction subchallenge

Perturbation data contain invaluable information about the directionality, magnitude, and timescale of the cell response. It also reveals causal interactions of nodes; therefore, it is often used for network inference (Zoppoli *et al*, 2010). However, do we need to do perturbation experiments on each and every cell line or is this information already available from the genome, basal protein expression and basal phosphorylation? We formulated subchallenge 4 (SC4) to investigate how well the dynamic response of cell lines can be predicted from their unperturbed, basal state. The participants had to predict the population‐level response (median of single cells) of five test cell lines to EGF stimulus alone and in combination to four kinase inhibitors across the measured time‐course (Appendix Fig S2). As in previous subchallenges, they were allowed to build their models based on the training cell lines data (for some of which data under all conditions are available) and use the basal omics (unperturbed, basal proteomics and phosphoproteomics and genomics) data of the test cell lines.

### Quality of the predictions compared with an average cell line

**Figure 6:**
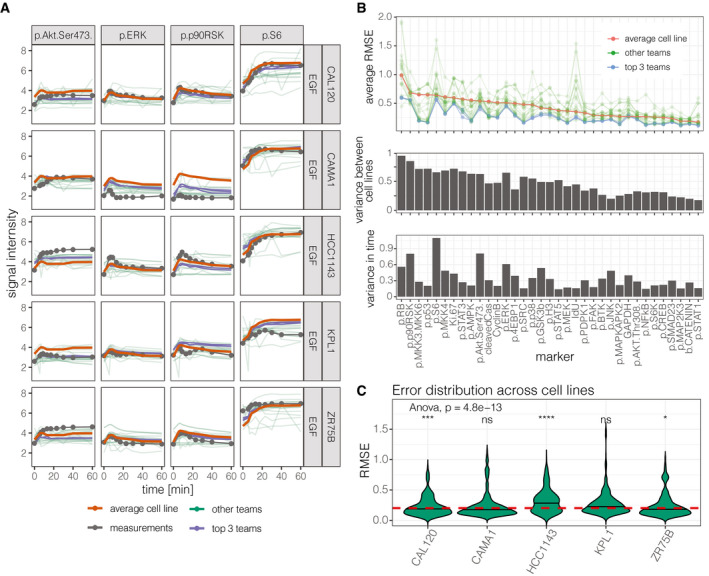
Prediction of dynamic response from basal omics Time‐course comparison of the dynamic response of the test cell lines with the average of the training cell lines and the teams' predictions (highlighting the top 3 teams).Comparison of the error of predictions and mean of the training cell lines for each marker (top), variance of each marker between cell lines, and mean variance of each marker in time.Comparison of prediction errors in each cell line. The red dashed horizontal line shows the global mean RMSE. The significance of a *t*‐test between the groups and the global RMSE is shown above each violin plot (*P < 0.05, **P < 0.01, ***P < 0.001, ****P < 0.0001, ns: not significant).

We ranked the predictions of the participants based on the RMSE (Fig 2D) and compared them with a reference prediction (model built before the challenge, see details in Materials and Methods) and with a baseline model in which we simply averaged all the training cell lines in each condition. Clearly, the baseline model captures the general trends (Fig 6A) and does not capture any cell line‐specific responses. Based on the scoring metric (RMSE), 11 of the 20 teams predicted better than the baseline model and the top team performed 40% better than the baseline (score of the baseline: 0.428, reference model: 0.3408, top team: 0.260).

The top three participants outperformed the average cell line model (baseline model) for all the markers (Fig 6B) except for *GAPHD*, for which their prediction error was similar to the average cell line model (prediction approaches are summarized in Appendix; statistical comparison in Appendix Table S6). Comparing the predictions with the baseline model, the RMSE was not the same for all markers: Certain markers, for example, *p.p53* and *p.MKK3/6*, were predicted much better by the top teams than by the average cell line model (Fig 6B).

To understand which markers can be modeled more accurately, we compared the marker variance across cell lines to the prediction errors (Fig 6B) and we found that the variance correlates strongly with the error in the average cell line (corr. coeff: 0.944), but less to the errors in the participants’ prediction (corr.coeff: 0.73). This means that the participants’ models performed better than the average cell line model, especially for the markers that changed strongly between cell lines.

Further, we also compared the prediction errors with the longitudinal variance of the markers (Fig 6B) that tells us how strongly the markers respond to the perturbation in time. The longitudinal variance correlated more with the participants error (corr. coeff: 0.858) than with the average cell line prediction error (corr. coeff: 0.501). We can also see that the participant prediction was much better than the average cell line model when the marker longitudinal variance was lower (Fig 6B), i.e., when the marker changed less in time.

Surprisingly, in contrast to SC1, the prediction errors were similar across cell lines. Although the distribution of the prediction error across the test cell lines showed statistically significant differences (ANOVA test, *P*‐value = 4.8e‐13; Fig 6C), the differences are not strong: The largest difference in RMSE (0.0528) was found between cell line HCC1143 and the mean cell line. This shows that modeling approaches generalizes well across cell lines.

In summary, in the last subchallenge, the goal was to predict the dynamics of the population response to perturbations from unperturbed basal data. Only 11 teams achieved better performance than random, underscoring the difficulty of the task. Unsurprisingly, predictions were particularly good for markers that did not change across time, but changed across cell lines.

## Discussion

New technologies allow us to interrogate cell signaling at the single‐cell level. New types of data come with new challenges for data analysis, and it is crucial to understand and tackle the limitations of existing computational methods. Toward this goal, we organized the Single Cell Signaling in Breast Cancer DREAM challenge to draw the attention of the computational community to single‐cell prediction problems, obtain state‐of‐the‐art solutions, and evaluate their performance. Our results provide a baseline for further development and a reference for the community to learn what approaches work best and what the limitations are.

The participants built predictive models from a rich and complex dataset, composed of the largest single‐cell signaling dataset to date and complementary bulk data types. In Subchallenge 1, the majority of the teams predicted missing nodes in the signaling network building models based on all the other measured nodes in each single cell and a summary (e.g., principal components) of the RNAseq and proteomic data. These types of models were trained on the cell lines provided and then used to predict the test cell lines. This implicitly assumes that the relationship among the nodes can be transferred between cell lines. This, in turn, could reflect common molecular programs driven by the shared lineage of the cell lines. We would thus expect less transferability when considering different tissues or processes that are less linked to each other as the ones considered here. The predictions strongly correlated with the test data and explained large amounts of variance, suggesting that there are indeed signaling interactions transferable among cell lines.

However, we found two major pitfalls in the predictions. First, the models captured much better the trends than the absolute value of nodes’ activities in cases where the basal marker intensity of the test cell lines was much higher than of the training cell lines. If the absolute values are important, this can be an important limitation, but not for downstream analysis where the data are rescaled, e.g. (Tognetti *et al*, 2021). In addition, the functional effect of at least some main pathways is driven by fold changes, and thus, predicting these, even if not being able to do so accurately for the absolute values, is helpful (Adler & Alon, 2018). The second pitfall is related to rare signaling cases: In the majority of cell lines, MEK phosphorylated ERK and the inhibition of MEK resulted in the lower phosphorylation of ERK. However, in a few cell lines, ERK showed a similar level of phosphorylation to EGF and Serum with and without the MEK inhibitor CI‐1040. This phenomenon was not captured by the predictions either because the number of such cell lines was low in the training data or because there was no information on this alternative relationship in the data, for example, if this phenomenon depends on the expression of non‐measured nodes. It might also be that those cell lines showed this behavior because the high concentration of MEK inhibitor resulted in cell type‐specific off‐target effects. This example illustrates the potential limitations of inhibitors for network inference, as off‐target effects can confound the results.

The activity of cellular markers within a single condition in a cell line could show twofold to threefold differences across single cells. The fact that models can explain up to 80% of this variance by the other measured markers shows that despite the loose regulation between the cells, the intracellular signaling is highly ordered and the activity of the proteins is strongly connected. This phenomenon is already recognized and is used by network inference algorithms, e.g. (Krishnaswamy *et al*, 2014). Furthermore, the models were trained on some cell lines and predicted the behavior in other cell lines, which show that the learnt regulations are transferable between cell lines. However, our finding that certain rare signaling patterns were not captured well shows that the transferred knowledge is limited and prediction algorithms should improve to capture rare cases.

We tested the winner’s method on an independent mass cytometry dataset (Lun *et al*, 2017), where we found that the predictions are much better than random and the prediction error is smaller than what we observed in Subchallenge 1. This is likely because the “pseudo‐cell lines” (cells that overexpress different proteins) are more similar to each other than the breast cancer cell lines. In any case, this result suggests that the method is applicable on other datasets.

The approach to predict the whole signaling response of the cells to kinase inhibitors (Subchallenges 2 and 3) was fundamentally different from Subchallenge 1. The top‐performing models learnt the signaling relationships among the nodes and how these changed between conditions within each cell line, and transferred this knowledge to the target condition. Further, instead of predicting each individual single cell directly, the top‐performing models derived statistical descriptors of the markers in each condition, predicted the distribution of cells in the target condition, and then sampled that distribution to generate the single cell predictions. We found that the predictions of the top team were almost as accurate as biological replicas, although it should be noted that we only had two replicates. Although models that inferred directly the median and variance of cells performed better than models based on resampling similar cell lines, the analysis of the distribution across cells showed that the latter approach performed better at capturing the cell lines’ heterogeneity.

In Subchallenge 4, the task was even more challenging: predict the time‐course response of cell lines for which only basal (before perturbation) data were provided. Many methods performed better than using an average of the provided cell lines as a predictor, showing that participants were able to capture to some degree the differences between the cell lines, particularly for phosphoproteins that did not change much across time, but varied between cell lines.

The teams used various prediction algorithms, including linear models (ordinary least squares, ridge regression, regularized linear regression), (recurrent‐) neural networks, tree‐based algorithms (e.g., random forest, gradient boosting), deep networks, and custom differential equation‐based models (see Appendix: Prediction methods of the best teams). However, similarly to previous we did not find evidence for the idea that a specific type of algorithm performed significantly better than other types of models for these prediction challenges. Across subchallenges, we found that the ensemble of predictions of multiple teams is better than an average team, which means that the risk for a bad prediction can be mitigated by combining multiple approaches. However, the combined prediction is not significantly better than the best team, which might be because the best team already used an ensemble of models.

Only some teams used the prior knowledge we provided, and none added any further external prior knowledge. Teams that did use this knowledge did not perform better, in contrast to the previous network inference challenge (Hill *et al*, 2016). In fact, top‐performing teams did not use prior knowledge, except the best performing team that used the prior network to predict the effect of mTOR inhibition on other nodes in Subchallenge 3. While the value of prior knowledge is considered helpful for network inference, it is not trivial to embed this in general machine learning frameworks, which could explain why it was not particularly relevant in this challenge.

The results of our challenge also provide general guidelines to design experiments aimed to characterize signaling events at the single‐cell level. Given the broadly good results from Subchallenge 1, one could design experiments where only subsets of markers are measured in the different conditions, as long as each phospho‐protein is measured in at least one condition for each cell line, even if some of these are bulk measurements. Further, in agreement with previous studies (Behbehani *et al*, 2012; Rapsomaniki *et al*, 2018), the data in subchallenges 2 and 3 show that some processes, like cell cycle (Fig 5C), strongly influence signaling. It is important to include markers that either capture these processes or are co‐regulated by them.

Further challenges could also address how to predict specifically the basal single‐cell signaling from other omics data. If achieved, combining this with improvements of the methods from subchallenge 4, one could predict the single‐cell dynamics of signaling networks directly from bulk omics data. In this challenge, we focused on predicting early signaling responses in breast cancer. Signaling responses have been used for predicting long‐term phenotypic changes, such as drug response. Bulk signaling response was used to successfully predict long‐term drug response (Niepel *et al*, 2013) and dynamic pathway models derived from signaling responses to perturbations predicted single and combined treatments in cancer cell lines (Korkut *et al*, 2015; Eduati *et al*, 2017; Silverbush *et al*, 2017; Tognetti *et al*, 2021). An interesting prospect for future challenges would thus be to predict long‐term phenotypes from cell signaling at the single‐cell level, which can provide important insights into the mechanisms of drug resistance.

In summary, the results of this DREAM challenge provide a snapshot of our collective ability to predict signal transduction at the single‐cell level. The best performing methods can be applied to other datasets and used as a baseline for those interested in developing methods for these aims. To support those further developments, all data and descriptions of methods are made freely available for the community to use.

## Materials and Methods

### Reagents and Tools table

**Table:** 

Resource	Reference or source	Identifier or catalog number
**Experimental data**		
Single‐cell data	Tognetti *et al* (2021)	NA
Proteomics data	Tognetti *et al* (2021)	NA
Genomics data	Marcotte *et al* (2016)	NA
External single‐cell data	Lun *et al* (2017)	NA
Predictions from the teams	https://www.synapse.org/#!Synapse:syn20366914/wiki/594733	NA
**Software**		
Code for analysis and figures	https://codeocean.com/capsule/7326564/	NA
Prediction methods of each team	https://www.synapse.org/#!Synapse:syn20366914/wiki/594733	NA

### Methods and Protocols

#### Single cell signaling in breast cancer DREAM challenge

The challenge was divided into three rounds which lasted between 2 and 4 weeks each. In each round, participants were limited to maximum three submissions, to avoid learning on the test dataset. After each round, the current scores were publicly presented on a scoring board. In total 127, 77, 61, and 83 valid predictions were submitted by 22, 16, 14, and 25 teams for each subchallenge, respectively.

A requirement for the challenge participation is a summary of method, which is publicly available (https://www.synapse.org/#!Synapse:syn20366914/wiki/593925) and open source code that reproduces the results. Further, we collected information on the methods via a questionnaire.

#### Experimental data used in the challenge

In the challenge, the participants were provided with 3 main datasets (summarized by Appendix Fig S2): (i) the single‐cell phosphoproteomic data and median intensities of the phosphoproteomics data, (ii) protein abundance data, and (iii) genomic data: RNA sequencing data, copy number variation (CNV), and single nucleotide polymorphism (SNP). The raw data and the experimental protocol for (i) and (ii) are described in Tognetti *et al* (2021) and for (iii) in Tognetti *et al* (2021); Marcotte *et al* (2016). In what follows, we summarize the experimental detail that is relevant to the challenge.

The single‐cell phosphoproteomic dataset comprises the measurement of 62 breast cancer and 5 normal cell lines in six treatment conditions. The treatment conditions included stimuli with EGF and serum after 24 h of starvation and stimuli with EGF and serum after 24 h of starvation and 15 min of incubation with one of the five kinase inhibitors. The kinase inhibitors and their concentrations are listed in Appendix Table S3. The adhesive cells were detached 5 min prior to fixation. The effect of detachment is expected to increase the baseline level of certain pathways, but this effect is constant across all experiments and is not expected to impact significantly time‐dependent effects. The response of the cells was measured at 7‐10 time points in the first hour after treatment (Appendix Fig S2A). In each condition, 36 markers were measured by mass cytometry, which included 31 phosphoproteins and 5 cellular markers.

The protein abundance data were measured at normal growth condition in basal state. Participants were provided by both the raw proteomic data, which contained biological and technical replicates, and the protein abundance output from MSstat (Choi *et al*, 2014), which provides the relative quantification (log2 fold changes) to the set of five cell lines that were derived from healthy patients (184A1, 184B5, MCF10A, MCF10F, and MCF12A).

The RNAseq, CNV, and SNP datasets were generated and published (Marcotte *et al*, 2016) and have information for 64 of the characterized 67 cell lines.

#### Scores and robust ranking by resampling

In subchallenge 1, we computed the root mean square error between the measured data y and prediction $\hat{y}$ for each marker m, separately in each condition, on single‐cell level. In a particular condition, there are $N_{cl,tr,t}$ single cells that are measured and predicted in each test cell line cl, treatment tr, and time point t after the perturbation. The RMSE is computed as

$$RMSE_{cl,tr,t,m}=\sqrt{\sum_{i=1}^{N_{cl,tr,t}}\frac{{\left(y_{i,cl,tr,t,m}‐{\hat{y}}_{i,cl,tr,t,m}\right)}^{2}}{N_{cl,tr,t}}}$$

For the final score of a team, we computed the average RMSE error across the five predicted markers (p‐ERK, p‐PLCg2, p‐HER2, p‐S6, and p‐AKT_S473), six cell lines (AU565, EFM19, HCC2218, LY2, MACLS2, and MDAMB436), and all measured timepoints (11 in stimulated experiments, 7 in case of combined stimulus and inhibition):

$$score=\frac{1}{N_{\mathrm{cond}}}\sum_{cl,tr,t,m}RMSE_{cl,tr,t,m}$$

We found that the RMSE score changes in each condition, most significantly based on the cell line that was predicted. Therefore, for a robust ranking of participants, we resampled 1000 times the conditions used to compute the final score with replacement and computed the bootstrap score on each sample. Finally, the team are compared by Bayes factors based on the bootstrap scores:

$$BF\left(T_{x,}T_{y}\right)=\frac{\sum_{i=1}^{1000}1\left(score{\left(T_{x}\right)}_{i}<score{\left(T_{y}\right)}_{i}\right)}{\sum_{i=1}^{1000}1\left(score{\left(T_{x}\right)}_{i}>score{\left(T_{y}\right)}_{i}\right)}$$

where $score\left(T_{x}\right)$ is the score of team $T_{x}$, 1() is the indicator function.

In subchallenges 2 and 3, the participants submitted 10`000 representative single‐cell predictions (predicting $N_{\mathrm{marker}}=35$ markers) for each validation cell line and condition (treatment and time). For each condition i, we compared the mean marker value from the gold standard data $\left(\mu \right)$ with the mean of the predicted sample $\left(\hat{\mu }\right)$.

$$S_{\mu ,i}=\sum_{j=1}^{N_{mar\ker}}{\left(\mu_{i,j}‐{\hat{\mu }}_{i,j}\right)}^{2},$$

and also for the covariance of marker pairs:

$$S_{\sigma ,i}=\sum_{j\leq k}^{N_{mar\ker}}{\left(\text{cov}\left(y_{i,j},y_{i,k}\right)‐\text{cov}\left({\hat{y}}_{i,j},{\hat{y}}_{i,k}\right)\right)}^{2}$$

and combined the two parts equally when computed the average across all cell lines and conditions (*N*):

$$S=\frac{1}{N}\sum_{i=1}^{N}\left(S_{\mu ,i}+S_{\sigma ,i}\right).$$

For a robust ranking, we created bootstrap samples of scores by resampling the conditions and cell lines with replacement. Then, we computed the Bayes factors between teams, based on the bootstrap scores.

In subchallenge 4, the participants predict the population mean response to perturbation over time for each marker. Therefore, for each cell line and treatment, we computed the RMSE across time:

$$RMSE_{cl,tr,m}=\sqrt{\sum_{t=1}^{T}\frac{{\left(y_{,t,m}‐{\hat{y}}_{t,m}\right)}^{2}}{T}}$$

The final score is computed by averaging the error for all conditions.

For a robust ranking, we created bootstrap samples of scores by resampling the treatments and cell lines with replacement, as before. Then, we computed the Bayes factors between teams.

#### Reference models

In SC1, our model predicted the missing marker based on the measured markers in each individual cell. We built a random forest model for each marker independently, across all conditions using the *ranger* R package (Wright & Ziegler, 2017). The model features included all the measured markers and time as continuous variables, and the treatments, starvation status (cells in treatment “full” were not starved), and stimulated status (cells at time 0 and in “full” treatment are not stimulated with EGF) as one‐hot‐encoded variables. The model was trained on a subset of the training cell lines: We selected 7 cell lines randomly (*BT474*, *CAL148*, *HBL100*, *MCF7*, *MDAMB157*, *TD47D*, and *ZR7530*) and 500 random cells from each condition.

The idea for the reference model in SC2 was to predict the means and covariance matrix of the markers in the missing conditions based on the available conditions and then use a simulator that generates samples from a multivariate normal distribution following the estimated mean and covariance values. Thus, first we computed the mean and covariance matrix of the markers in each cell line, treatment, and time point. Then, we built an independent random forest model for each statistical variable *y* (mean of a marker or entry in the covariance matrix), for each cell line and time point:

$$\hat{y_{tr_{i}}}=RF\left(y_{tr_{j\neq i}}\right)$$

For example, the mean value of marker *Ki‐67*, in cell line *BT474*, time 0, treatment iMEK was predicted based on the available values of *Ki‐67* levels in the same cell line, in time 0, in {EGF, iEGFR, iPI3K, iPKC} conditions. For the training of the models, we selected the same 7 training cell lines as in SC1.

We have not built a reference model for subchallenge 3.

In subchallenge 4, we hypothesized that each marker response ($y_{cl,tr,time}$) can be described as the superposition of the average cell line response ($y_{average,tr,time}=\frac{1}{N_{\mathrm{cl}}}\sum_{\mathrm{cl}}y_{tr,time}$) and cell line‐specific response difference ($d_{cl,tr,time}$), i.e.,

(1) $$y_{cl,tr,time}=y_{average,tr,time}+d_{cl,tr,time}.$$

The average cell line response ($y_{average,tr,time}$) was calculated by taking the mean of all the training cell lines for each marker, in each condition (treatment and time point). Note that all the three terms in Equation (1) can be easily calculated for the training data. Then, we built a random forest model that predicts the cell line‐specific response ($d_{cl,tr,time}$) based on the data that is available for the test conditions, i.e., based on the proteomic data and based on the basal activation of markers (“full” condition). We also used the treatment, time, and stimulation status (i.e., if time > 0) as model features. We trained a model for each reporter independently based on all the training cell lines.

#### Random and systematic combinations of predictions

Predictions of randomly selected teams and the top‐performing teams were aggregated. Different sizes of ensembles (*n*) were scored, from the minimum of one team up to the maximum of all teams. In subchallenges 1 and 4, the predicted values of different teams were combined by taking the median of the predictions. In subchallenges 2 and 3, representative cells were predicted and scored based on the statistics. Here, we used two approaches: (i) Predictions were combined by sampling an equal number of predicted values per condition from all selected submissions and (ii) computing the statistics for each submission and then averaging across the selected teams. In all subchallenges, the resulting ensembles were scored the same way as individual submissions. For the random combination of teams, we repeated the procedure 100 times to see the distribution of score.

#### Machine learning‐based combination of predictions

We used Random Forest (RF) models in SC1 and SC2 to combine the predictions for the test cell lines. We used a cross‐validation scheme for the model training and for the predictions to avoid overfitting these RF models and to generate predictions for all the test cell lines. We divided the data into sixfold: In SC1, each fold of data contained a single test cell line, and in SC2, each fold contained two cell lines. This way the training and predictions of the RF models also mimic the setup of the subchallenges; i.e., they are trained on part of the cell lines and predict other cell lines. Then in six iterations, we trained our RF models on fivefold and predicted the sixth fold until we had predictions for all the test data and could compare the performance of RF predictions with the participants’ predictions.

In SC1, we trained a random forest model per marker and used the single‐cell predictions from the teams and the time point as continuous variables and the treatment and predicted marker as one‐hot encoded features. Due to the chosen cross‐validation scheme, we could not use “cell line” as a feature. Therefore, we also included the 32 non‐predicted marker values as model features.

In SC2, our RF predicted the mean and covariances per condition using the mean and covariance matrix from the participants’ predictions. The model also included as features the median expression per marker at treatment EGF. The predicted statistics were used to create a multivariate normal distribution, from which 10,000 cells per condition were sampled and subsequently scored.

#### Cell cycle phases

Cell cycle phases were identified from the single‐cell data based on the markers *Histone H3* (p.H3), *iododeoxyuridine* (IdU), *retinoblastoma protein* (p.RB), and *cyclinB* (Appendix Fig S8). Following *(*Behbehani *et al*, 2012 *)*, G0‐phase and G1‐phase are both characterized by low expression of IdU, p.H3, and cyclinB, but we could not differentiate between G0 and G1 based on the expression of *retinoblastoma protein* (p.RB). High expression of IdU and low expression of p.H3 characterize S‐phase; high expression of p.H3 with low IdU identifies M‐phase; finally, low expression of IdU and p.H3 with high expression of cyclinB identifies G2 phase. Apoptotic cells were detected by high cleaved *Caspase 3* (cleavedCas) levels.

## Author contributions

AG, MT, JS‐R, and BB conceived and designed the Single Cell Signaling in Breast Cancer DREAM challenge with the help of TY and VC. AG and AD analyzed the challenge results with the help of MT and JT, under the supervision of JS‐R. The top‐performing approaches were designed by BG, WC, and HS. The DREAM Consortium provided predictions, method implementations, and descriptions. AG and JS‐R drafted the manuscript with inputs from MT, AD, and JT. All authors read, commented, and approved the final manuscript.

## Conflict of interest

JSR has received funding from GSK and Sanofi and expects consultant fees from Travere Therapeutics. MT is an employee of Biognosys AG (Zurich, Switzerland).

## Supporting information



Appendix

## Data Availability

The datasets and computer code produced in this study are available in the following databases:
Single‐cell proteomic, bulk proteomic, transcriptomic, and genomic data: https://www.synapse.org/#!Synapse:syn20564743
Description of prediction methods and code of the challenge participants: https://www.synapse.org/#!Synapse:syn20366914/wiki/594733
Prediction data and analysis scripts: CodeOcean capsule. Single Cell Signaling in Breast Cancer DREAM challenge analysis: https://doi.org/10.24433/CO.6078101.v2.

## References

[msb202110402-bib-0001] Adler M , Alon U (2018) Fold‐change detection in biological systems. Curr Opin Syst Biol 8: 81–89

[msb202110402-bib-0002] Behbehani GK , Bendall SC , Clutter MR , Fantl WJ , Nolan GP (2012) Single‐cell mass cytometry adapted to measurements of the cell cycle. Cytometry A 81: 552–566 22693166 10.1002/cyto.a.22075 PMC3667754

[msb202110402-bib-0003] Benveniste EN , Liu Y , McFarland BC , Qin H (2014) Involvement of the janus kinase/signal transducer and activator of transcription signaling pathway in multiple sclerosis and the animal model of experimental autoimmune encephalomyelitis. J Interferon Cytokine Res 34: 577–588 25084174 10.1089/jir.2014.0012 PMC4118716

[msb202110402-bib-0004] Bodenmiller B , Zunder ER , Finck R , Chen TJ , Savig ES , Bruggner RV , Simonds EF , Bendall SC , Sachs K , Krutzik PO *et al* (2012) Multiplexed mass cytometry profiling of cellular states perturbed by small‐molecule regulators. Nat Biotechnol 30: 858–867. 22902532 10.1038/nbt.2317 PMC3627543

[msb202110402-bib-0005] Boucher J , Kleinridders A , Kahn CR (2014) Insulin receptor signaling in normal and insulin‐resistant states. Cold Spring Harb Perspect Biol 6: a009191 24384568 10.1101/cshperspect.a009191 PMC3941218

[msb202110402-bib-0006] Byrne K , Monsefi N , Dawson J , Degasperi A , Bukowski‐Wills J‐C , Volinsky N , Dobrzyński M , Birtwistle M , Tsyganov M , Kiyatkin A *et al* (2016) Bistability in the Rac1, PAK, and RhoA signaling network drives actin cytoskeleton dynamics and cell motility switches. Cell Syst 2: 38–48 27136688 10.1016/j.cels.2016.01.003 PMC4802415

[msb202110402-bib-0007] Choi M , Chang C‐Y , Clough T , Broudy D , Killeen T , MacLean B , Vitek O (2014) MSstats: an R package for statistical analysis of quantitative mass spectrometry‐based proteomic experiments. Bioinformatics 30: 2524–2526. 24794931 10.1093/bioinformatics/btu305

[msb202110402-bib-0008] Eduati F , Doldàn‐Martelli V , Klinger B , Cokelaer T , Sieber A , Kogera F , Dorel M , Garnett MJ , Blüthgen N , Saez‐Rodriguez J (2017) Drug resistance mechanisms in colorectal cancer dissected with cell type‐specific dynamic logic models. Can Res 77: 3364–3375. 10.1158/0008-5472.CAN-17-0078 PMC6433282 28381545

[msb202110402-bib-0009] Hill SM , Heiser LM , Cokelaer T , Unger M , Nesser NK , Carlin DE , Zhang Y , Sokolov A , Paull EO , Wong CK *et al* (2016) Inferring causal molecular networks: empirical assessment through a community‐based effort. Nat Methods 13: 310–318 26901648 10.1038/nmeth.3773 PMC4854847

[msb202110402-bib-0010] Hynes NE , MacDonald G (2009) ErbB receptors and signaling pathways in cancer. Curr Opin Cell Biol 21: 177–184 19208461 10.1016/j.ceb.2008.12.010

[msb202110402-bib-0011] Korkut A , Wang W , Demir E , Aksoy BA , Jing X , Molinelli EJ , Babur Ö , Bemis DL , Onur Sumer S , Solit DB *et al* (2015) Perturbation biology nominates upstream–downstream drug combinations in RAF inhibitor resistant melanoma cells. Elife 4: e04640 10.7554/eLife.04640 PMC4539601 26284497

[msb202110402-bib-0012] Krishnaswamy S , Spitzer MH , Mingueneau M , Bendall SC , Litvin O , Stone E , Pe’er D , Nolan GP (2014) Systems biology. Conditional density‐based analysis of T cell signaling in single‐cell data. Science 346: 1250689 25342659 10.1126/science.1250689 PMC4334155

[msb202110402-bib-0013] Loos C , Moeller K , Fröhlich F , Hucho T , Hasenauer J (2018) A hierarchical, data‐driven approach to modeling single‐cell populations predicts latent causes of cell‐to‐cell variability. Cell Syst 6: 593–603 29730254 10.1016/j.cels.2018.04.008

[msb202110402-bib-0014] Lun X‐K , Zanotelli VRT , Wade JD , Schapiro D , Tognetti M , Dobberstein N , Bodenmiller B (2017) Influence of node abundance on signaling network state and dynamics analyzed by mass cytometry. Nat Biotechnol 35: 164–172 28092656 10.1038/nbt.3770 PMC5617104

[msb202110402-bib-0015] Marbach D , Costello JC , Küffner R , Vega NM , Prill RJ , Camacho DM , Allison KR , Kellis M , Collins JJ , Stolovitzky G (2012) Wisdom of crowds for robust gene network inference. Nat Methods 9(8): 796–804. 22796662 10.1038/nmeth.2016 PMC3512113

[msb202110402-bib-0016] Marcotte R , Sayad A , Brown K , Sanchez‐Garcia F , Reimand J , Haider M , Virtanen C , Bradner J , Bader G , Mills G *et al* (2016) Functional genomic landscape of human breast cancer drivers, vulnerabilities, and resistance. Cell 164: 293–309 26771497 10.1016/j.cell.2015.11.062 PMC4724865

[msb202110402-bib-0017] Meyer P , Saez‐Rodriguez J (2021) Advances in systems biology modeling: 10 years of crowdsourcing DREAM challenges. Cell Syst 12: 636–653 34139170 10.1016/j.cels.2021.05.015

[msb202110402-bib-0018] Niepel M , Hafner M , Pace EA , Chung M , Chai DH , Zhou L , Schoeberl B , Sorger PK (2013) Profiles of Basal and stimulated receptor signaling networks predict drug response in breast cancer lines. Sci Signal 6: ra84 24065145 10.1126/scisignal.2004379 PMC3845839

[msb202110402-bib-0019] Prill RJ , Saez‐Rodriguez J , Alexopoulos LG , Sorger PK , Stolovitzky G (2011) Crowdsourcing network inference: the DREAM predictive signaling network challenge. Sci Signal 4: mr7 21900204 10.1126/scisignal.2002212 PMC3465072

[msb202110402-bib-0020] Rapsomaniki MA , Lun X‐K , Woerner S , Laumanns M , Bodenmiller B , Martínez MR (2018) Cell CycleTRACER accounts for cell cycle and volume in mass cytometry data. Nat Commun 9: 632 29434325 10.1038/s41467-018-03005-5 PMC5809393

[msb202110402-bib-0021] Rukhlenko OS , Khorsand F , Krstic A , Rozanc J , Alexopoulos LG , Rauch N , Erickson KE , Hlavacek WS , Posner RG , Gómez‐Coca S *et al* (2018) Dissecting RAF Inhibitor Resistance by Structure‐based Modeling Reveals Ways to Overcome Oncogenic RAS Signaling. Cell Syst 7: 161–179 30007540 10.1016/j.cels.2018.06.002 PMC6149545

[msb202110402-bib-0022] Saez‐Rodriguez J , Costello JC , Friend SH , Kellen MR , Mangravite L , Meyer P , Norman T , Stolovitzky G (2016) Crowdsourcing biomedical research: leveraging communities as innovation engines. Nat Rev Genet 17: 470–486 27418159 10.1038/nrg.2016.69 PMC5918684

[msb202110402-bib-0023] Silverbush D , Grosskurth S , Wang D , Powell F , Gottgens B , Dry J , Fisher J (2017) Cell‐specific computational modeling of the PIM pathway in acute myeloid leukemia. Cancer Res 77: 827–838 27965317 10.1158/0008-5472.CAN-16-1578

[msb202110402-bib-0024] Spitzer MH , Nolan GP (2016) Mass cytometry: single cells, many features. Cell 165: 780–791 27153492 10.1016/j.cell.2016.04.019 PMC4860251

[msb202110402-bib-0025] Tognetti M , Gabor A , Yang M , Cappelletti V , Windhager J , Rueda OM , Charmpi K , Esmaeilishirazifard E , Bruna A , de Souza N *et al* (2021) Deciphering the signaling network of breast cancer improves drug sensitivity prediction. Cell Syst 12: 401–418.e12 33932331 10.1016/j.cels.2021.04.002

[msb202110402-bib-0026] Wright MN , Ziegler A (2017) ranger: a fast implementation of random forests for high dimensional data in C and R. J Stat Softw 77: 1–17

[msb202110402-bib-0028] Yaffe MB (2019) Why geneticists stole cancer research even though cancer is primarily a signaling disease. Sci Signal 12: eaaw3483 30670634 10.1126/scisignal.aaw3483

[msb202110402-bib-0027] Zoppoli P , Morganella S , Ceccarelli M (2010) TimeDelay‐ARACNE: reverse engineering of gene networks from time‐course data by an information theoretic approach. BMC Bioinformatics 11: 154 20338053 10.1186/1471-2105-11-154 PMC2862045

