# Supplementary material for: Cell‐to‐cell and type‐to‐type heterogeneity of signaling networks: insights from the crowd
Source: Mol Syst Biol. 2021 Oct 18;17(10):e10402. doi: 10.15252/msb.202110402 (PMC8522707; doi:10.15252/msb.202110402)
Supplement: Supplementary file 1 — Appendix [file MSB-17-e10402-s002.pdf]

Appendix: Cell-to-cell and type-to-type heterogeneity of signaling networks: Insights from the crowd

Attila Gabor[1,#], Marco Tognetti[2,#], Alice Driessen[1], Jovan Tanevski[1], Baosen Guo[3], Wencai Cao[3], He Shen[3], Thomas Yu[4], Verena Chung[4], Single Cell Signaling in Breast Cancer DREAM Consortium members, Bernd Bodenmiller[2,*], Julio Saez-Rodriguez[1,*]

[1] Heidelberg University, Faculty of Medicine, and Heidelberg University Hospital, Institute for Computational Biomedicine, Bioquant, Heidelberg, Germany

[2] Department of Quantitative Biomedicine & Institute of Molecular Life Sciences, University of Zurich, Zurich, Switzerland

[3] Division of AI & Bioinformatics, Shenzhen Digital Life Institute, Shenzhen, China

[4] Sage Bionetworks, Seattle, USA

[#] These authors contributed equally to the work

* Corresponding authors: pub.saez@uni-heidelberg.de, bernd.bodenmiller@uzh.ch

Table of Content:

Appendix: Cell-to-cell and type-to-type heterogeneity of signaling networks: Insights from the crowd 1

Appendix Figure S1: Measured phospho-proteins (in blue) and perturbation targets (indicated by drug symbols) in the single cell experiments. 3

Appendix Figure S2. Overview of data availability in the challenge. 4

Appendix Figure S3. Performance of teams, combined predictions and improved prediction in subchallenge 1. 5

Appendix Figure S4. Mean measured data (thick line with dots) and predictions (thin, semi-transparent lines) in SC1. 6

Appendix Figure S4(cont.) Mean measured data (thick line with dots) and predictions (thin, semi-transparent lines) in SC1 for the remaining markers. 8

Appendix Figure S5. Distribution of the prediction error score. 8

Appendix Figure S6. Combination of predictions for SC2. 9

Appendix Figure S7. UMAP projection of the data and predictions for the cell line BT483, iEGFR condition in subchallenge 2. 9

Appendix Figure S8 Single cell distributions colored by cell cycle marker expression 10

Error distributions 10

Appendix Table S1: ANOVA of RMSE in subchallenge 1. 10

Appendix Table S2: comparison of RMSE within group versus global distribution via t-test. subchallenge 1 12

Appendix Table S3: kinase inhibitors applied to single cell perturbation experiments. 13

Prediction methods of the best teams 13
Subchallenge 1 13
Method of team icx_bxai (rank: 1) 13
Method of team NAD (rank: 2) 14
Method of team SingleCellLand (rank: 3) 14
Subchallenge 2 15
Method of team icx_bxai (rank: 1) 15
Method of pqiu (rank 2) 16
Methods of team Orangeballs (rank 3) 17
Subchallenge 3 18
Method of team icx_bxai (rank 1) 18
Method of team Orangeballs (rank 2) 18
Method of team AMberland (rank 3) 19
Subchallenge 4 20
Method of team icx_bxai (rank: 1) 20
Method of team AMberland (rank 2) 20
Method of team orangeballs (rank 3) 21
Appendix Table S4: comparison of prediction accuracy to reference predictions and random predictions in SC1. 22
Appendix Table S5: comparison of prediction accuracy to reference predictions and EGF condition in SC2. 23
Appendix Table S6: comparison of prediction accuracy to reference predictions and average cell line in SC4. 25
Single cell prediction on an independent dataset 25
The new dataset 25
Appendix Figure S9. Overexpression dependent signaling. 26
The prediction task 26
Appendix Table S7. Overexpression groups assigned to training and test sets. 26
Prediction method 27
Evaluation of the predictions 27
Appendix Figure S10. Comparison of team's prediction with random predictions on an independent dataset. 27
DREAM consortium 28

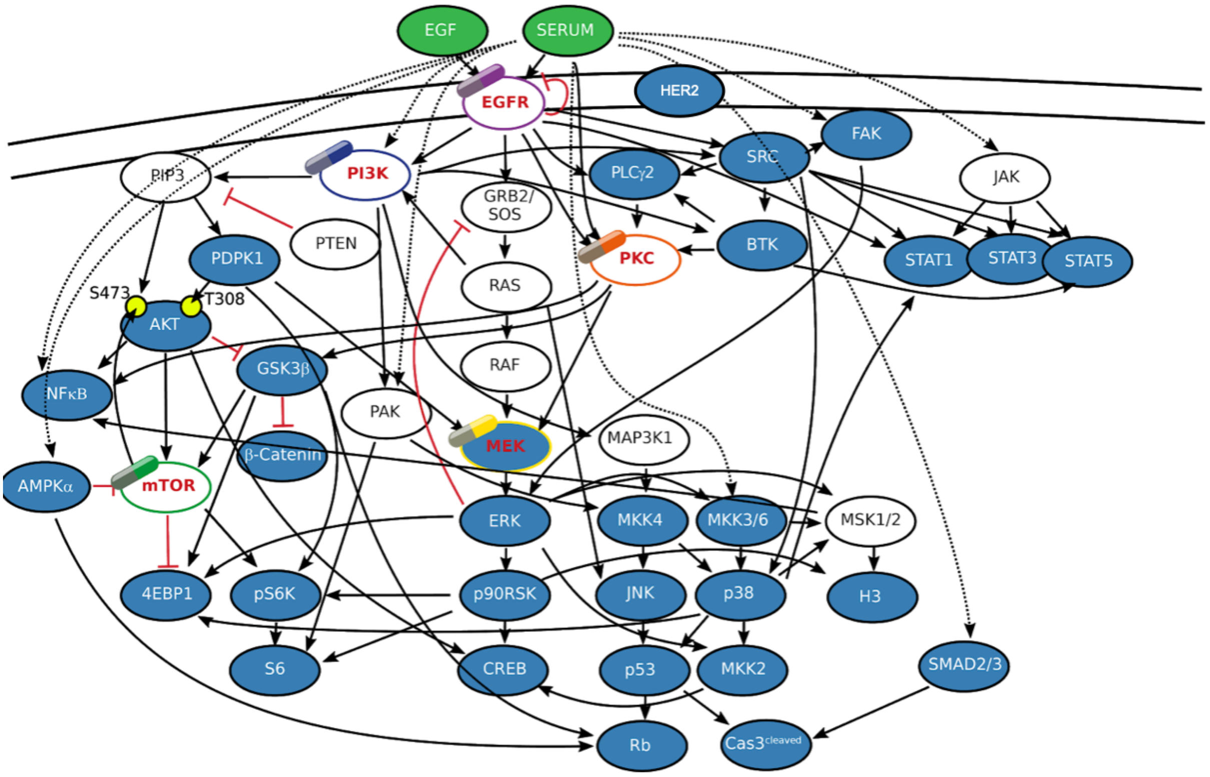

Appendix Figure S1: Measured phospho-proteins (in blue) and perturbation targets (indicated by drug symbols) in the single cell experiments.

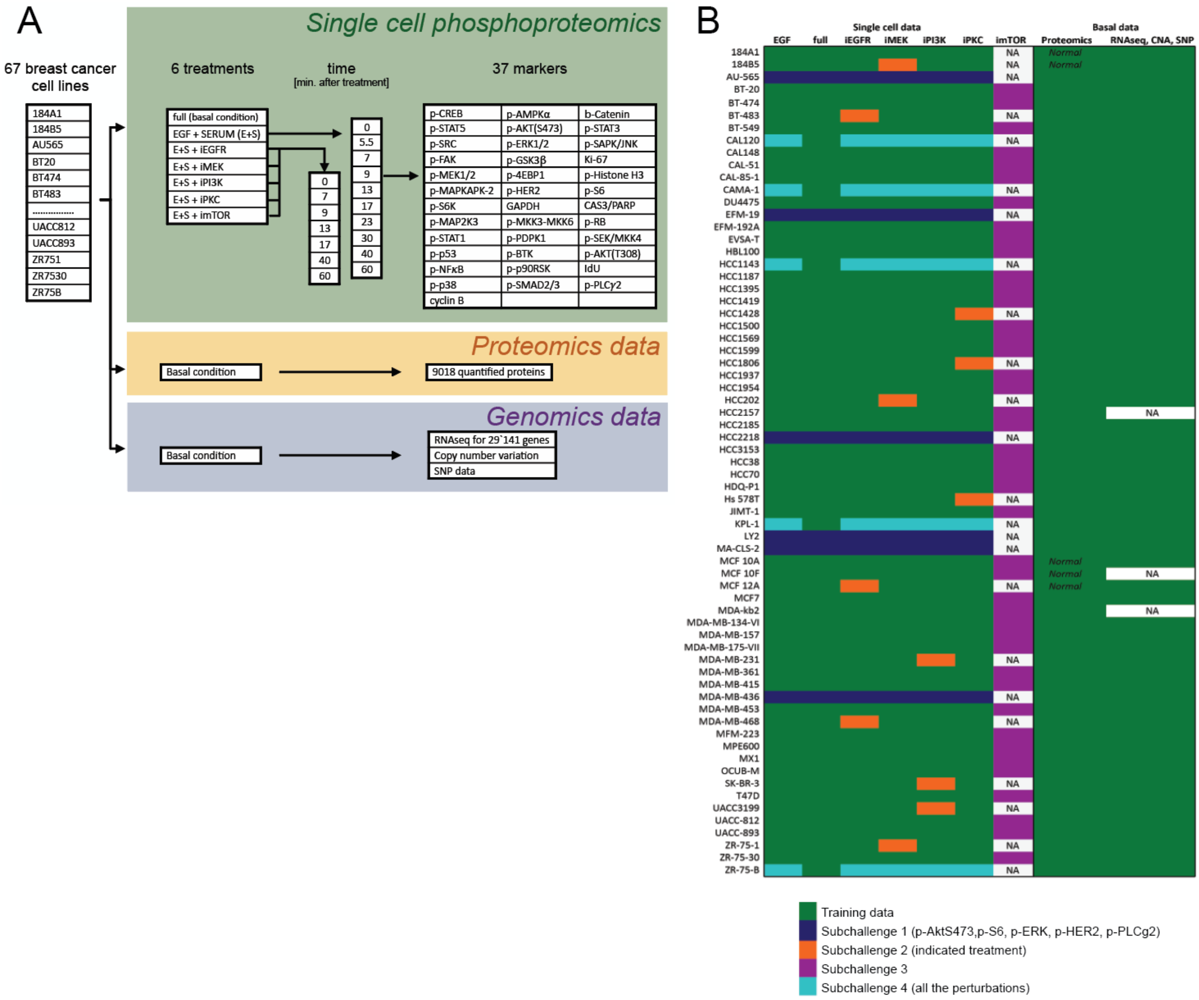

Appendix Figure S2. Overview of data availability in the challenge.
Panel A shows the three different types of data provided for the participants and the experimental conditions available for the single cell phosphorylation data. Panel B shows the distribution of the cell line data between training data (available for participants) and test data (used for scoring). The cells were starved before stimulation with EGF and incubated with the inhibitors for 15 minutes. The initial activation refers to the phosphorylation level at time 0, right before adding the EGF stimulus.This is different from basal activation, which represents the activation of cells growing in normal condition.

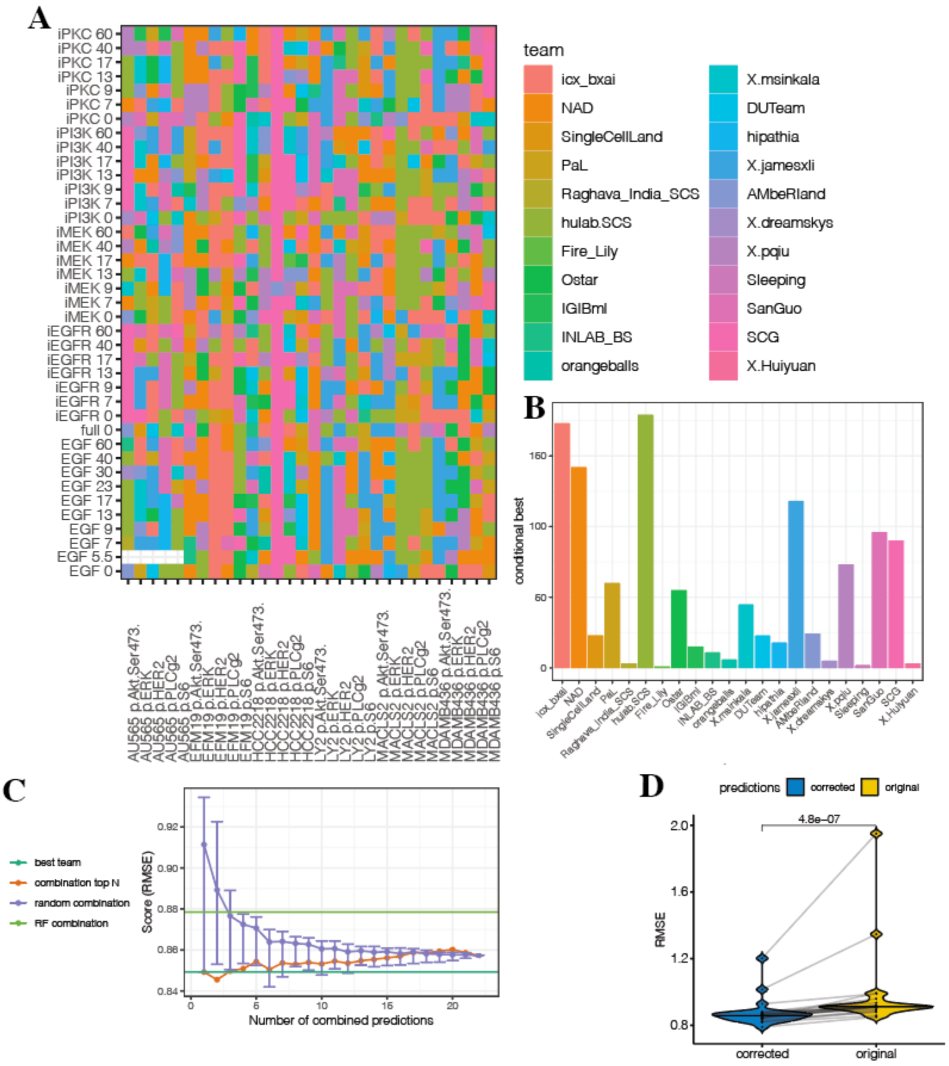

Appendix Figure S3. Performance of teams, combined predictions and improved prediction in subchallenge 1.

A) the tile plot shows the best performing team for each cell line, condition, treatment and time. B) number of conditions where a team performed the best. C) The performance of combined predictions depending on the number of considered teams (see Methods for details) D) improvement of teams' score after correcting their predictions by the difference between measured and predicted basal phosphorylation in each cell line.

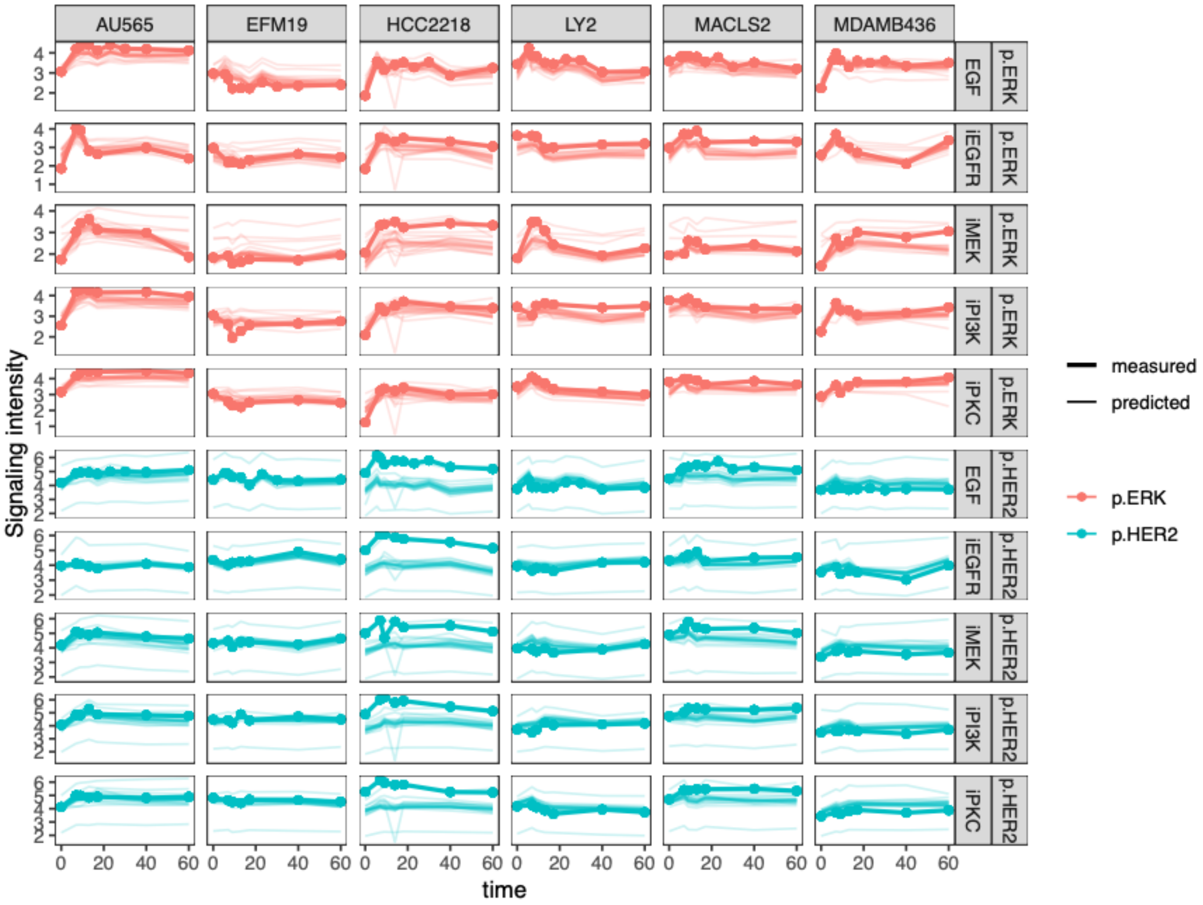

Appendix Figure S4. Mean measured data (thick line with dots) and predictions (thin, semi-transparent lines) in SC1.

Cell lines respond differently to the same perturbation (in rows). The effect of EGF stimuli in combination with the kinase inhibitors result in different responses. The median predictions of the teams are mostly well overlapping with the measurements. In case of larger differences (e.g. HCC2218, p.HER2, all treatments) the difference between prediction and measurements are almost constant in time.

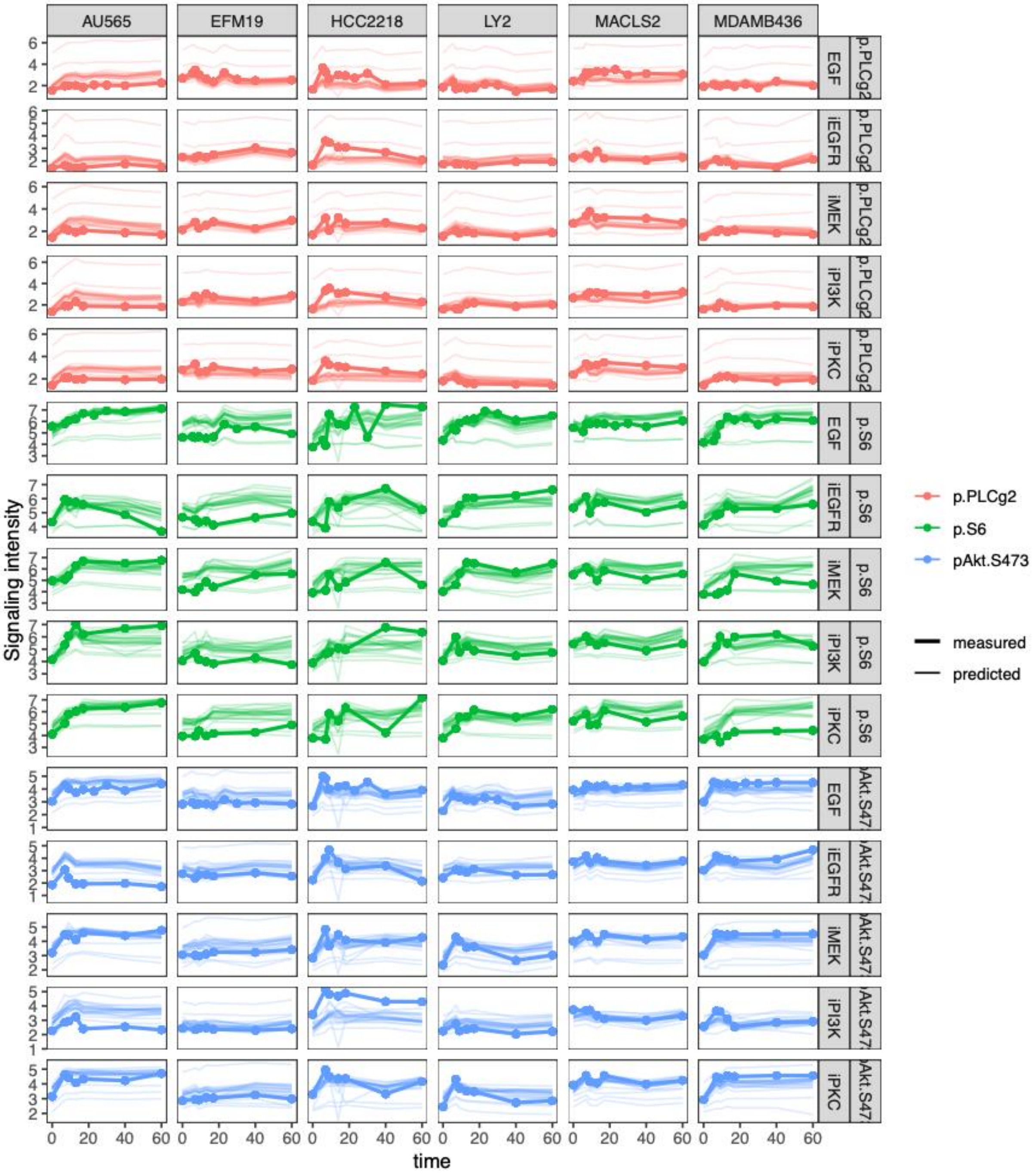
AU565
EFM19
HCC2218
LY2
MACLS2
MDAMB436
EGF
iEGFR
iMEK
iPI3K
iPKC
p.PLCg2
p.S6
pAkt.S473
Signaling intensity
time
measured
predicted

Appendix Figure S4(cont.) Mean measured data (thick line with dots) and predictions (thin, semi-transparent lines) in SC1 for the remaining markers.

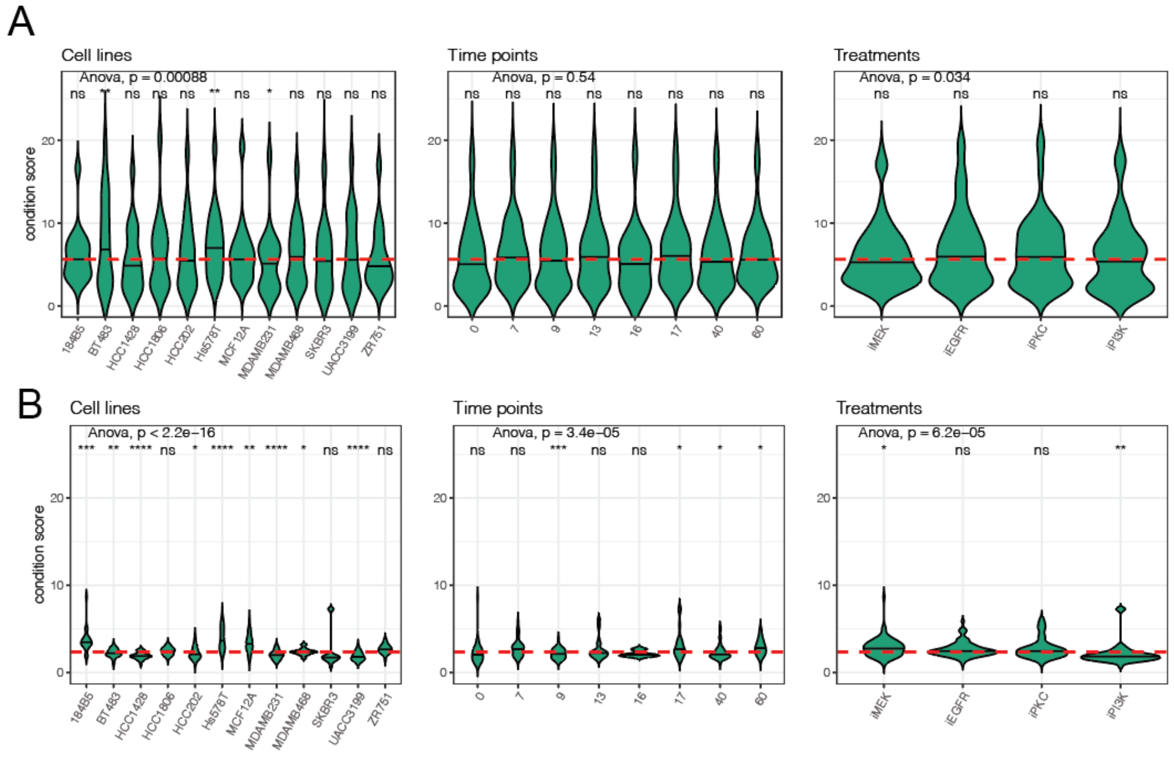

Appendix Figure S5. Distribution of the prediction error score.

*distribution of the prediction error score (squared difference of predicted and real median and covariances) across cell lines, treatments and time points in SC2. Each violin contains the predictions of A) all the teams, B) top 4 teams in each condition. The significance of a t-test between the individual groups and the global RMSE is shown above each violin plot (*: p<0.05, **: p<0.01, ***: p<0.001, ****: p<0.0001, ns: not significant)*

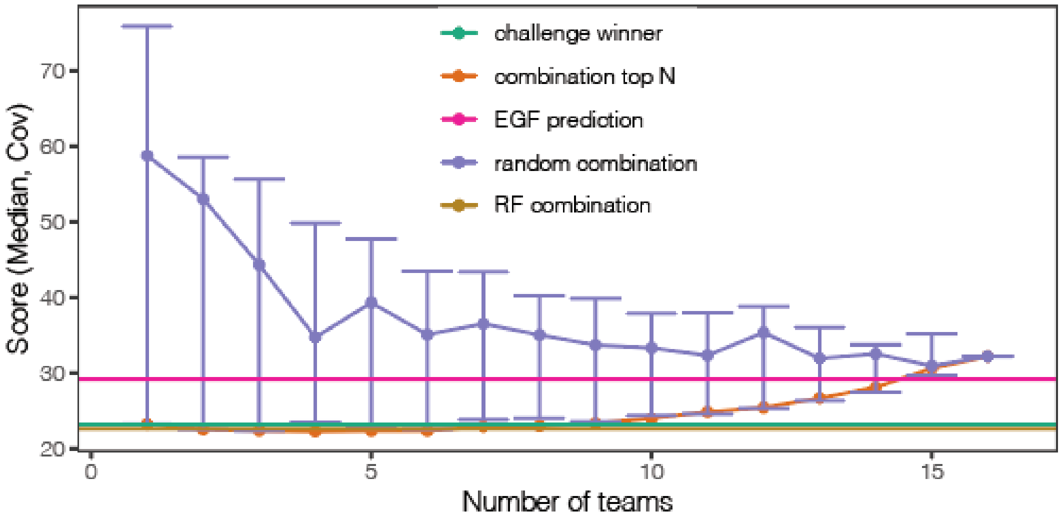

Appendix Figure S6. Combination of predictions for SC2.
Score of combined predictions based on the number of teams considered.

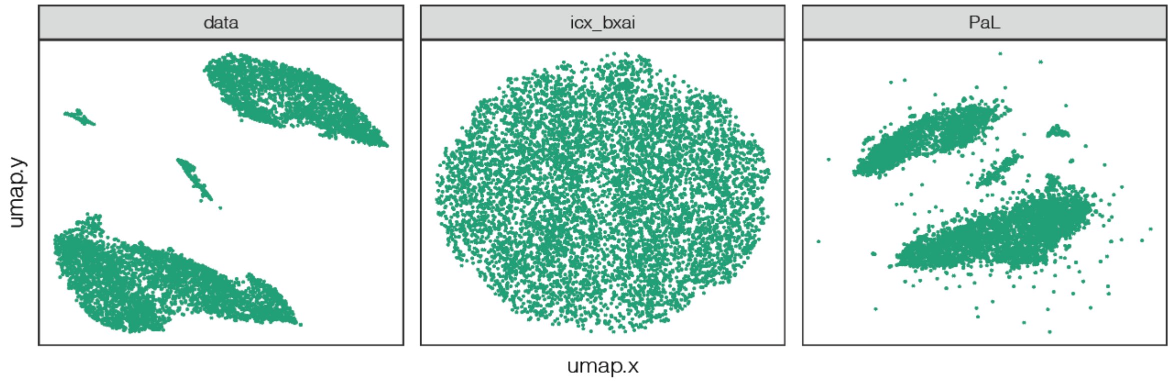

Appendix Figure S7. ***UMAP projection of the data and predictions for the cell line BT483, iEGFR condition in subchallenge 2.***
The projection of data and predictions independently to a 2D manifold reveals clusters for the data and for the predictions from PaL (used a resampling method), but shows a uniform distribution for the challenge winner's prediction (icx_bxai, used a Gaussian sampler)

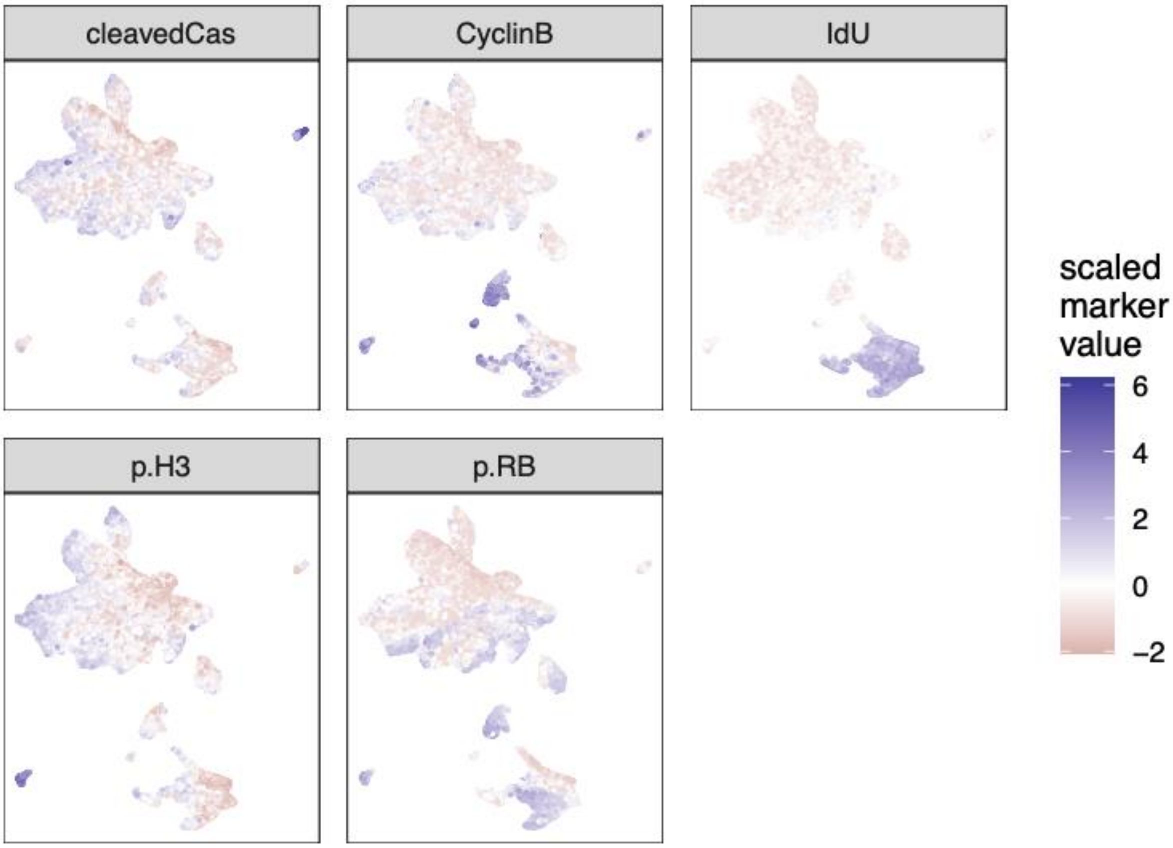

Appendix Figure S8 Single cell distributions colored by cell cycle marker expression
SC2 test data shown on two-dimensional Uniform Manifold Approximation and Projection (UMAP) colored by markers used to identify cell-cycle phases (see Methods)

Error distributions

We tested in each subchallenges, how the RMSE distributes across cell lines, treatment, marker and treatments. We evaluated this via an ANOVA test, where the null hypothesis is that the RMSE has the same mean value across the variables (Supp. Table 1). Then we compared the RMSE distribution within each group to the global distribution of RMSE (Supp. Table 2).

	Degree of freedom	Sum of Squares	Mean Squares	F-value	Significance	
cell_line	5	14.767	2.953	179.58	<2e-16	***
treatment	5	0.191	0.038	2.321	0.0413	*
time	9	0.269	0.03	1.817	0.0611	.
marker	4	31.335	7.834	476.342	<2e-16	***
Residuals	1141	18.765	0.016			

Appendix Table S1: ANOVA of RMSE in subchallenge 1.

SC1: marker vs global									
	marker	deltaRMSE	groupMeanRMSE	globalMeanRMSE	statistic	p.value	parameter	conf.low	conf.high
1	p.Akt.Ser473.	-0.0241	0.689	0.713	-2.01	4.52E-02	498	-0.0477	-0.000516
2	p.ERK	-0.144	0.569	0.713	-15.5	3.67E-48	903	-0.162	-0.126
3	p.HER2	-0.142	0.571	0.713	-10.6	2.48E-23	420	-0.169	-0.116
4	p.PLCg2	0.00543	0.718	0.713	0.517	6.05E-01	654	-0.0152	0.026
5	p.S6	0.305	1.02	0.713	16.3	9.17E-44	311	0.268	0.342
SC1: cell line vs global									
	cell_line	deltaRMSE	groupMeanRMSE	globalMeanRMSE	statistic	p.value	parameter	conf.low	conf.high
1	AU565	-0.0593	0.654	0.713	-3.7	2.62E-04	284	-0.0909	-0.0277
2	EFM19	-0.0551	0.658	0.713	-3.08	2.26E-03	268	-0.0902	-0.0199
3	HCC2218	0.246	0.959	0.713	10.8	2.82E-22	235	0.201	0.291
4	LY2	-0.0776	0.636	0.713	-5.46	9.28E-08	330	-0.105	-0.0496
5	MACLS2	-0.0531	0.66	0.713	-4.1	5.01E-05	372	-0.0785	-0.0277
6	MDAMB436	-0.00231	0.711	0.713	-0.177	8.60E-01	367	-0.028	0.0234
SC1: time vs global									
	time	deltaRMSE	groupMeanRMSE	globalMeanRMSE	statistic	p.value	parameter	conf.low	conf.high
1	0	0.0369	0.75	0.713	1.87	0.0622	233	-0.00189	0.0756
2	5.5	-0.016	0.697	0.713	-0.279	0.783	24.7	-0.134	0.102
3	7	-0.000796	0.712	0.713	-0.0362	0.971	183	-0.0442	0.0426
4	9	-0.00881	0.704	0.713	-0.438	0.662	191	-0.0485	0.0309
5	13	-0.00173	0.711	0.713	-0.0866	0.931	192	-0.0412	0.0378

6	17	-0.006	0.707	0.713	-0.305	0.76	194	-0.0447	0.0327
7	23	-0.037	0.676	0.713	-0.884	0.384	30.7	-0.122	0.0484
8	30	-0.0244	0.689	0.713	-0.537	0.595	30.4	-0.117	0.0682
9	40	0.00479	0.718	0.713	0.238	0.812	192	-0.0349	0.0445
	60	-0.0168	0.696	0.713	-0.843	0.4	193	-0.056	0.0224
SC1: treatment vs global									
	treatment	deltaRMSE	groupMeanRMSE	globalMeanRMSE	statistic	p.value	parameter	conf.low	conf.high
1	EGF	-0.0158	0.697	0.713	-1.01	0.314	449	-0.0465	0.015
2	full	0.0463	0.759	0.713	0.971	0.339	30.3	-0.051	0.144
3	iEGFR	0.00393	0.717	0.713	0.233	0.816	301	-0.0293	0.0371
4	iMEK	0.0127	0.726	0.713	0.737	0.462	295	-0.0212	0.0467
5	iPI3K	0.0033	0.716	0.713	0.185	0.854	288	-0.0319	0.0385
6	iPKC	-0.00438	0.709	0.713	-0.237	0.813	282	-0.0408	0.032

Appendix Table S2: comparison of RMSE within group versus global distribution via t-test. subchallenge 1

**Target Molecule**	**Inhibitor Name**	**Alternative Name**	**Supplier**	**Catalog Number**	**Concentration Used** [μM]
PI3K	GDC-0941	Pictilisib	LC Laboratories	G-9252	0.5
mTOR	Rapamycin	Sirolimus	LC Laboratories	R-5000	0.01
EGFR/HER2	Lapatinib		LC Laboratories	L-4899	1.08
PKC	Enzastaurin	LY317615	LC Laboratories	E-4506	3.9
MEK	CI-1040	PD184352	Selleckchem	S1020	1.7

Appendix Table S3: kinase inhibitors applied to single cell perturbation experiments.

Prediction methods of the best teams

Here we summarize the best performing methods for each subchallenge from the top 3 teams.

Subchallenge 1

Method of team icx_bxai (rank: 1)

Team members: Baosen Guo, Wencai Cao, He Shen, Xiangkun Gu, Peng Zhang, Qixun Qu, Jiamin Cao, Xiaoqing Jiang, Lisheng Zhou, Xiyan Xiang, Bin Xiao, Chao Liu, Linghao Shen, Hancheng Zheng

Subchallenge 1 was treated as a traditional regression task. The team started with the preprocessing of the data.

Data used for predictions:
Single cell proteomics, proteomics

Preprocessing
The single cell data from the same treatment, cell line and time are considered as one group. Four steps to build the dataset:

1. Z-score transformation.
2. Address irregularity in measurement time: the frequency of time in each group was different and some time did not appear in the target template. The team replaced some time points with low frequency: 12 /14 -> 13, 15 /16 ->17.
3. NA filtering: NAs were filtered from the training data.
4. Sampling: the amount of single cell in different groups were unbalanced, each group was resampled.

Modeling
Each target marker was modeled independently.
The included features were:

1. 32 given markers
2. One-hot encoded treatment and time
3. PCA of proteome
4. The medians of the current group and the one-time-pre/post group.

Different combinations of the features were used with a range of regression models including Elastic Net, ExtraTree, RandomForest, LGB and Ridge training and then averaged all predicting results as final prediction.

Code: https://www.synapse.org/#!Synapse:syn21217863
Language: python, library: sklearn, lightgbm

Method of team NAD (rank: 2)

Team members: Duc Tran, Hung Nguyen, Tin Nguyen
The team applied a convolutional neural network to learn the connection between inputs (the measured single cell markers) and outputs (missing single cell markers).

Data used for predictions:
Single cell proteomics

Preprocessing:

- Arrange the data by time for each batch of the NN
- Transformate the data: y = log10(asinh(x)+1)
- Subsample the data at each time point for the batches (256 cells per batch)

Modelling:

- Derive a separate model for each treatment condition and cell line
- Model features: only the 32 measured markers
- defined a convolutional neural network with 7 layers: a combination linear and Conv2d layers (see source code for details)
- Defined a squared loss function with l2 regularization

Code: https://www.synapse.org/#!Synapse:syn21265898
Language: python, library: pytorch

Method of team SingleCellLand (rank: 3)

Team members: Jie Zheng, Piyushkumar Mundra, Xinnan Dai, Jie Wang, Fan Xu
The team approached the prediction challenge as an imputation of the missing values problem.

Data used for the predictions:
Single cell proteomics

Preprocessing:

- Divided the training data into 5 subsets (5 folds).
- Removed any NAs

Modelling:

- Derived separate models for each missing marker and treatments (in total 30 models)
- Built a random forest based model (LightGBM).
- Model features: time and the 32 measured markers
- For each treatment and marker, 5 models were trained, by leaving out each fold from the training data. Finally the 5 models were averaged.

Code: https://www.synapse.org/#!Synapse:syn20821227/wiki/596104

Language: python, library: lightgbm

Subchallenge 2

Method of team icx_bxai (rank: 1)

Team members: Baosen Guo, Wencai Cao, He Shen, Xiangkun Gu, Peng Zhang, Qixun Qu, Jiamin Cao, Xiaoqing Jiang, Lisheng Zhou, Xiyan Xiang, Bin Xiao, Chao Liu, Linghao Shen, Hancheng Zheng

Subchallenge 2 was treated as a regression task, where the first goal was to predict the distribution of the cells (mean and covariance of markers) in the target group (defined by cell line, treatment, time). Then, a Gaussian sampler algorithm was used to predict the 10 000 single cells with the inferred mean and covariance matrices.

Data used for predictions:
Single cell proteomics

Preprocessing
- Z-score transformation
- Address irregularity in measurement time: the frequency of time in each group was different and some time did not appear in the target template. The team replaced some time points with low frequency: 12 -> 13, 16 ->17, 24 -> 23.
- Imputation: used interpolation for the missing mean values of markers at certain time points

Modeling steps:
- Algorithm: elasticNet,
- Modelled the mean and covariance of the markers independently
- Generated the single cell predictions using Gaussian distributions based on the inferred mean and covariances
- The included model features were a random subset of:
    - Mean and covariance values of the 35 measured markers of same cell line and same time
    - One-hot encoded treatment and time
    - Means, medians, quantiles from other treatments within the same cell line, same time or time 0.

The elasticNet algorithm was trained independently on the mean and covariance data with various parameters (regularization parameter alpha: {0.001,0.1,10}, l1_ratio: {0.3, 0.5, 0.7}) using subsets of model features. The model with each different tuning parameter and subset of features, were used to predict the mean and covariance matrices for the test condition. Then, the predicted mean values and the predicted covariances from the independent models were averaged and finally, the numpy.random.multivariate_normal() function was used to sample 10 000 cells following the predicted mean and covariance values.

Code: https://www.synapse.org/#!Synapse:syn21217863

Language: python, library: sklearn, numpy

Method of pqiu (rank 2)

Members: Peng Qiu
Pqiu argued that many cell lines show similar responses under the same perturbation and their responses to different perturbations could be correlated. Further, since the evaluation metric used the mean and covariance matrix, Pqui aimed to predict these parameters of the cells in the test conditions and then sampled with a multivariate Gaussian to generate the single cell predictions.

Data for the predictions:
Single cell proteomics

Modelling steps: (as written by the team)

- For a particular (cell line + unobserved perturbation + unobserved time point) to be predicted, (1) collect the samples corresponding to all (observed perturbation + observed time point) samples for this cell line, (2) compute the mean vector for each of these samples, (3) organize these mean vectors into a 37*K matrix, where K is the total number of observed (observed perturbation + observed time point) samples for this cell line, (4) let's call this matrix X2
- For each of 44 train cell line with complete data, (1) collect the samples corresponding to all the K observed (observed perturbation + observed time point) samples in the step above, (2) compute the mean vector for each of these samples, (3) organize these mean vectors into a 37*K matrix, (4) do this for all train cell lines with complete data, and obtain 44 such 37*K matrices, (5) concatenate all these matrices vertically to obtain a 1628*K matrix, (6) let's call this matrix X1
- For each of 44 train cell line with complete data, (1) collect the sample corresponding to the unobserved perturbation + unobserved time point in the first step above, (2) compute the mean vector for this sample, (3) this mean vector is of size 37*1, (4) do this for all train cell lines with complete data, and obtain 44 such 37*1 vectors, (5) concatenate all these matrices vertically to obtain a 1628*1 vector, (6) let's call this vector Y1
- Build a linear regression model based on X1 and Y1
- Apply the linear regression model to X2, and the resulting Y2 is the predicted mean vector for the particular (cell line + unobserved perturbation + unobserved time point) in the first step above
- Repeat all previous steps for each (cell line + unobserved perturbation + unobserved time point) to be predicted, which generates predictions of the mean vectors for all the samples to be predicted.
- Repeat all previous steps by replace mean vector by vectorized covariance matrices, which generates predictions of the covariance matrices for all the samples to be predicted.
- For each sample to be predicted, use the predicted mean and covariance to form a multivariate Gaussian distribution, randomly sample 10000 data points from the Gaussian, and use the sampled data points as submitted predictions

Code: https://www.synapse.org/#!Synapse:syn21326114
Language: MATLAB

Methods of team Orangeballs (rank 3)

Team members: Benjamin DeMeo, Alex Wu, Rohit Singh, Brian Hie

The Orangeball team observed substantial larger variance in the signaling between cell lines than within a cell line between different treatment conditions and time. Thus, when it was possible they built models that predict the response of cells in the unknown condition from the known conditions of the same cell line. However, they also noted that in spite of the inter-cell-line heterogeneity, the effect of a treatment was reasonably consistent among cell lines and including other cell lines to train the predictor drastically increased the performance of the predictions in the cross-validation scheme.

Data used for prediction
Single cell proteomics

Modelling steps (as written by the team)
In subchallenge 2, we predict the response of several cell lines to different kinase inhibitors. The expression profiles of these inhibitors are known for other cell lines; however, the base expression values in each of the cell lines varied substantially. By comparison, the response of a fixed cell line to varying treatments was remarkably similar. Thus, we used as a starting point the median and covariance of each cell-line's expression profile in the treatments known for that line. We then built a simple multilinear model that would predict the held-out treatment profiles given the remaining known ones. In all cases we trained separate models for each time point.
To make this mathematically rigorous, let $E(\ell,y,t,p)$ be the median expression of protein p in cell line ℓ after treatment y at time t. Let $y_1,...,y_6$ denote the six treatment conditions (including full). To predict an expression value $E(\ell_0,y_u,t,p)$ for an unknown treatment y_u, we use as a starting point the remaining known treatment values $\{E(\ell_0,y_i,t,p)\}$. We train a multilinear model using the cell lines for which all treatments are known. The inputs to this model are, for each line ℓ with all treatments known, an expression vector $E(\ell,\{y_1,...,y_6\},t,p)$, where y_u is excluded from the treatments. The corresponding outputs are the median values in the unknown treatments, $E(\ell,y_u,t,p)$. The result is a multilinear function from \mathbb{R}^5 to \mathbb{R}, which predicts the unknown treatment value given the remaining five. We then plug in these treatment values for the cell line of interest $\ell_0$to obtain our prediction for y_u. We observed empirically that most of the proteins did not change much between treatments -- simple linear regressions often had slope near 1. However, the multilinear model does substantially improve on simply plugging in treatment values, both on cross-validation and on scores in the leaderboard phase.
To predict covariances, we repeat the above approach for every pair of proteins p_i and p_j, predicting the covariance term $c_{ij}=cov(p_i,p_j)$ from the covariance measured in the other five known treatments, and using the remaining cell lines to train our model. Accounting for varying times, this results in over 10,000 models being fit. However, because each fit contains only 30-50 training samples (one for each viable cell line), the runtime is quite reasonable (around 10 minutes).

Code: https://www.synapse.org/#!Synapse:syn21339733
Language: python, library: sklearn

Subchallenge 3

Method of team icx_bxai (rank 1)

Team members: Baosen Guo, Wencai Cao, He Shen, Xiangkun Gu, Peng Zhang, Qixun Qu, Jiamin Cao, Xiaoqing Jiang, Lisheng Zhou, Xiyan Xiang, Bin Xiao, Chao Liu, Linghao Shen, Hancheng Zheng

Subchallenge 3 was approached similarly as subchallenge 2, where the first goal was to predict the distribution of the cells (mean and covariance of markers) in the target group (defined by cell line, treatment, time). Then, a Gaussian sampler algorithm was used to predict the 10 000 single cells with the inferred mean and covariance matrices.

Data used for predictions:
Single cell proteomics, prior knowledge network

Preprocessing

- Address irregularity in measurement time: Aimed at aligning time points with the [0, 7, 9, 13, 17, 40, 60], the pre-nearest time was used to replace the lacking time point. We replaced some time points: 24 -> 23, 25 /35 -> 40, 12 /14 -> 13, 15 /16 -> 17.

Modeling

- To predict a new treatment that was not seen before, they considered using correlation between different treatments and chose Line (https://github.com/tangjianpku/LINE) (a graph embedding framework, whose second order embedding can be applied to directed graph) to extract the correlation from the prior_knowledge.sif, which demonstrated the signaling network between protein kinases(treatments) and biomarkers
- Modelled the mean and covariance of the markers independently: the group mean and covariance were calculated by exponential weighted average of the value from other five treatments. The weights are correlation coefficients derived in the first step.
- Finally, the single cell predictions are generated by using Gaussian distributions based on the inferred mean and covariances

Code: https://www.synapse.org/#!Synapse:syn21217863
Language: python, library: LINE

Method of team Orangeballs (rank 2)

Team members: Benjamin DeMeo, Alex Wu, Rohit Singh, Brian Hie

The team tried several approaches to combine data of other conditions to predict the response to the treatment with mTOR inhibitor. The team discussed in their write-up, how the different approaches performed and improved their predictions across the challenge.

Data used for predictions
Single cell proteomics, prior knowledge network

Modelling steps (as written by the team)
We tried several different approaches. First, using the gene regulatory network from the Challenge wiki, we observed that PI3K and PKC were most directly related to mTOR. Thus, for each cell line, and each time post-treatment, we generated a mixture of cells from these two inhibitors, computed the mean and covariance, and used these to generate our predictions. This produced reasonable results, and landed us the top spot on the round 1 leaderboard for this subchallenge. Perhaps unsurprisingly, others performed better in round 2, spurring a search for more accurate methods.
Given the previously-discussed inter-cell-line heterogeneity, we opted to use only the expression values within the same cell line, but under other treatments, to predict mTOR values. For a more holistic view of how the treatments are related, we computed for each cell line, time, and pair of treatments the sum of the squared differences between the mean and covariance matrices. This is similar to the scoring metric used in the Challenge, although the latter only considers the upper triangle of the covariance matrix. The average treatment vs. treatment similarity across all cell lines and times is shown below.
From this we observe that EGF and iEGFR produce similar expression profiles, and that the full expression data is significantly different from any of the other treatments. Given that our submissions to the leaderboard had produced scores in the range of the full vs. treated scores, we hypothesized that mTOR more resembled the untreated data than any of the other treatments. However, substituting in the full values produced an abysmal score in round 2.
In preparing our final submission, we discovered that averaging together the expression profiles (mean and covariance) across all of the unknown cell lines produced better predictions than any of them independently. This suggests that simple mean imputation may outperform substitution, even when the most similar cell line is substituted, because the former is more robust to outliers. Excluding the full expression values from this mean improved cross-validation scores even more. The overall improvement over the above numbers was around 50%.
For each cell line and each time, we averaged the median and covariance across all treatments, with the exception of the full values. The resulting median and covariance values were used to generate the mTOR values for the given cell line and time.

Code: https://www.synapse.org/#!Synapse:syn21339733
Language: python

Method of team AMberland (rank 3)

Team members: Renata Retkute, Alidivinas Prusokas, Augustinas Prusokas

Data used for prediction
Single cell proteomics

Modelling (as written by the team)
For each cell line in the test dataset, we used marker data from the corresponding training data from *complete_cell_lines* directory. We have removed all rows with treaments *full* or *EGF* and put the rest of the data into a prediction matrix. Then for each time point, we randomly sampled 10,000 rows from the prediction matrix.

Code: https://www.synapse.org/#!Synapse:syn21302051
Language: R

Subchallenge 4

Method of team icx_bxai (rank: 1)

Team members: Baosen Guo, Wencai Cao, He Shen, Xiangkun Gu, Peng Zhang, Qixun Qu, Jiamin Cao, Xiaoqing Jiang, Lisheng Zhou, Xiyan Xiang, Bin Xiao, Chao Liu, Linghao Shen, Hancheng Zheng

Subchallenge 1 was treated as a traditional regression task. The team started with the preprocessing of the data.

Data used for predictions:
Single cell proteomics, median phosphoproteomics data

Preprocessing
The single cell data from the same treatment, cell line and time are considered as one group. Four steps to build the dataset:

- Z-score transformation.
- Address irregularity in measurement time: the frequency of time in each group was different and some time did not appear in the target template. The team replaced some time points with low frequency: 12 /14 -> 13, 15 /16 ->17,25 -> 23, 35 -> 40.
- Time imputation: interpolation for the lacking time point with the mean of two medians of the one-time-pre group and one-time-post group.

Modeling
Each target marker was modeled independently.
The included features were:

- One-hot encoded treatment and time
- Median, means and quantiles from the full treatment within the same cell line

Different combinations of the features were used with a range of regression models including Elastic Net, Ridge, SGD and SVR and then averaged all predicting results as final prediction.

Code: https://www.synapse.org/#!Synapse:syn21217863
Language: python, library: sklearn, lightgbm

Method of team AMberland (rank 2)

Team members: Renata Retkute, Alidivinas Prusokas, Augustinas Prusokas

The team used the median phosphoproteomics data to fit a linear regression, where the median expression of the target marker was assumed to be a function of the same marker under the treatment *full* and time point *0*.

Data used for prediction
median phosphoproteomics data

Modelling steps:

- Aggregated data at a particular time point from all available cell lines
- used values of the target markers from the training dataset under the treatment *full* and time point *0* and used the linear regression to predict target marker values
- took the median of these predicted values as output.

Code: https://www.synapse.org/#!Synapse:syn21302052
Language: R

Method of team orangeballs (rank 3)

Team members: Benjamin DeMeo, Alex Wu, Rohit Singh, Brian Hie

Data sued for prediction:
Median phosphoproteomics

Modeling steps: (as written by the team)
This subchallenge is similar in principle to subchallenge 2, but only the full, untreated expression values are known. Following a similar approach to that subchallenge, we fit linear models for each cell line, time, protein, and treatment, regressing the median untreated expression value of each protein against the median treated expression value. To predict the median expression of p_i in line ℓ_0 at time t under treatment y_i, we plug the median full expression value of protein p_i in line ℓ_0 into the corresponding regression line.
Again, we observe substantially improved performance (about a 2-fold improvement) over simply substituting in the full expression values. This again indicates that there is a meaningful relationship to learn between treated and untreated values. However, we observed that the majority of proteins were minimally affected by treatment, yielding regression lines with slope close to 1.

Code: https://www.synapse.org/#!Synapse:syn21339733
Language: python, library: sklearn

		comparison to reference model		comparison to random predictions		
**rank**	**team**	**statistic**	**p.value**	**statistic**	**p.value**	**N_data**
-	random_prediction	5.09E+09	1	-	-	119153
-	reference_model	-	-	2.01E+09	0	119153
1	icx_bxai	2.83E+09	0	1.81E+09	0	119153
2	NAD	2.95E+09	0	1.82E+09	0	119153
3	SingleCellLand	3.19E+09	1.04E-197	1.92E+09	0	119153

4	PaL	3.10E+09	0	1.88E+09	0	119153
5	Raghava_India_SCS	3.27E+09	1.05E-126	1.96E+09	0	119153
6	hulab.SCS	3.60E+09	0.999997173	2.11E+09	0	119153
7	Fire_Lily	3.37E+09	3.02E-53	1.98E+09	0	119153
8	Ostar	3.41E+09	4.57E-34	1.97E+09	0	119153
9	IGIBml	3.49E+09	3.87E-07	2.05E+09	0	119153
10	INLAB_BS	3.51E+09	0.00174254	2.04E+09	0	119153
11	orangeballs	3.30E+09	2.67E-100	1.97E+09	0	119153
12	msinkala	3.54E+09	0.185221	2.06E+09	0	119153
13	DUTeam	3.59E+09	0.999377317	2.07E+09	0	119153
14	hipathia	3.46E+09	9.72E-14	2.02E+09	0	119153
15	jamesxli	3.55E+09	0.650108933	2.05E+09	0	119153
16	AMbeRland	3.72E+09	1	2.09E+09	0	119153
17	dreamskys	3.66E+09	1	2.11E+09	0	119153
18	pqiu	3.88E+09	1	2.20E+09	0	119153
19	Sleeping	3.80E+09	1	2.19E+09	0	119153
20	SanGuo	4.16E+09	1	2.49E+09	0	119153
21	SCG	5.41E+09	1	3.91E+09	1	119153
22	Huiyuan	6.01E+09	1	4.88E+09	1	119153

Appendix Table S4: comparison of prediction accuracy to reference predictions and random predictions in SC1.
Test: Wilcoxon sign rank, which tests the distribution of the error produced by the teams' predictions vs the random and the reference model. The H0 hypothesis is that the distributions are the same, the alternative hypothesis is that the teams' predictions produce smaller errors ("less"). We compared the predictions on 119153 data points (1% of the total data)

		comparison to EGF prediction		Comparison to reference_model	

rank	team	statistic	p.value	statistic	p.value	N_data
-	reference_model	8.65E+08	1	-	-	55195
-	EGF prediction	-	-	6.58E+08	1.85E-169	55195
1	icx_bxai	5.81E+08	0	4.67E+08	0	55195
2	pqiu	6.66E+08	1.28E-144	5.74E+08	0	55195
3	orangeballs	6.58E+08	1.04E-167	5.41E+08	0	55195
4	PaL	8.03E+08	1	6.78E+08	5.60E-112	55195
5	NAD	9.18E+08	1	8.20E+08	1	55195
6	SCG	1.01E+09	1	8.78E+08	1	55195
7	AMbeRland	1.14E+09	1	9.94E+08	1	55195
8	Sleeping	1.27E+09	1	1.26E+09	1	55195
9	hipathia	1.26E+09	1	1.25E+09	1	55195
10	hulab.SCS	1.25E+09	1	1.25E+09	1	55195
11	CSBL	1.27E+09	1	1.27E+09	1	55195
12	msinkala	1.40E+09	1	1.37E+09	1	55195
13	Huiyuan	1.42E+09	1	1.40E+09	1	55195
14	SanGuo	1.47E+09	1	1.45E+09	1	55195
15	Raghava_India_SCS	1.48E+09	1	1.47E+09	1	55195
16	KAUST_RSS	1.48E+09	1	1.47E+09	1	55195

Appendix Table S5: comparison of prediction accuracy to reference predictions and EGF condition in SC2.

Test: Wilcoxon sign rank, which tests the distribution of the error produced by the teams' predictions vs the EGF condition and the reference model. The H0 hypothesis is that the distributions are the same, the alternative hypothesis is that the teams' predictions produce smaller errors ("less"). We compared the predictions on all the test data points (sample size: 55195)

		comparison to prediction by average cell line		comparison to reference model	
rank	team	statistic	p.value	statistic	p.value
-	reference_model	7.35E+06	4.03E-124	-	-
-	average_cell line	-	-	1.48E+07	1
1	icx_bxai	5.16E+06	0	7.16E+06	3.42E-137
2	AMbeRland	5.22E+06	1.15E-304	7.29E+06	2.67E-128
3	orangeballs	5.41E+06	1.58E-285	7.45E+06	4.91E-118
4	GaoGao199694	6.05E+06	1.36E-224	8.48E+06	4.97E-61
5	NAD	6.96E+06	4.87E-151	9.12E+06	2.34E-35
6	MacBookPro	7.29E+06	4.75E-128	9.40E+06	1.48E-26
7	PaL	8.97E+06	1.02E-40	1.12E+07	0.85849372
8	dreamskys	8.75E+06	2.19E-49	1.11E+07	0.51965076
9	pqiu	9.43E+06	1.15E-25	1.17E+07	0.99993359
10	ZeroPage_SCS	1.01E+07	2.91E-10	1.31E+07	1
11	DUTeam	1.01E+07	9.30E-11	1.44E+07	1
12	SanGuo	1.14E+07	0.99	1.39E+07	1
13	hulab.SCS	1.12E+07	0.83	1.38E+07	1
14	CSBL	1.08E+07	0.09	1.35E+07	1
15	Sleeping	1.18E+07	1.00	1.50E+07	1
16	msinkala	1.05E+07	5.58E-04	1.31E+07	1
17	hipathia	1.30E+07	1	1.57E+07	1
18	Raghava_India_SCS	1.40E+07	1	1.62E+07	1
19	KAUST_RSS	1.40E+07	1	1.62E+07	1
20	Huiyuan	1.61E+07	1	1.79E+07	1

Appendix Table S6: comparison of prediction accuracy to reference predictions and average cell line in SC4.
Test: Wilcoxon sign rank, which tests the distribution of the error produced by the teams' predictions vs the average cell line and the reference model. The H0 hypothesis is that the distributions are the same, the alternative hypothesis is that the teams' predictions produce smaller errors ("less"). We compared the predictions on all the test data points (sample size: 6650)

Single cell prediction on an independent dataset

The new dataset

We use the data from (Lun et al. 2017) to show the applicability of the prediction algorithm from the DREAM challenge winner (icx_bxai) on an independent data set.

In these series of experiments, Lun et al. studied the node abundance dependent signaling behavior of HEK293T cells. For that, in each experiment a selected gene was over-expressed and then the cells were perturbed by EGF stimulus. The cells were sampled in 5 time points: at the unstimulated state (0 min) and four times after stimuli (5, 15, 30 and 60 mins after stimuli) and 22 targeted molecular markers (phosphorylation sites and active form of proteins) were measured by mass cytometry.

The authors reported (Figure 3 (Lun et al. 2017)) strong signaling differences among groups of cells that overexpress different nodes in the RAS, MAPK and AKT pathways (Appendix Figure S9). Further, they observed strong signaling differences based on the level of overexpression (Figure 4 in (Lun et al. 2017)).

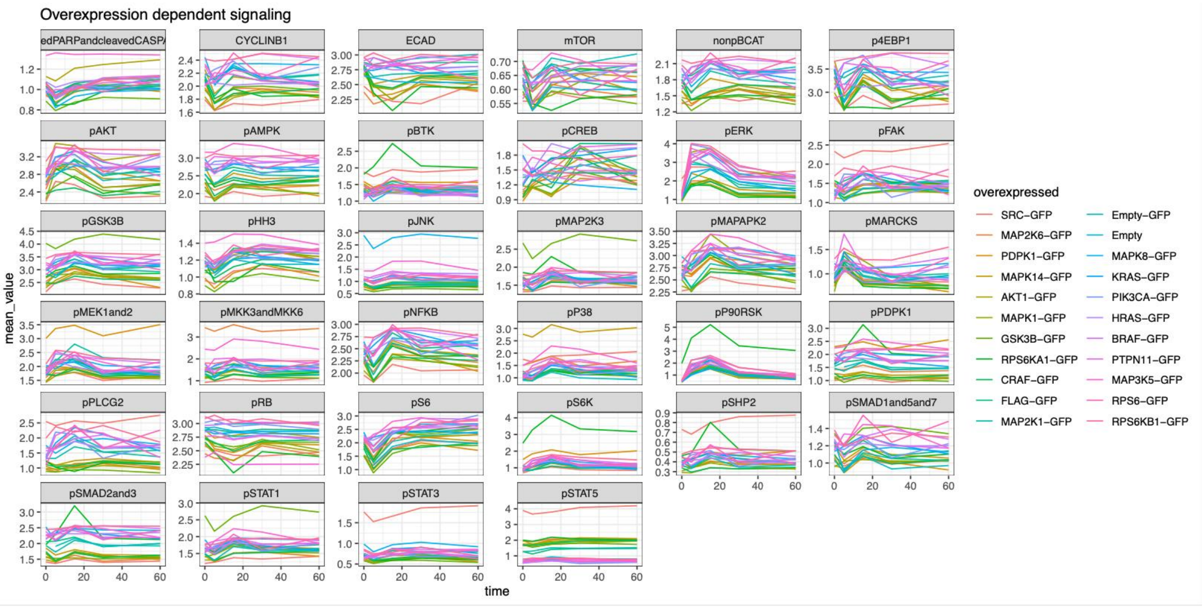

Appendix Figure S9. Overexpression dependent signaling.
Measured time courses of the cell lines that overexpressed a specific node in response to EGF stimulus. The raw data was transformed with the function y=asinh(x/5) and the single cell measurement were averaged in each experimental condition.

There are similarities between this dataset and the data used in the DREAM challenge:

- The single cell data is measured with mass-cytometry.
- The measured nodes partially overlap with the measured nodes in the challenge.
- The response was measured in the first hour.

However, there are also differences:

- The measurements are taken in response to EGF treatment, but there is no combinatorial treatment with kinase inhibitors.
- The cells were not starved before the stimuli.
- There is a single cell-line (HEK293T) with different nodes overexpressed, which alters the dynamic response.
- There is no proteomics, RNAseq or genomics data measured within this dataset.

The prediction task

For the validation of the prediction methods, we selected the following markers based on their dynamics (Appendix Figure S9) in the new dataset to be predicted:
- p-GSK3B, p-P90RSK: show good dynamics both in time and between groups
- p-4EBP1, p-AKT: shows good dynamics between groups, but less across time
- pPLCG2: flat trajectories for some overexpression groups, but changes for others

Similarly to the challenge, where we split the data to training and validation based on cell lines, here we split the data by overexpression groups (Appendix Table S7). This way the method has to predict the single cell response along the whole measured time-course.

Groups for training		Groups for testing	
FLAG-GFP	MAPK1-GFP	SRC-GFP	PIK3CA-GFP
Empty-GFP	AKT1-GFP	MAP2K6-GFP	BRAF-GFP
RPS6KA1-GFP	HRAS-GFP	PDPK1-GFP	RPS6KB1-GFP
GSK3B-GFP	MAPK8-GFP	MAPK14-GFP	
RPS6-GFP	PTPN11-GFP	CRAF-GFP	
KRAS-GFP	MAP3K5-GFP	MAP2K1-GFP	

Appendix Table S7. Overexpression groups assigned to training and test sets.

Prediction method

The challenge winning group (icx_bxai) was asked to train their method on the training set and provide predictions for the test data. Due to the mentioned difference between the Challenge and this dataset, the method had to be slightly modified:

- In terms of Feature Engineering, the method is basically unchanged.
- Only time is one hot encoded (no kinase inhibitors in this data)
- "Overexpressed" and "time" were defined as the basis for cell grouping.
- The median values of the markers from the same "overexpressed" group at the current time point (t), the previous time point (t-1) and the next time point (t+1) were used as the time-series statistical features.
- Because of the lack of omics data, PCA components of 30 given markers were used instead.

Evaluation of the predictions

We used the same metric (averaged RMSE across the overexpression groups) to evaluate the prediction as in subchallenge 1, which resulted in a value of $RMSE_{team,Lun}$=0.508. Further, we also evaluated a random model (same procedure as in subchallenge 1, see main text), which gave $RMSE_{random,Lun}$ = 1.298.

Since this score could depend on the selection of markers, and overexpression groups, we used bootstrap method and sampled the conditions (defined by the set of overexpressed node, predicted marker and time) in the test data 1000 times and evaluated both the prediction of the team and the random prediction (Appendix Figure S8)

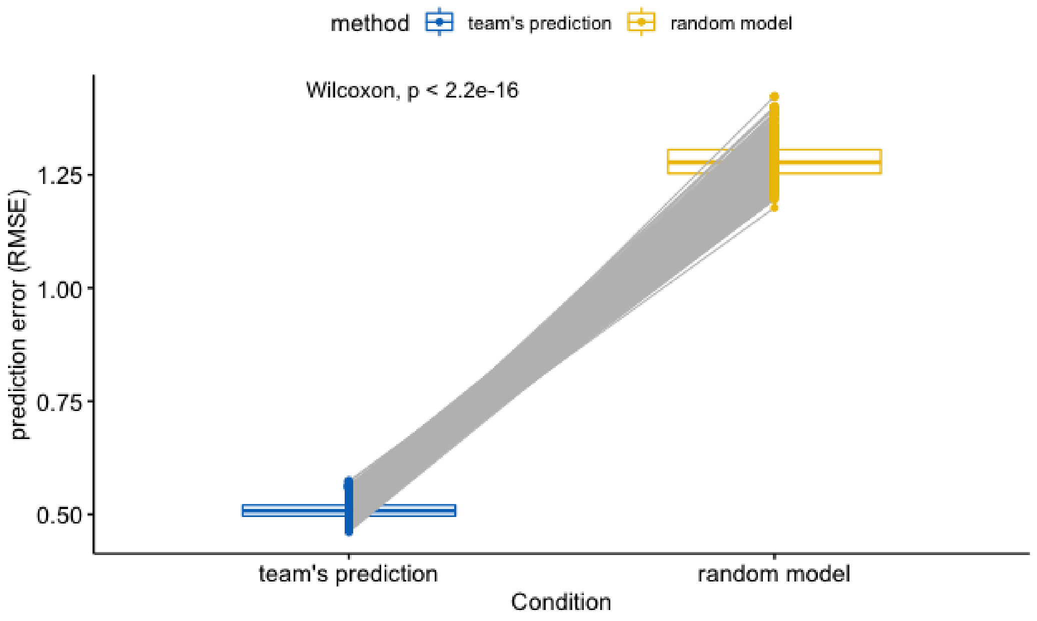

Appendix Figure S10. Comparison of team's prediction with random predictions on an independent dataset.

We compared the predictions on 1000 bootstrap (paired) samples and compared the distribution of the prediction errors by Wilcoxon signed ranked test), which shows strong

(reduction in error: 0.771) and significant (p.val< 1e-16) difference in favour of the teams prediction.

In comparison to the Subchallenge 1, here the random prediction performed similarly ($RMSE_{random,Lun}$ = 1.298 vs. $RMSE_{random,SC1}$ = 1.44,), but the difference between the real predictions in the two datasets ($RMSE_{team,Lun}$ = 0.508 vs. $RMSE_{team,SC1}$ = 0.849) are much stronger. The small difference in random predictions indicates that new data from Lun et al. has similar characteristics, maybe less changes between the single cells. Further, the increased performance of the model in the new data (40% less error) is likely because the groups of cells overexpressing different nodes are more similar to each other, than the different breast cancer cell lines in the challenge.

DREAM consortium

First name	Last name	Team	Affiliation
Augustinas	Prusokas	AMbeRland	Imperial College London
Alidivinas	Prusokas	AMbeRland	Newcastle University
Renata	Retkute	AMbeRland	University of Cambridge
Anand	A. R.	CSBL	Department of Biotechnology, Bhupat Jyoti Mehta School of Biosciences
Karthik	Raman	CSBL	Department of Biotechnology, Indian Institute of Technology Madras
Malvika Sudhakar		CSBL	Department of Biotechnology, Bhupat Jyoti Mehta School of Biosciences
Raghunathan	Rengaswamy	CSBL	Initiative for Biological Systems Engineering (IBSE)
Edward S.C. Shih		dreamskys	Institute of Biomedical Sciences, Academia Sinica, Taipei, Taiwan, R.O.C.
Min-jeong	Kim	DUTeam	National Evidence-based Healthcare Collaborating Agency
Changje	Cho	DUTeam	ChangJe@synapse.org
Dohyang	Kim	DUTeam	Division of Mathematics and Big Data Science, Daegu University, South Korea
Hyeju	Oh	DUTeam	Division of Mathematics and Big Data Science, Daegu University, South Korea
Jinseub	Hwang	DUTeam	Division of Mathematics and Big Data Science, Daegu University, South Korea
Kim	Jongtae	DUTeam	Division of Mathematics and Big Data Science, Daegu University, South Korea
Yeongeun	Nam	DUTeam	Division of Mathematics and Big Data Science, Daegu University, South Korea

Sanghoo	Yoon	DUTeam	Division of Mathematics and Big Data Science, Daegu University, South Korea
Taeyong	Kwon	DUTeam	Division of Mathematics and Big Data Science, Daegu University, South Korea
Kyeongjun	Lee	DUTeam	Division of Mathematics and Big Data Science, Daegu University, South Korea
Sarika	Chaudhary	Fire_Lily	M.Tech Computer science, Indraprastha Institute of Information Technology, Delhi
Nehal	Sharma	Fire_Lily	M.Tech Computer science, Indraprastha Institute of Information Technology, Delhi
Shreya	Bande	Fire_Lily	M.Tech Computer science, Indraprastha Institute of Information Technology, Delhi
Gao	Gao	GaoGao199694	Chongqing Institute of Green and Intelligent Technology, Chinese Academy of Sciences
Cankut	Cubuk	hipathia	The Clinical Bioinformatics Area
Pelin	Gundogdu	hipathia	FPS
Joaquin	Dopazo	hipathia	Bioinformatics Area, FPS
Kinza	Rian	hipathia	Bioinformatics Area, FPS
Carlos	Loucera	hipathia	Clinical Bioinformatics Area
Matias	M. Falco	hipathia	Mfalco@synapse.org
Martin	Garrido-Rodriguez	hipathia	mgrcbioinfo@synapse.org
Maria	Peña	hipathia	mpena@synapse.org
Huiyuan	Chen	Huiyuan	Case Western Reserve University
Gabor	Turu	hulab SCS	Department of Physiology, Faculty of Medicine, Semmelweis University, Budapest, Hungary
László Hunyady		hulab SCS	Department of Physiology, Faculty of Medicine, Semmelweis University, Budapest, Hungary
Ádám Misák		hulab SCS	Department of Physiology, Faculty of Medicine, Semmelweis University, Budapest, Hungary
Baosen	Guo	icx_bxai	Division of AI & Bioinformatics, Shenzhen Digital Life Institute, Shenzhen, China
Wencai	Cao	icx_bxai	Division of AI & Bioinformatics, Shenzhen Digital Life Institute, Shenzhen, China
He	Shen	icx_bxai	Division of AI & Bioinformatics, Shenzhen Digital Life Institute, Shenzhen, China
Lisheng	Zhou	icx_bxai	Division of Diabetes, Shenzhen iCarbonX Digital Life Management Co., Ltd, Shenzhen, China
Xiaoqing	Jiang	icx_bxai	Division of Diabetes, Shenzhen iCarbonX Digital Life Management Co., Ltd, Shenzhen, China

Pieta	Zhang	icx_bxai	Division of AI & Bioinformatics, Shenzhen Digital Life Institute, Shenzhen, China
Aakansha	Rai	IGIBml	CSIR-Institute of Genomics & Integrative Biology
Rintu	Kutum	IGIBml	CSIR-Institute of Genomics & Integrative Biology
Sadhna	Rana	INLAB_BS	Innovation Labs, Tata Consultancy Services
Rajgopal Srinivasan		INLAB_BS	Innovation Labs, Tata Consultancy Services
Swatantra	Pradhan	INLAB_BS	Innovation Labs, Tata Consultancy Services
James	Li	jamesxli	VisuMap Technologies Inc.
Vladimir	Bajic	KAUST_RSS	King Abdullah University of Science and Technology, CBRC.
Christophe Van Neste		KAUST_RSS	King Abdullah University of Science and Technology, CBRC.
Didier	Barradas-bautista	KAUST_RSS	King Abdullah University of Science and Technology, Catalysis center.
Somayah Abdullah Albarade		KAUST_RSS	King Abdullah University of Science and Technology, CBRC.
Igor	Nikolskiy	MacBookPro	igor.nikolskiy@synapse.org
Musalula	Sinkala	msinkala	School of Health Sciences, Department of Integrative Biomedical Sciences, Computational Biology Division, University of Cape Town
Duc	Tran	NAD	Department of Computer Science and Engineering, University of Nevada, Reno.
Hung	Nguyen	NAD	Department of Computer Science and Engineering, University of Nevada, Reno.
Tin	Nguyen	NAD	Department of Computer Science and Engineering, University of Nevada, Reno.
Alex	Wu	orangeballs	alexw@synapse.org
Benjamin DeMeo		orangeballs	bdemeo@synapse.org
Brian	Hie	orangeballs	brianhie@synapse.org
Rohit Singh		orangeballs	rs239@synapse.org
Jiwei	Liu	Ostar	AI infrastructure, NVIDIA
Xueer	Chen	Ostar	, Department of Biomedical Informatics, University of Pittsburgh
Leonor Saiz		PaL	Department of Biomedical Engineering, University of California, 451 East Health Sciences Drive, Davis, California 95616, USA
Jose M. G. Vilar		PaL	Biofisika Institutua (CSIC, UPV/EHU), University of the Basque Country, P.O. Box 644, 48080 Bilbao, Spain

Peng	Qiu	pqiu	Department of Biomedical Engineering, Georgia Institute of Technology and Emory University
Akash	Gosain	Raghava_India_SCS	Indraprastha Institute of Information technology, Delhi
Anjali Dhall		Raghava_India_SCS	Indraprastha Institute of Information Technology, Delhi.
Dinesh Bajaj		Raghava_India_SCS	Indraprastha Institute of Information Technology, Delhi
Harpreet	Kaur	Raghava_India_SCS	CSIR-Institute of Microbial Technology, Chandigarh
Krishna	Bagaria	Raghava_India_SCS	Indraprastha Institute of Information Technology, Delhi.
Mayank	Chauhan	Raghava_India_SCS	Indraprastha Institute of Information Technology, Delhi.
Neelam Sharma		Raghava_India_SCS	Indraprastha Institute of Information Technology, Delhi.
Gajendra	Raghava	Raghava_India_SCS	Indraprastha Institute of Information Technology, Delhi
Sumeet	Patiyal	Raghava_India_SCS	Indraprastha Institute of Information Technology, Delhi
Jianye Hao		SanGuo	Tianjin University
Jiajie	Peng	SanGuo	Northwestern Polytechnical University
Shangyi Ning		SanGuo	Fudan University
Yi Ma		SanGuo	Tianjin University
Zhongyu	Wei	SanGuo	Fudan University
Atte	Aalto	SCG	Luxembourg Centre for Systems Biomedicine, University of Luxembourg
Jorge	Goncalves	SCG	Luxembourg Centre for Systems Biomedicine, University of Luxembourg
Laurent	Mombaerts	SCG	Luxembourg Centre for Systems Biomedicine, University of Luxembourg
Xinnan	Dai	SingleCellLand	School of Information Science and Technology, ShanghaiTech University, Shanghai 201210, China.
Jie	Zheng	SingleCellLand	School of Information Science and Technology, ShanghaiTech University, Shanghai 201210, China.
Piyushkumar	Mundra	SingleCellLand	Cancer Research UK Manchester Institute, Manchester, UK.
Fan	Xu	SingleCellLand	School of Information Science and Technology, ShanghaiTech University, Shanghai 201210, China.
Jie	Wang	SingleCellLand	School of Information Science and Technology, ShanghaiTech University, Shanghai 201210, China.

Krishna Kant	Singh	Sleeping	Data scientist, Aryballe , Grenoble, France
Mingyu	Lee	ZeroPage_SCS	Kakao corp, South Korea.
