# Supplementary material for: Cell‐to‐cell and type‐to‐type heterogeneity of signaling networks: insights from the crowd
Source: Mol Syst Biol. 2021 Oct 18;17(10):e10402. doi: 10.15252/msb.202110402 (PMC8522707; doi:10.15252/msb.202110402)
Supplement: Supplementary file 2 [file MSB-17-e10402-s001.pdf]

molecular systems biology

Cell-to-cell and type-to-type heterogeneity of signaling networks: Insights from the crowd

Attila Gábor, Marco Tognetti, Alice Driessen, Jovan Tanevski, Baosen Guo, Wencai Cao, He Shen, Thomas Yu, Verena Chung, Bernd Bodenmiller, and Julio Saez-Rodriguez
DOI: 10.15252/msb.202110402

Corresponding author(s): Julio Saez-Rodriguez (pub.saez@uni-heidelberg.de) , Bernd Bodenmiller (bernd.bodenmiller@uzh.ch)

Review Timeline:

Submission Date:	13th Apr 21
Editorial Decision:	28th May 21
Revision Received:	24th Aug 21
Editorial Decision:	15th Sep 21
Revision Received:	27th Sep 21
Accepted:	28th Sep 21

Editor: Maria Polychronidou

Transaction Report:

(Note: With the exception of the correction of typographical or spelling errors that could be a source of ambiguity, letters and reports are not edited. Depending on transfer agreements, referee reports obtained elsewhere may or may not be included in this compilation. Referee reports are anonymous unless the Referee chooses to sign their reports.)

Thank you again for submitting your work to Molecular Systems Biology. We have now heard back from the three referees who agreed to evaluate your study. Overall, the reviewers acknowledge that the results of the challenge are a relevant contribution to the field. They raise however a series of concerns, which we would ask you to address in a major revision.

Without repeating all the points raised, some of the more fundamental issues are the following:

- As reviewers #1 and #3 point out, the study would benefit from a more detailed method presentation.
- Interpretations of the results in the context of the underlying biology of signaling networks should be included as suggested by reviewer #2.
- The reviewers also raise several technical issues and request the inclusion of clarifications and more detailed discussion of several aspects of the analyses and results.
- Analyses of an independent dataset as suggested by reviewer #1 would significantly enhance the impact of the study.

All issues raised by the reviewers need to be satisfactorily addressed. Please contact me in case you would like to discuss in further detail any of the issues raised.

On a more editorial level, we would ask you to address the following points:

Reviewer #1:

The paper should be published. However, the manuscript would benefit a lot if it would present the individual methods participating. The reviewer understands these can be published as independent units however it leaves the reader in the darkness. Its great to see the methods challenged and benchmarked but the manuscript would catch more readers attention if it would also explain the basis for at least some of the methods.

Secondly, to which degree the benchmarking is shown to be robust and significant? Its not clear to which degree methods have been benchmarked with AROC and also shown the improvements (or differences) are significant (p-Values), or better a Bayesian based benchmarking.

Thirdly, there is a dataset published on kinase-inhibitors/proteomics from Cutillaslab (https://www.nature.com/articles/s41587-019-0391-9) that could be very relevant for this challenge. The manuscript would benefit a lot from this data being evaluated in the context of the top performing methods. It would serve at least as an independent dataset. It also gets to the issue of kinase inhibitor specificity and off-target effects which is not really discussed in the current version of the manuscript.

Finally the paper would benefit from more involvement of signaling network modelling groups and cell signaling labs and with different types of data. The authors are encouraged to develop this further.

Reviewer #2:

Gabor and Tognetti et al present a fascinating paper describing the recent results of Single Cell Signaling in Breast Cancer (SCSBrC) Dialogue for Reverse Engineering Assessments and Methods (DREAM) challenge. The study aims to leverage a vast single-cell training set to understand whether computational models can predict cell signaling responses in unmeasured (non-empirical) nodes. This is an important computational problem as existing single-cell proteomic technologies are limited by reporter channels (~50 in mass cytometry) or the availability well validated anti-PTM affinity reagents. If computational methods could accurately impute/predict unmeasured datapoints this could greatly expand our knowledge of signaling biology.

The paper is generally very well written and cleanly divided into 4 subchallenges (beautifully illustrated in Figure 1). Each subchallenge is clearly introduced and discussed. I thoroughly enjoyed reading the paper and have very few suggestions for improvement. This is an exceptional manuscript and I congratulate the authors on their excellent work.

Minor Points:

1) One important finding is that models combining multiple methods typically perform better than simpler alternatives. Is there some way to illustrate this when introducing each model (e.g. in Figure 2A)? The currently figure doesn't reveal ANY differences regarding the different models which hides this take-away message.

2) What does the success of these models tell us about the underlying biology of signaling networks? Is the fact that signaling is predictable mean that signaling networks are highly ordered? Is the ultimate source of model failure cell-type-specific difference or non-canonically wired networks? Some discussion regarding the fundamental implications of these findings on signaling biology (i.e. not the models themselves) would be very valuable to readers.

3) Are there model types/formats that weren't used by contestants that theoretically might be better for future versions of such challenges? For example, 'machine learning' is frequently mentioned but not neural networks?

4) It is increasing appreciated that the cell-cycle has a huge effect on signaling networks. If all cell-cycle markers are removed for the training/test data, how do you think the models would have performed? Does this imply that cell-cycle markers are 'essential' elements of predicting signaling and should be included in all future signaling studies?

Reviewer #3:

Gabor et al. and the Single Cell Signaling in Breast Cancer DREAM Consortium members reported the related DREAM challenge results. They use a large mass cytometry dataset that measures single cell dynamic signaling responses of 37 markers to EGF+"serum" over 1 hour across 67 breast cancer cell lines in the presence or absence of 5 different pathway inhibitors. In addition, they report "basal" proteomics (~5000 quantified proteins) and genomics (mRNAseq, CNV, SNP) data. The dataset alone is a substantial contribution to the literature, although the extent to which it is already available in the cited work was not clear. The challenge involving this dataset was split into four parts called subchallenges (SC). First, they asked participants to predict a subset of 5 markers for 6 cell lines, based on all the other data. The next two challenges asked participants to predict effects of inhibitors on the dynamic responses. In the 2nd, prediction was for 12 of the cell lines, given data for inhibitor response in the other cell lines. The prediction was only a single drug in each line, not all the drugs in each of the 12 lines. In the 3rd, the prediction was for a mTOR inhibitor, without having data on the mTOR inhibitor (besides the name and concentration), but using all the other data. The fourth challenge was to predict dynamics in response to EGF (plus serum) in 5 of the cell lines, given all the data, plus the "basal" data.
At the end of four sub-challenges (SCs), they conclude that the machine learning methods, especially a collection of them, can accurately predict single-cell perturbation responses. As denoted in their paper, there are not enough resources to do all the desired experiments or the current knowledge to intelligently select the most informative ones. Efforts like the one presented here, with accompanying datasets, will benefit and enable the biomedical research community in this regard greatly. In particular, the result that the cell-line specific signaling dynamics (at least over 1 hr time course) can be predicted from "basal" information is particularly compelling. That being said, I do have substantial concerns about the manuscript in its current form, that are detailed below.

Major Points:
--An early summary figure of teams, their methodologies, and overall performances across different SCs will benefit the readers and make the text easier to understand before going into the Results section. Importantly, the participants were allowed to use prior knowledge, but how that was implemented and/or impactful was missing from the paper.
--The experimental methods could not be located in the paper. A detailed methods section is absolutely essential since a major contribution of this paper is the dataset (depending on the cited work).
--Along the previous lines, it seems that the authors treated cells with EGF + serum (based on supplementary figure 2). The paper continually refers to EGF treatment. This discrepancy needs to be resolved. I could not decipher what was the case because I could not find detailed experimental methods.
--Another methodological point. Cytof requires suspension cells, but these all seem to be adherent. That needs to be reported and discussed. Adhesion often contributes strongly to signaling.
--I am not convinced by the authors claim that non-canonical MEK-independent ERK signaling can explain why models do not predict ERK signaling well in certain cell lines. The main reason are the data presented in Supplementary Figure 4, along with Supplementary Table 3. In the six cell lines shown in Fig. S4, the MEK inhibitor seems to have quite incomplete effects on pERK dynamics. Table S3 shows a non-clinical MEK inhibitor is used, at an abnormally high concentration for MEK inhibitors. Therefore it may be that issues with the inhibitor dose and / or the inhibitor itself may account for the observations. That being said, the paper is strong without going into details on MEK-independent ERK signaling. But, I do believe a rewording of all such claims in the paper is warranted. Likewise, it seems as though some of the other inhibitors may not be working as well as the authors may think.
--Along the lines of the above point, on pg 14, the authors state "This relatively good score is due to the fact that the kinase inhibitors are very specific and influence only a small number of nodes". I am not sure why the authors are making this statement, but it is quite well established that kinase inhibitors show significant promiscuity, especially at some doses reported in Table S3.
--The dense time points chosen within the first hour of treatment response may not be indicative of the eventual phenotypic response such as cell cycle or cell death. The work remains significant, yet some discussion of the relation between dynamics, signaling and the phenotypes that are actually of final interest to predict is lacking.
--The claims of the paper on the surface seem quite significant, related to the fact that signaling dynamics and their inhibitor responses can be predicted reasonably well on a large scale. Yet, there are some aspects of this that I find somewhat lacking. For example, usually a small % of the dataset is predicted-in SC1 5 markers in 6 cell lines out of 37 markers and 67 cell lines, with "prior knowledge" allowed (what this means is opaque-see above comment). What is the underlying dimensionality of the datasets (e.g simple PCA)? Is this surprising from a statistical or information perspective? From a biological perspective, the cytof panel is covering multiple parts of pathways that are expected to be highly correlated. For example, if I know what pMEK is doing, it is highly likely I know what pERK is doing. That kind of analysis and discussion is what I am referring to.
--Along the above lines, usually test and training data are parsed many times over to get a real estimate of predictability. Granted, that is not really possible in this DREAM challenge scenario, but the lack of it diminishes the claims of how predictable things are.
--There is some hype with respect to the single-cell nature of this dataset and prediction. But the only true single-cell predictions seem to be in SC1. The remaining challenges focus on predicting mean and/or variance of the population. That discussion should be more balanced.
--The authors' conclusion that the time resolution (or the number of time points) can be sacrificed first to reduce the number of experiments is not that well supported yet. Per their data and accompanying performance of the teams' models to predict time

points better do not necessarily mean that the dynamical data are of lesser importance compared to more cell lines or more conditions (perturbations) in each cell line. One reason is that they also point out multiple times that the models predicted conditions of non-time-varying markers across cell lines better compared to other conditions. So, I believe without an analysis where the authors iteratively train and test model predictions at different time resolutions, their claim on this matter is not well supported.
--to better understand what was done in this manuscript, the information in supplementary figure 2 would be quite useful in figure 1. The proportions in the cartoons of the 2nd row of figure 1 are not representative of what was done.
--A major finding of this paper is the claim that signaling dynamics can be predicted from basal information. Some of that data is presented in Figure 5D. While there are cases where the predictions are good, there are other cases where the predictions seem quite wrong. So it is unclear how supported that claim is.
-- The comparison of model predictions to biological replicate variance may be overstated. Mostly because there were only the two replicates (from what I could see), the emphasis here seemed distracting in the flow of the paper.
-- In the discussion (2nd paragraph), the authors speak to the transferability of inferred reactions / connections between cell lines. This is biologically not surprising, but a further discussion on how one could determine which signaling interactions are more transferrable than others would be biologically useful.
Minor Points:
--The figure quality could be improved. Often times text is very small, and/or "stretched".
--pg 9. "maker" marker
--lapatinib is a dual EGFR/HER2 inhibitor, not an EGFR inhibitor.

Reviewers' comments:

Reviewer #1:

1) The paper should be published.

Thank you for your positive opinion on our paper.

However, the manuscript would benefit a lot if it would present the individual methods participating. The reviewer understands these can be published as independent units however it leaves the reader in the darkness. Its great to see the methods challenged and benchmarked but the manuscript would catch more readers attention if it would also explain the basis for at least some of the methods.

Thank you for pointing out this issue. We agree with this comment and to address it, we have added
(1) more details on the similarities and differences of methods for the result section on subchallenge 1 (SC2/3/4 already included in the previous version) and
(2) a new section to the Appendix (supp material) of the updated manuscript: "Prediction methods of the best teams", where the methods of the top 3 teams in each subchallenge are summarised and referenced it from the main text:

"To understand what makes a model better than others, we analysed the participants' write-up of methods and their answers to a questionnaire (Appendix File 1). The best three teams approached the prediction task with different methods: an ensemble of linear and tree-based methods, convolutional neural networks, and gradient boosting, respectively (details of each method are available in the Appendix). Further, while team 1 and 3 built models for each missing marker separately, the neural network model of team 2 predicted all the 5 missing markers together. Common among the three approaches are that all of them included all the 32 measured markers as model features among others and each team addressed the problem of missing data. "

"Interestingly, five teams, including the top three (icx_bxai, pqui and orangeballs) followed similar modelling approaches for SC2 (more about the approaches in the Appendix)."

"The top three participants outperformed the average cell line model (baseline model) for all the markers (Figure 5B) except for GAPHD, for which their prediction error was similar to the average cell line model (prediction approaches are summarised in Appendix)."

2) Secondly, to which degree the benchmarking is shown to be robust and significant? It's not clear to which degree methods have been benchmarked with AROC and also shown the improvements (or differences) are significant (p-Values), or better a Bayesian based benchmarking.

We agree that the robustness and significance have to be evaluated. For robustness, we used bootstrapping, where the test data conditions were sampled 1000 times. The predictions from each team were evaluated on each sample. The distributions of the scores can be seen on Fig 2 panel A-D by the violin plots in each subchallenge.

In the revised manuscript, we added the bootstrap distributions for the reference and random models for better visual comparison. Please note that these panels of figures are now shown on Figure 2 in the revised manuscript.

Updated fig2A:

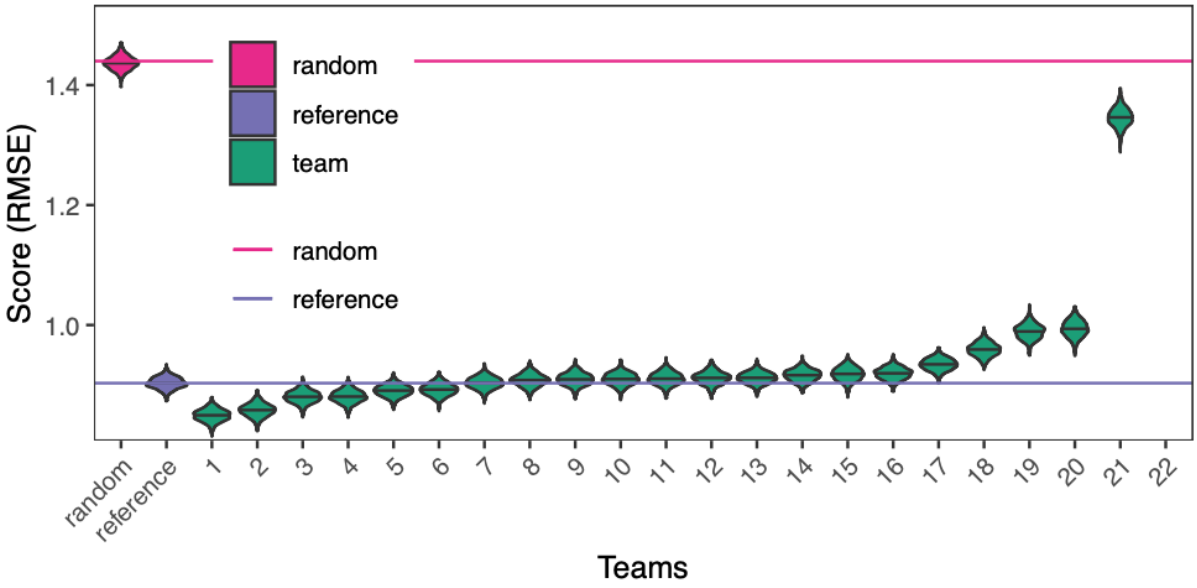

Updated fig4A:

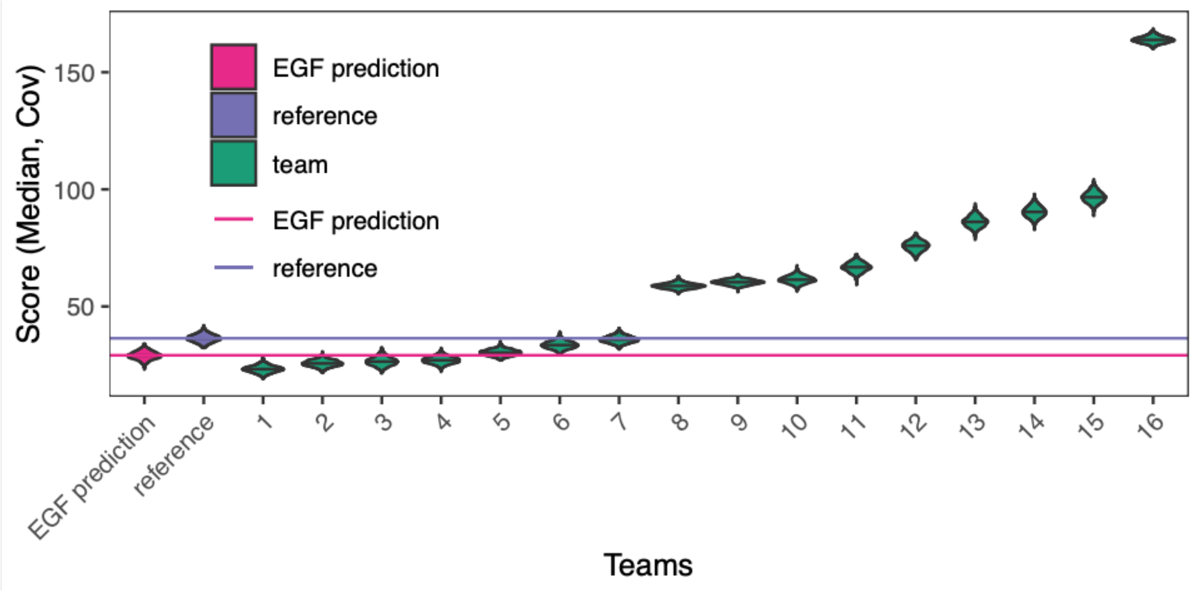

Updated fig5A:

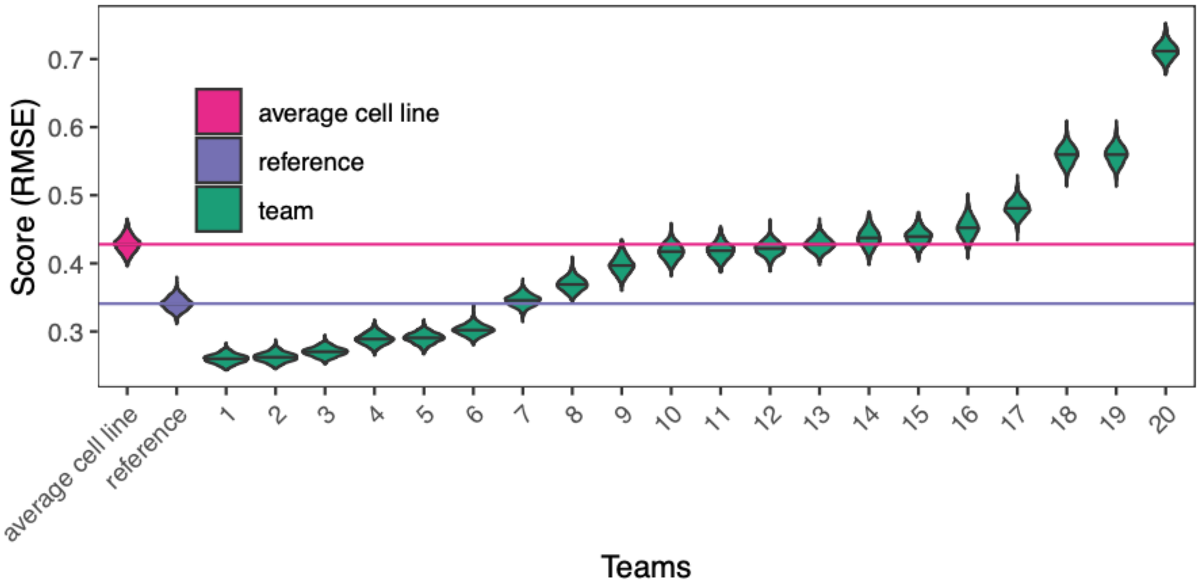

Based on the distributions of these scores, we calculated the Bayes factor between each pair of consecutively ranked teams. This method showed that the ranking of the teams was robust against variation of the test data (Area under the receiver operator characteristic curve (AUROC) applies to classification problems, however, all the subchallenges are addressing regression problems)

Regarding the significance: in each subchallenge, we defined reference and random (null) models, but indeed, in the original manuscript we didn't use a statistical approach to compare it with the teams' prediction. In the revised manuscript we added non-parametric statistical tests (Wilcoxon sign rank) to see if the individual teams' predictions have significantly lower errors than the reference model or the random predictions (See Appendix Table 4, 5 and 6). The results are in accordance with the previous findings: the top 3 teams

performed significantly better than our reference model and most of the teams performed better than random.

3) Thirdly, there is a dataset published on kinase-inhibitors/proteomics from Cutillaslab (https://www.nature.com/articles/s41587-019-0391-9) that could be very relevant for this challenge. The manuscript would benefit a lot from this data being evaluated in the context of the top performing methods. It would serve at least as an independent dataset. It also gets to the issue of kinase inhibitor specificity and off-target effects which is not really discussed in the current version of the manuscript.

The mentioned manuscript presentes indeed an excellent data set, also dealing with signaling networks in cancer and upon drug perturbation. That said, it is based on bulk phosphoproteomics data. Compared to the mass cytometry used in the challenge, the coverage is much larger, but it is untargeted (that is, not the same phospho-sites are measured in each experiment), and it does not have single-cell resolution. Given the stark differences, it would not be appropriate to apply directly the methods of this challenge. We would actually be very excited to run a similar challenge focused on mass-spec phosphoproteomics.

That said, we fully agree that analysing an independent data set would be important to show the general value of the models. For this, in the revised version we applied the winning method on an independent dataset from:

Lun, X. K., Zanotelli, V. R., Wade, J. D., Schapiro, D., Tognetti, M., Dobberstein, N., & Bodenmiller, B. (2017). Influence of node abundance on signaling network state and dynamics analyzed by mass cytometry. *Nature biotechnology*, 35(2), 164–172. https://doi.org/10.1038/nbt.3770

The results are summarised in the Appendix section "Single cell prediction on an independent dataset". In essence, the method of the winning team greatly outperforms a random model (see figure below, Appendix Figure S8).

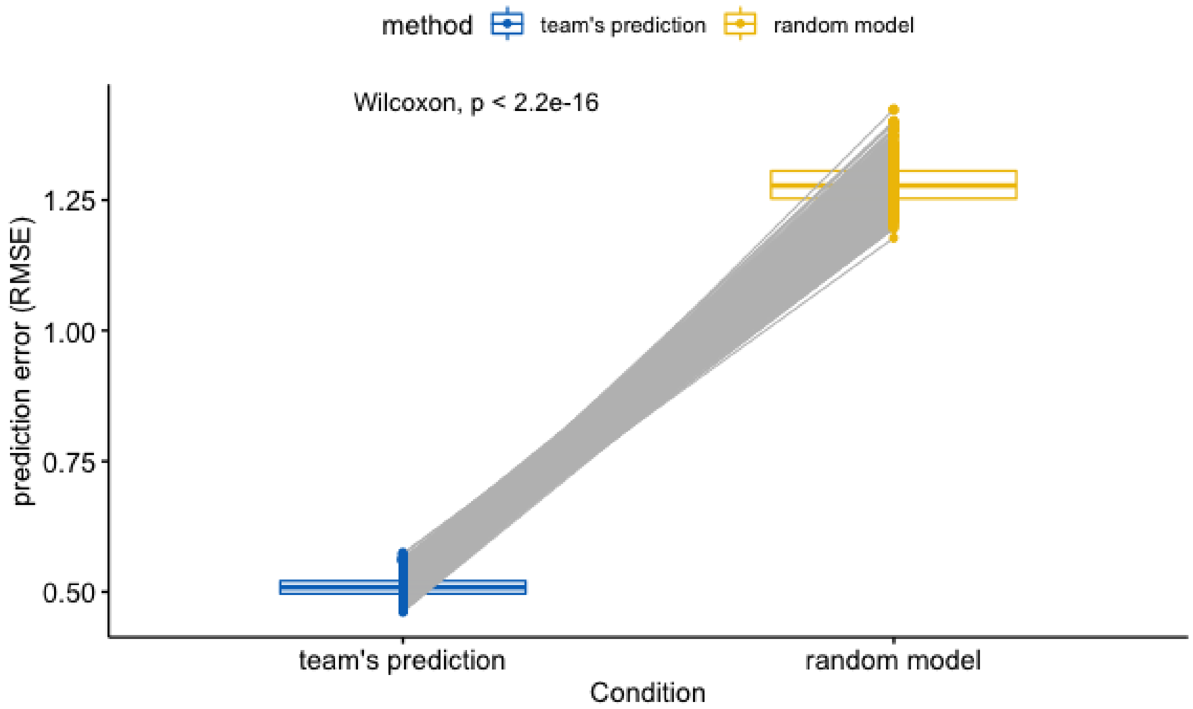

Appendix Figure S8. Comparison of team's prediction with random predictions on an independent dataset.
We compared the predictions on 1000 bootstrap (paired) samples and compared the distribution of the prediction errors by Wilcoxon signed ranked test), which shows strong (reduction in error: 0.771) and significant (p.val< 1e-16) difference in favour of the teams prediction.

We included the following text in the results section:

"We evaluated the winner's team method on a similar, but independent mass cytometry dataset (Lun et al. 2017), where the authors studied the differences in signaling dynamics due to node abundance (Appendix section "Single cell prediction on independent dataset"). We used the winner's method to learn signaling relationships among the measured markers in a set of cells overexpressing some proteins and then predict missing markers on cells expressing other proteins (Appendix Table S7). We found that the challenge winner's method greatly outperformed a random prediction ($RMSE_{team,Lun}$=0.508 vs $RMSE_{random,Lun}$ = 1.298, Appendix Figure S8), which demonstrates the applicability of the method on other datasets. "

Further, we added to the discussion:

“We tested the winner’s method on an independent mass cytometry dataset (Lun et al. 2017), where we found that the predictions are much better than random and the prediction error is smaller than what we observed in Subchallenge 1. This is likely because the “pseudo-cell lines” (cells that overexpress different proteins) are more similar to each other than the breast cancer cell lines. In any case, this result suggests that the method is applicable on other datasets.”

4) Finally the paper would benefit from more involvement of signaling network modelling groups and cell signaling labs and with different types of data. The authors are encouraged to develop this further.

We agree with the reviewer and we also hoped to see more participants from the signalling community. To enable the engagement post-challenge, data and code are freely available and hence any group can use both to benchmark their own methods.

Reviewer #2:

Gabor and Tognetti et al present a fascinating paper describing the recent results of Single Cell Signaling in Breast Cancer (SCSBrC) Dialogue for Reverse Engineering Assessments and Methods (DREAM) challenge. The study aims to leverage a vast single-cell training set to understand whether computational models can predict cell signaling responses in unmeasured (non-empirical) nodes. This is an important computational problem as existing single-cell proteomic technologies are limited by reporter channels (~50 in mass cytometry) or the availability well validated anti-PTM affinity reagents. If computational methods could accurately impute/predict unmeasured datapoints this could greatly expand our knowledge of signaling biology.

The paper is generally very well written and cleanly divided into 4 subchallenges (beautifully illustrated in Figure 1). Each subchallenge is clearly introduced and discussed. I thoroughly enjoyed reading the paper and have very few suggestions for improvement. This is an exceptional manuscript and I congratulate the authors on their excellent work.

We thank the reviewer for the kind words and we are glad that she/he enjoyed our paper.

Minor Points:

1) One important finding is that models combining multiple methods typically perform better than simpler alternatives. Is there some way to illustrate this when introducing each model (e.g. in Figure 2A)? The currently figure doesn't reveal ANY differences regarding the different models which hides this take-away message.

We agree that it is good to further represent the modeling approaches used by the teams. To make the differences among the approaches clearer for the reader, we included in the revised manuscript the description of the modelling approach of the top 3 teams for each sub-challenge.

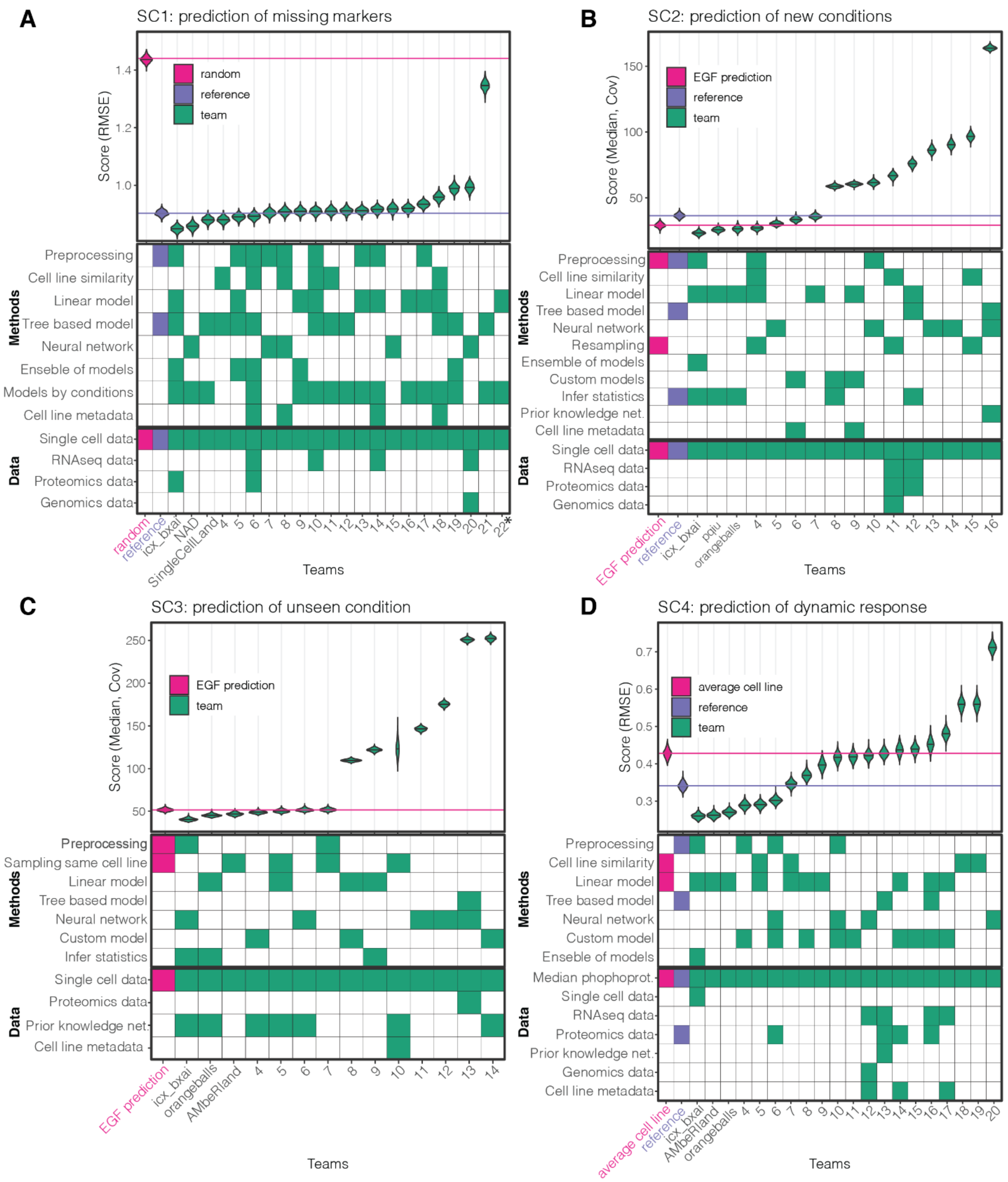

Figure 2. Summary of the ranking of teams and their methods and data usage across subchallenges. *Panel A, B, C, and D summarize subchallenge 1-4 respectively. The violin plots show the distribution of the scores obtained on bootstrap samples of the conditions in the test data. In all challenges lower scores mean better predictions. Reference models that we built before the challenge are highlighted with blue color, Random and null models (EGF condition based predictions and average cell line based predictions) are shown with magenta. Only data types that are used by at least one team are shown in each subchallenge. *Team 22 in SC1 scored worse than random and their score exceeds the limit of the y-axis.*

Of note, although the winning team combined multiple methods, this does not mean that this is the main reason why they won the challenges. As discussed in the results section, this team did many steps that others did not, for example they:

- subsampled the data to have a balanced number of cells in each condition
- carefully treated missing timepoints by interpolation
- engineered new features, e.g. by taking the quantiles of the single cell distributions.

Further, the second team in subchallenge I used a single algorithm, namely, convolutional neural network, which performed also very well. Combining the predictions of these teams, we didn't see much improvement of the scores (Appendix Figure S3C and S6).

2) What does the success of these models tell us about the underlying biology of signaling networks? Is the fact that signaling is predictable mean that signaling networks are highly ordered? Is the ultimate source of model failure cell-type-specific difference or non-canonically wired networks? Some discussion regarding the fundamental implications of these findings on signaling biology (i.e. not the models themselves) would be very valuable to readers.

We thank the reviewer for directing us to these questions. We have included the following paragraph to the discussion to address them:

"The activity of cellular markers within a single condition in a cell line could show 2-3 fold differences across single cells. The fact that models can explain up to 80% of this variance by the other measured markers suggests that despite the loose regulation between the cells, the intracellular signaling is highly ordered and the activity of the proteins are strongly connected. This phenomenon is already recognized and is used by network inference algorithms e.g. (Krishnaswamy et al. 2014). Furthermore, the models were trained on some cell lines and predicted the behaviour in other cell lines, which show that the learnt regulations are transferable between cell lines. However, our finding that certain rare signaling patterns were not captured well suggests that the transferred knowledge is limited and prediction algorithms should improve to capture rare cases."

3) Are there model types/formats that weren't used by contestants that theoretically might be better for future versions of such challenges? For example, 'machine learning' is frequently mentioned but not neural networks?

We agree with the reviewer that looking more into the methods used (and not used) is important. The participants used various methods in their predictions, which included linear models (ordinary least squares, ridge regression, regularized linear regression), (convolutional and recurrent-) neural networks

(e.g. team NAD, ranked 2nd in SC1), tree based algorithms (e.g. random forest, gradient boosting), and deep networks. We used the term “Machine Learning“ (ML) more often to keep things compact as Neural networks are a type of ML methods. Of note, we didn't see kinetic or mechanistic models of signaling networks.

We included the following paragraph in the discussion:

“The teams used various prediction algorithms, including linear models (ordinary least squares, ridge regression, regularized linear regression), (convolutional- and recurrent-) neural networks, tree based algorithms (e.g. random forest, gradient boosting), deep networks and custom differential equation based models (see Appendix Prediction methods of the best teams). However, similarly to previous challenges, we didn’t find evidence for the idea that a specific type of algorithm performed significantly better than other types of models for these prediction challenges. Across subchallenges, we found that the ensemble of predictions of multiple teams is better than an average team, which means that the risk for a bad prediction can be mitigated by combining multiple approaches. However, the combined prediction is not significantly better than the best team, which might be because the best team already used an ensemble of models.”

4) It is increasing appreciated that the cell-cycle has a huge effect on signaling networks. If all cell-cycle markers are removed for the training/test data, how do you think the models would have performed? Does this imply that cell-cycle markers are 'essential' elements of predicting signaling and should be included in all future signaling studies?

We agree with the reviewer that the effect of cell cycle on signaling is important.
As we showed in subchallenge 2 and 3, cell cycle markers drive the clustering of the single cells. Further, the following figure also shows that the cell-cycle state influences the dynamics of nodes: we gated MCF10A cells by cell cycle markers and then calculated the median of the markers in each conditions, then we plotted the p-AKT level in time in response to EGF+Serum activation and in combination with PI3K inhibitor.

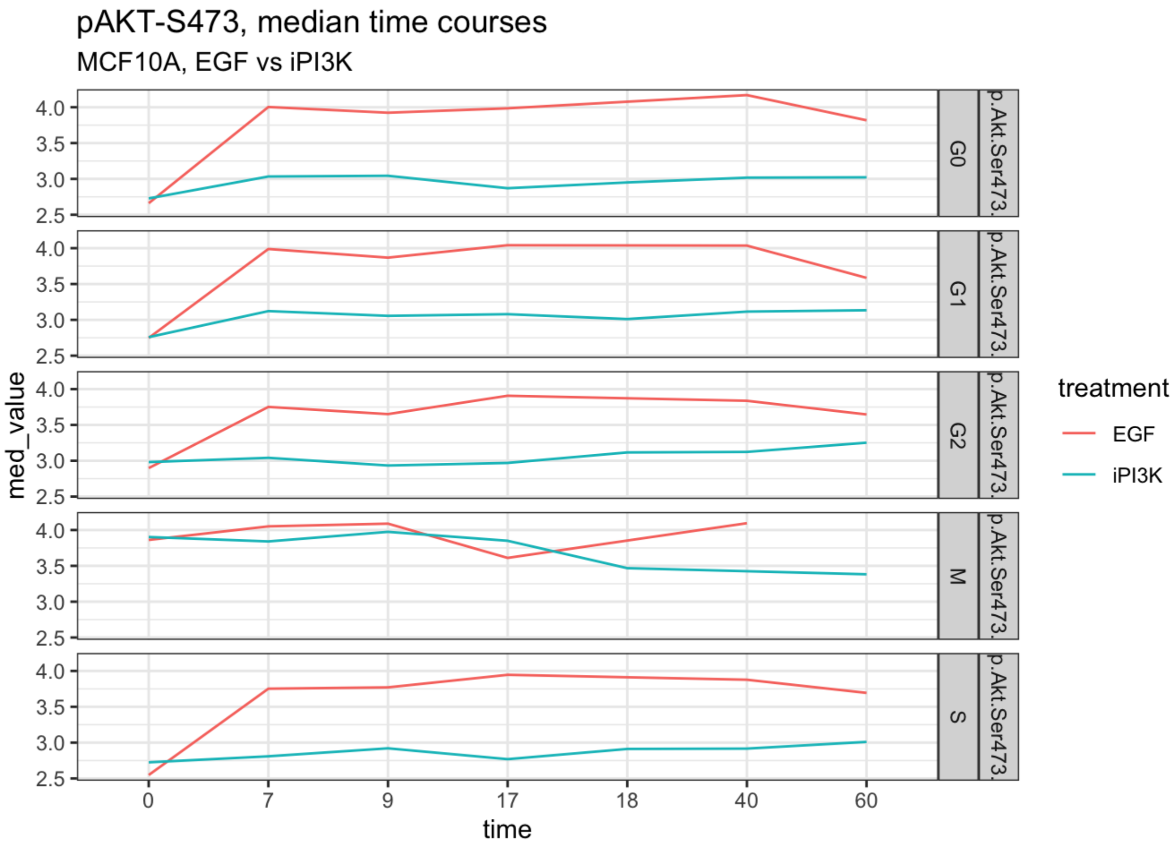

The median p-AKT response depends on Cell Cycle.
The response of cells of MCF10A cell line to EGF (red line) and EGF+iPI3K (cyan line) perturbation, in different cell cycle phases. In most cell cycle phases the response is similar: there is an increased phosphorylation of AKT in response to EGF stimulus, which reaches saturation in 7-9 mins. In the case of the PI3K inhibitor, the response is much lower. However, the response is quite different in M-phase: the initial phosphorylation of AKT is already high and the PI3K inhibition leads to a small decrease over time.

As we see in the above figure, cells in the M phase have a higher initial phosphorylation level and also respond differently to stimuli than cells in other states.

To put more emphasis on this issue in the revised manuscript, we extended the discussion by:

Further, in agreement with previous studies (Rapsomaniki et al. 2018; Behbehani et al. 2012), the data in subchallenge 2 and 3 show that some processes, like cell-cycle (Figure 5C), strongly influence signaling. It is important to include markers that either capture these processes, or are co-regulated by them.

Reviewer #3:

Gabor et al. and the Single Cell Signaling in Breast Cancer DREAM Consortium members reported the related DREAM challenge results. They use a large mass cytometry dataset that measures single cell dynamic signaling responses of 37 markers to EGF+"serum" over 1 hour across 67 breast cancer cell lines in the presence or absence of 5 different pathway inhibitors. In addition, they report "basal" proteomics (~5000 quantified proteins) and genomics (mRNAseq, CNV, SNP) data. The dataset alone is a substantial contribution to the literature, although the extent to which it is already available in the cited work was not clear. The challenge involving this dataset was split into four parts called subchallenges (SC). First, they asked participants to predict a subset of 5 markers for 6 cell lines, based on all the other data. The next two challenges asked participants to predict effects of inhibitors on the dynamic responses. In the 2nd, prediction was for 12 of the cell lines, given data for inhibitor response in the other cell lines. The prediction was only a single drug in each line, not all the drugs in each of the 12 lines. In the 3rd, the prediction was for a mTOR inhibitor, without having data on the mTOR inhibitor (besides the name and concentration), but using all the other data. The fourth challenge was to predict dynamics in response to EGF (plus serum) in 5 of the cell lines, given all the data, plus the "basal" data.
At the end of four sub-challenges (SCs), they conclude that the machine learning methods, especially a collection of them, can accurately predict single-cell perturbation responses. As denoted in their paper, there are not enough resources to do all the desired experiments or the current knowledge to intelligently select the most informative ones. Efforts like the one presented here, with accompanying datasets, will benefit and enable the biomedical research community in this regard greatly. In particular, the result that the cell-line specific signaling dynamics (at least over 1 hr time course) can be predicted from "basal" information is particularly compelling.

We would like to thank the reviewer for the detailed evaluation of the manuscript and appreciating the value of studies like this for the community.

That being said, I do have substantial concerns about the manuscript in its current form, that are detailed below.

Major Points:
1. An early summary figure of teams, their methodologies, and overall performances across different SCs will benefit the readers and make the text easier to understand before going into the Results section.

We agree with the reviewer that distributing the results of the subchallenges across multiple panels of figures might cause some confusion, therefore we collected the figures showing the scores of the teams on a single panel (new Figure 2; see also in this document on page 13, answer to Reviewer 1, minor point 1).

Further, to provide a summary of the used methodologies we extended the figures to indicate the approaches of the teams and datasets used. In case the reader would like to go to further details, we included the detailed methodologies of the top 3 teams of each subchallenge in the Appendix.

Importantly, the participants were allowed to use prior knowledge, but how that was implemented and/or impactful was missing from the paper.

We agree that the use of prior knowledge is an important point and it should be further discussed. We didn't restrict the participant to use any other type of data. Some teams used the protein-protein interaction network that we provided. For example, they used it to guess which other inhibitor could mimic the effect of the mTOR inhibitor in SC3 or which nodes could be influenced by an inhibitor in SC2. Based on the survey and the write-ups of the teams, the participants didn't use any other external datasets. We now elaborate on this in the discussion:

"Only some teams used the prior knowledge we provided, and none added any further external prior knowledge. Teams that did use this knowledge did not perform better, in contrast to the previous network inference challenge (Hill et al. 2016). In fact, top performing teams did not use prior knowledge, except the best performing team that used the prior network to predict the effect of mTOR inhibition on other nodes in Subchallenge 3. While the value of prior knowledge is considered helpful for network inference, it is not trivial to embed this in general machine learning frameworks, which could explain why it was not particularly relevant in this challenge."

2. The experimental methods could not be located in the paper. A detailed methods section is absolutely essential since a major contribution of this paper is the dataset (depending on the cited work).

We agree with the reviewer that we didn't give enough details about the experimental conditions in the manuscript, therefore we added a section to the Methods, which describes the datasets in more detail:

"Experimental data used in the challenge

In the challenge, the participants were provided with 3 main datasets (summarised by Appendix Figure S2): (1) the single cell phosphoproteomics data and median intensities of the phosphoproteomics data, (2) protein abundance data and (3) genomics data: RNA sequencing data, copy number variation (CNV) and single nucleotide polymorphism (SNP). The raw data and the experimental protocol for (1) and (2) are described in (Tognetti et al. 2021) and for (3) in (Marcotte et al. 2016). In what follows, we summarise the experimental detail that is relevant to the challenge.

The single cell phosphoproteomics dataset comprises the measurement of 62 breast cancer and 5 normal cell lines in six treatment conditions. The treatment conditions included stimuli with EGF and serum after 24 hours of starvation and stimuli with EGF and Serum after 24 hours starvation and 15 minutes incubation with one of the five kinase inhibitors. The kinase inhibitors and their concencentrations are listed in Appendix Table S3. Detachment occurred always 5 minutes prior to fixation. The response of the cells were measured at 7-10 time points in the first hour after treatment (Appendix Figure S2A). In each condition, 36 markers were measured by mass cytometry, which included 31 phosphoproteins and 5 cellular markers.

The protein abundance data were measured at normal growth condition in basal state. Participants were provided by both the raw proteomics data, which contained biological and technical replicates, and the protein abundance output from MSstat, which provides the relative quantification (log2 fold changes) to the set of five cell lines that were derived from healthy patients (184A1, 184B5, MCF10A, MCF10F, MCF12A).

The RNAseq, CNV and SNP data sets were generated and published (Marcotte et al. 2016) and have information for 64 of the characterized 67 cell lines."

3. Along the previous lines, it seems that the authors treated cells with EGF + serum (based on supplementary figure 2). The paper continually refers to EGF treatment. This discrepancy needs to be resolved. I could not decipher what was the case because I could not find detailed experimental methods.

We clarified now this in the introduction:

"In this dataset, 67 cell lines were stimulated with EGF and serum (referred hereafter as EGF treatment) in combination with one of five kinase inhibitors (PKC, PI3K, mTOR, MEK, EGFR)"

Further, we explain in more detail in the new Method section as mentioned in the previous point.

4. Another methodological point. Cytof requires suspension cells, but these all seem to be adherent. That needs to be reported and discussed. Adhesion often contributes strongly to signaling.

This is a valid point. Indeed these cells are adhesive. Detachment occurred always 5 minutes prior to fixation. The effect of detachment is expected to increase the baseline level of certain pathways, but this effect is constant across all experiments and is not expected to impact significantly time dependent effects.
We included this information in the Methods:

The adhesive cells were detached 5 minutes prior to fixation. The effect of detachment is expected to increase the baseline level of certain pathways, but this effect is constant across all experiments and is not expected to impact significantly time dependent effects.

5. I am not convinced by the authors claim that non-canonical MEK-independent ERK signaling can explain why models do not predict ERK signaling well in certain cell lines. The main reason are the data presented in Supplementary Figure 4, along with Supplementary Table 3. In the six cell lines shown in Fig. S4, the MEK inhibitor seems to have quite incomplete effects on pERK dynamics. Table S3 shows a non-clinical MEK inhibitor is used, at an abnormally high concentration for MEK inhibitors. Therefore it may be that issues with the inhibitor dose and / or the inhibitor itself may account for the observations. That being said, the paper is strong without going into details on MEK-independent ERK signaling. But, I do believe a rewording of all such claims in the paper is warranted. Likewise, it seems as though some of the other inhibitors may not be working as well as the authors may think.

We thank the reviewer for providing us a sensible alternative explanation for this signaling phenomenon, that is also a general limitation of using inhibitors. We reworded our claims and included the suggested explanation in the discussion:

"The second pitfall is related to rare signaling cases: in the majority of cell lines MEK phosphorylated ERK and the inhibition of MEK resulted in the lower phosphorylation of ERK. However, in a few cell lines, ERK showed a similar level of phosphorylation to EGF and Serum with and without the MEK inhibitor CI-1040. This phenomenon was not captured by the predictions either because the number of such cell lines were low in the training data or because there was no information on this alternative relationship in

the data, for example, if this phenomenon depends on the expression of non-measured nodes. It might also be that those cell lines showed this behaviour because the high concentration of MEK inhibitor resulted in cell-type-specific off-target effects. This example illustrates the potential limitations of inhibitors for network inference, as off-target effects can confound the results.“

6) Along the lines of the above point, on pg 14, the authors state "This relatively good score is due to the fact that the kinase inhibitors are very specific and influence only a small number of nodes". I am not sure why the authors are making this statement, but it is quite well established that kinase inhibitors show significant promiscuity, especially at some doses reported in Table S3.

We agree that our wording was not adequate. In this context we referred to the fact that we only saw phosphorylation effects on some markers in response to the inhibitors (figure 4A). We agree that drugs have generally off-target effects that need to be taken into account (as discussed in the previous point). We rewrote this sentence and now state that this observation only applies to the measured signaling node in the revised manuscript:

“This relatively good score could be due to the fact that each inhibitor showed strong effect only on a few nodes in the measured part of the signaling network.”

7) The dense time points chosen within the first hour of treatment response may not be indicative of the eventual phenotypic response such as cell cycle or cell death. The work remains significant, yet some discussion of the relation between dynamics, signaling and the phenotypes that are actually of final interest to predict is lacking.

Indeed as the reviewer points, this work focuses on early response, taking advantage of this unique data set. How early response relate to late events and phenotype is a valid related question, and we elaborate on this in the discussion of the paper:

“In this challenge we focused on predicting early signaling responses in breast cancer. Signaling responses have been used for predicting long term phenotypic changes, such as drug response. Bulk signaling response was used to successfully predict long term drug response (Niepel et al. 2013) and dynamic pathway models derived from signaling responses to perturbations predicted single and combined treatments in cancer cell lines (Korkut et al. 2015; Tognetti et al. 2021; Eduati et al. 2017; Silverbush et al. 2017). An

interesting prospect for future challenges would thus be to predict long term phenotypes from cell signaling at the single cell level, which can provide important insights into the mechanisms of drug resistance."

8) The claims of the paper on the surface seem quite significant, related to the fact that signaling dynamics and their inhibitor responses can be predicted reasonably well on a large scale. Yet, there are some aspects of this that I find somewhat lacking. For example, usually a small % of the dataset is predicted-in SC1 5 markers in 6 cell lines out of 37 markers and 67 cell lines, with "prior knowledge" allowed (what this means is opaque-see above comment).

There is indeed a limitation in the percentage of the dataset we can use for testing. For example, for Subchallenge I, some cell lines the measurement of PLCg2 and Her2 markers didn't pass the quality control and we could not use the values in the downstream analysis (Tognetti et al. 2021). Further, we needed to select a reasonably high number of cell lines for testing (as you also pointed out in comment 9, below). However, the more cell lines we would use in SC1, the less would be available for training the models. We thus decided to keep 10 % of the cell lines for validation as a trade-off.

What is the underlying dimensionality of the datasets (e.g simple PCA)? Is this surprising from a statistical or information perspective?

We thank the reviewer for this question. We did a PCA analysis of the test data (37 markers, 2.38M cells). The plot below shows the variance explained by the principal components. We need 22 principal components to explain 90.2% of the variance, suggesting that that variability of the data is complex and thus can not be trivially predicted.

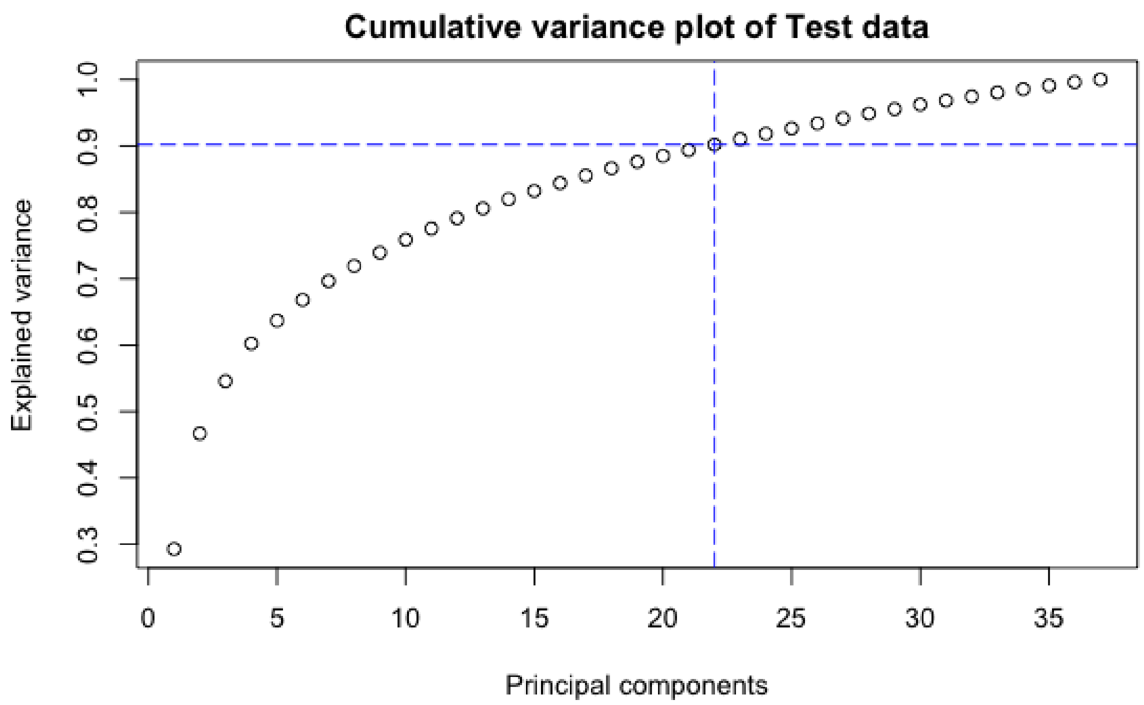

Figure rev1: PCA of the single cell data

From a biological perspective, the cytof panel is covering multiple parts of pathways that are expected to be highly correlated. For example, if I know what pMEK is doing, it is highly likely I know what pERK is doing. That kind of analysis and discussion is what I am referring to.

Indeed, the panel covers multiple parts of related pathways and, in our opinion, this structure of the data is what allows us to build models that can predict the missing nodes. We also agree that knowing pMEK provides some information on pERK, but the relation is not perfect (Figure rev2, below). If we check the range of correlation coefficients between these makers (Figure rev3, below), we can see that the correlation is high in most conditions but can be as low as 0.3 in others. This moderate connection among the measured markers are also reflected by the above PCA results, i.e. high collinearity would result in a few principal components explaining most of the variance, which is not the case.

We agree, that these points were not well explained in the manuscript, and we therefore added a paragraph on the biological interpretation of the prediction results to the discussion (see also reviewer#2, minor point 2):

"The activity of cellular markers within a single condition in a cell line could show 2-3 fold differences across single cells. The fact that models can explain up to 80% of this variance by the other measured markers shows that despite the loose regulation between the cells, the intracellular signaling is highly ordered and the activity of the proteins are strongly connected. This phenomenon is already recognized and is used by network

inference algorithms e.g. [(Krishnaswamy et al. 2014)](). Furthermore, the models were trained on some cell lines and predicted the behaviour in other cell lines, which show that the learnt regulations are transferable between cell lines. However, our finding that certain rare signaling patterns were not captured well shows that the transferred knowledge is limited and prediction algorithms should improve to capture rare cases."

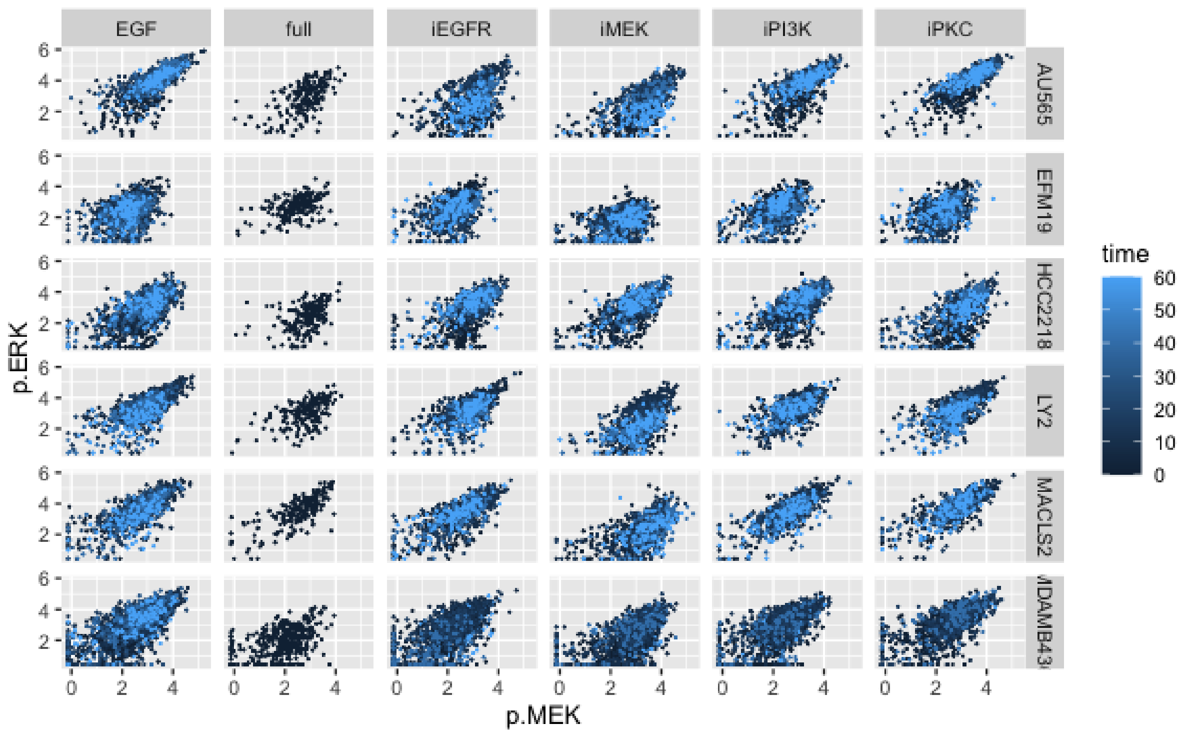

Figure rev2: Correlation of p.MEK and p.ERK across cell lines and treatments. Data was downsampled to 100 cells in each condition.

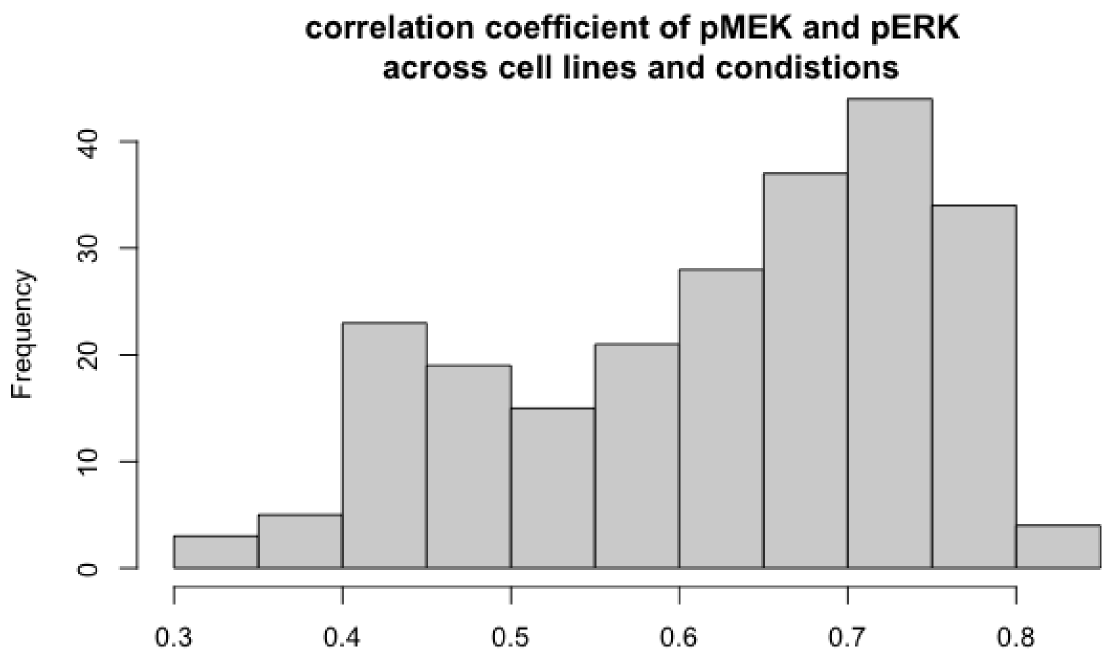

Figure rev3: The distribution of correlation coefficient between p.MEK and p.ERK across cell lines and conditions.

9) Along the above lines, usually test and training data are parsed many times over to get a real estimate of predictability. Granted, that is not really possible in this DREAM challenge scenario, but the lack of it diminishes the claims of how predictable things are.

We agree with the reviewer, that the more data we have the more accurately the prediction accuracy can be determined, and that one ideally would have independent datasets.
To overcome this limitation, we applied bootstrapping, which is a common statistical tool to test robustness, to evaluate the predictions.
Further, in the revised manuscript we included a new dataset from (Lun et al. 2017) and repeated the prediction tasks from SC1. All the details are summarised in Appendix "Single cell prediction on an independent dataset".

10) There is some hype with respect to the single-cell nature of this dataset and prediction. But the only true single-cell predictions seem to be in SC1. The remaining challenges focus on predicting mean and/or variance of the population. That discussion should be more balanced.

The reviewer is correct that only the first subchallenge evaluates actual single-cell predictions. The others use diverse population-based metrics, but some of them (co-variance of markers) can not be captured from bulk data, only from single-cell data.
We chose population-based metrics, because teams predicted all the markers of the single cells, and therefore it was impossible to map the predicted single cells to the measured single cells 1-to-1, like in SC1. Therefore we chose the means and the covariance matrix to make it possible to score teams while ensuring that the models had to capture cell-to-cell variability. We now clarify this distinction in the corresponding results section and in the discussion.

11) The authors' conclusion that the time resolution (or the number of time points) can be sacrificed first to reduce the number of experiments is not that well supported yet. Per their data and accompanying performance of the teams' models to predict time points better do not necessarily mean that the dynamical data are of lesser importance compared to more cell lines or more conditions (perturbations) in each cell line. One reason is that they also point out multiple times that the models predicted conditions of non-time-varying markers across cell lines better compared to other conditions. So, I believe without an analysis where the authors iteratively train and test model predictions at different time resolutions, their claim on this matter is not well supported.

We agree with the reviewer that further analysis should be made, and we have removed this statement.

12) to better understand what was done in this manuscript, the information in supplementary figure 2 would be quite useful in figure 1. The proportions in the cartoons of the 2nd row of figure 1 are not representative of what was done.

We agree with the reviewer. We have added numbers to Figure 1 to improve the information content and adjusted the proportions of the figures:

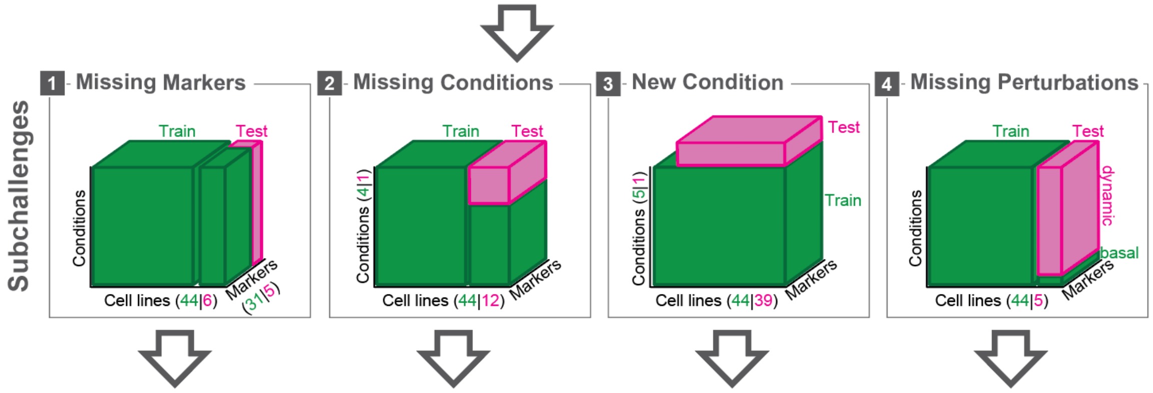

.

13) A major finding of this paper is the claim that signaling dynamics can be predicted from basal information. Some of that data is presented in Figure 5D. While there are cases where the predictions are good, there are other cases where the predictions seem quite wrong. So it is unclear how supported that claim is.

As the reviewer notes, the prediction is good for some but not all cases. We found these results still promisingly good, given the complexity of the task - predicting dynamics from static data is notoriously hard. At the onset of the challenge, we were not even sure that teams would be able to perform better than random.
We agree with the reviewer that the claims should be toned down, and we have done so in the key points, introduction, and discussion.

14) The comparison of model predictions to biological replicate variance may be overstated. Mostly because there were only the two replicates (from what I could see), the emphasis here seemed distracting in the flow of the paper.

We highlighted the replicates because it is the closest information to an actual ground truth we could find. We agree that this has to be taken with caution, given that we only have two replicates. We note this now in the discussion.

15) In the discussion (2nd paragraph), the authors speak to the transferability of inferred reactions / connections between cell lines. This is biologically not surprising, but a further discussion on how one could determine which signaling interactions are more transferrable than others would be biologically useful.

This is a good point and we have now added some thoughts on that paragraph:

"This implicitly assumes that the relationship among the nodes can be transferred between cell lines. This, in turn, could reflect common molecular programmes driven by the shared lineage of the cell lines. We would thus expect less transferability when considering different tissues or processes that are less linked to each other than the ones considered here."

Minor Points:
--The figure quality could be improved. Often times text is very small, and/or "stretched".
--pg 9. "maker"marker

--lapatinib is a dual EGFR/HER2 inhibitor, not an EGFR inhibitor.

Thank you for spotting these. We increased the figure quality, every figure is in vector graphics format in the manuscript and all fonts are at least 8pt (it was 6pt before). We fixed spelling and inhibitor target, too.

1st Revision - Editorial Decision 15th Sep 2021

Thank you for sending us your revised manuscript. We have now heard back from the two reviewers who were asked to evaluate your revised study. As you will see below, the reviewers are satisfied with the performed revisions and are supportive of publication.

Before we formally accept the study for publication, we would ask you to address some remaining editorial issues listed below.

REFEREE REPORTS

Reviewer #1:

Publish.

Reviewer #3:

The authors have done a very nice and thorough job at improving the quality of the manuscript describing this interesting and impactful work. I am happy to recommend publication.

2nd Authors' Response to Reviewers 27th Sep 2021

The authors have made all requested editorial changes.

Thank you for sending us your revised manuscript. We are now satisfied with the modifications made and I am pleased to inform you that your paper has been accepted for publication.

EMBO PRESS

YOU MUST COMPLETE ALL CELLS WITH A PINK BACKGROUND ➔

PLEASE NOTE THAT THIS CHECKLIST WILL BE PUBLISHED ALONGSIDE YOUR PAPER

Corresponding Author Name: Julio Saez-Rodriguez
Journal Submitted to: Molecular Systems Biology
Manuscript Number: MSB-2021-10402

USEFUL LINKS FOR COMPLETING THIS FORM

http://www.antibodypedia.com
http://1degreebio.org
http://www.equator-network.org/reporting-guidelines/improving-bioscience-research-repor
http://grants.nih.gov/grants/olaw/olaw.htm
http://www.mrc.ac.uk/Ourresearch/Ethicsresearchguidance/Useofanimals/index.htm
http://ClinicalTrials.gov
http://www.consort-statement.org
http://www.consort-statement.org/checklists/view/32-consort/66-title
http://www.equator-network.org/reporting-guidelines/reporting-recommendations-for-tumo
http://datadryad.org
http://figshare.com
http://www.ncbi.nlm.nih.gov/gap
http://www.ebi.ac.uk/ega
http://biomodels.net/
http://biomodels.net/miriam/
http://jjj.biochem.sun.ac.za
https://osp.od.nih.gov/biosafety-biosecurity-and-emerging-biotechnology/
http://www.selectagents.gov/

Reporting Checklist For Life Sciences Articles (Rev. June 2017)

This checklist is used to ensure good reporting standards and to improve the reproducibility of published results. These guidelines are consistent with the Principles and Guidelines for Reporting Preclinical Research issued by the NIH in 2014. Please follow the journal's authorship guidelines in preparing your manuscript.

A- Figures
1. Data
The data shown in figures should satisfy the following conditions:

- ➔ the data were obtained and processed according to the field's best practice and are presented to reflect the results of the experiments in an accurate and unbiased manner.
- ➔ figure panels include only data points, measurements or observations that can be compared to each other in a scientifically meaningful way.
- ➔ graphs include clearly labeled error bars for independent experiments and sample sizes. Unless justified, error bars should not be shown for technical replicates.
- ➔ if n< 5, the individual data points from each experiment should be plotted and any statistical test employed should be justified
- ➔ Source Data should be included to report the data underlying graphs. Please follow the guidelines set out in the author ship guidelines on Data Presentation.

2. Captions
Each figure caption should contain the following information, for each panel where they are relevant:

- ➔ a specification of the experimental system investigated (eg cell line, species name).
- ➔ the assay(s) and method(s) used to carry out the reported observations and measurements
- ➔ an explicit mention of the biological and chemical entity(ies) that are being measured.
- ➔ an explicit mention of the biological and chemical entity(ies) that are altered/varied/perturbed in a controlled manner.

- ➔ the exact sample size (n) for each experimental group/condition, given as a number, not a range;
- ➔ a description of the sample collection allowing the reader to understand whether the samples represent technical or biological replicates (including how many animals, litters, cultures, etc.).
- ➔ a statement of how many times the experiment shown was independently replicated in the laboratory.
- ➔ definitions of statistical methods and measures:
  - common tests, such as t-test (please specify whether paired vs. unpaired), simple χ2 tests, Wilcoxon and Mann-Whitney tests, can be unambiguously identified by name only, but more complex techniques should be described in the methods section;
  - are tests one-sided or two-sided?
  - are there adjustments for multiple comparisons?
  - exact statistical test results, e.g., P values = x but not P values < x;
  - definition of 'center values' as median or average;
  - definition of error bars as s.d. or s.e.m.

Any descriptions too long for the figure legend should be included in the methods section and/or with the source data.

In the pink boxes below, please ensure that the answers to the following questions are reported in the manuscript itself. Every question should be answered. If the question is not relevant to your research, please write NA (non applicable). We encourage you to include a specific subsection in the methods section for statistics, reagents, animal models and human subjects.

B- Statistics and general methods

Please fill out these boxes ➔ (Do not worry if you cannot see all your text once you press return)

1.a. How was the sample size chosen to ensure adequate power to detect a pre-specified effect size?	NA
1.b. For animal studies, include a statement about sample size estimate even if no statistical methods were used.	NA
2. Describe inclusion/exclusion criteria if samples or animals were excluded from the analysis. Were the criteria pre-established?	all data included
3. Were any steps taken to minimize the effects of subjective bias when allocating animals/samples to treatment (e.g. randomization procedure)? If yes, please describe.	NA
For animal studies, include a statement about randomization even if no randomization was used.	NA
4.a. Were any steps taken to minimize the effects of subjective bias during group allocation or/and when assessing results (e.g. blinding of the investigator)? If yes please describe.	yes, methods were scored based on metrics that we defined before the challenge
4.b. For animal studies, include a statement about blinding even if no blinding was done	NA
5. For every figure, are statistical tests justified as appropriate?	yes
Do the data meet the assumptions of the tests (e.g., normal distribution)? Describe any methods used to assess it.	We used non-parametric tests (Wilcoxon) where the assumption of normality was not correct
Is there an estimate of variation within each group of data?	Yes

Is the variance similar between the groups that are being statistically compared?	yes

C- Reagents

6. To show that antibodies were profiled for use in the system under study (assay and species), provide a citation, catalog number and/or clone number, supplementary information or reference to an antibody validation profile. e.g., Antibodypedia (see link list at top right), 1DegreeBio (see link list at top right).	NA, we used publicly available data.
7. Identify the source of cell lines and report if they were recently authenticated (e.g., by STR profiling) and tested for mycoplasma contamination.	cell line related information was reported in the referenced data sources

* for all hyperlinks, please see the table at the top right of the document

D- Animal Models

8. Report species, strain, gender, age of animals and genetic modification status where applicable. Please detail housing and husbandry conditions and the source of animals.	NA
9. For experiments involving live vertebrates, include a statement of compliance with ethical regulations and identify the committee(s) approving the experiments.	NA
10. We recommend consulting the ARRIVE guidelines (see link list at top right) (PLoS Biol. 8(6), e1000412, 2010) to ensure that other relevant aspects of animal studies are adequately reported. See author guidelines, under 'Reporting Guidelines'. See also: NIH (see link list at top right) and MRC (see link list at top right) recommendations. Please confirm compliance.	NA

E- Human Subjects

11. Identify the committee(s) approving the study protocol.	NA
12. Include a statement confirming that informed consent was obtained from all subjects and that the experiments conformed to the principles set out in the WMA Declaration of Helsinki and the Department of Health and Human Services Belmont Report.	NA
13. For publication of patient photos, include a statement confirming that consent to publish was obtained.	NA
14. Report any restrictions on the availability (and/or on the use) of human data or samples.	all data sources are reported in the manuscript
15. Report the clinical trial registration number (at ClinicalTrials.gov or equivalent), where applicable.	NA
16. For phase II and III randomized controlled trials, please refer to the CONSORT flow diagram (see link list at top right) and submit the CONSORT checklist (see link list at top right) with your submission. See author guidelines, under 'Reporting Guidelines'. Please confirm you have submitted this list.	NA
17. For tumor marker prognostic studies, we recommend that you follow the REMARK reporting guidelines (see link list at top right). See author guidelines, under 'Reporting Guidelines'. Please confirm you have followed these guidelines.	NA

F- Data Accessibility

18: Provide a "Data Availability" section at the end of the Materials & Methods, listing the accession codes for data generated in this study and deposited in a public database (e.g. RNA-Seq data: Gene Expression Omnibus GSE39462, Proteomics data: PRIDE PXD000208 etc.) Please refer to our author guidelines for 'Data Deposition'.<br><br>Data deposition in a public repository is mandatory for:<br>a. Protein, DNA and RNA sequences<br>b. Macromolecular structures<br>c. Crystallographic data for small molecules<br>d. Functional genomics data<br>e. Proteomics and molecular interactions	we used publicly available data, all sources are reported in the manuscript
19. Deposition is strongly recommended for any datasets that are central and integral to the study; please consider the journal's data policy. If no structured public repository exists for a given data type, we encourage the provision of datasets in the manuscript as a Supplementary Document (see author guidelines under 'Expanded View' or in unstructured repositories such as Dryad (see link list at top right) or Figshare (see link list at top right).	we used publicly available data, all sources are reported in the manuscript
20. Access to human clinical and genomic datasets should be provided with as few restrictions as possible while respecting ethical obligations to the patients and relevant medical and legal issues. If practically possible and compatible with the individual consent agreement used in the study, such data should be deposited in one of the major public access-controlled repositories such as dbGAP (see link list at top right) or EGA (see link list at top right).	NA
21. Computational models that are central and integral to a study should be shared without restrictions and provided in a machine-readable form. The relevant accession numbers or links should be provided. When possible, standardized format (SBML, CellML) should be used instead of scripts (e.g. MATLAB). Authors are strongly encouraged to follow the MIRIAM guidelines (see link list at top right) and deposit their model in a public database such as Biomodels (see link list at top right) or JWS Online (see link list at top right). If computer source code is provided with the paper, it should be deposited in a public repository or included in supplementary information.	

G- Dual use research of concern

22. Could your study fall under dual use research restrictions? Please check biosecurity documents (see link list at top right) and list of select agents and toxins (APHIS/CDC) (see link list at top right). According to our biosecurity guidelines, provide a statement only if it could.	Prediction data and analysis scripts are accessible via a CodeOcean capsule:<br>Single Cell Signaling in Breast Cancer DREAM challenge analysis, DOI:<br>https://doi.org/10.24433/CO.6078101.v2
